# Design and Synthesis of Novel Imidazo[4,5‐*c*]pyridine Derivatives, Evaluation of Their Activity Against Hepatitis C Virus and In Silico Prediction of Their Binding Mode to NS4B Protein

**DOI:** 10.1002/cmdc.70378

**Published:** 2026-07-13

**Authors:** Eftychia Karampella, George Mpekoulis, Maria Georgiou, Iris Kolida, Katerina I. Kalliampakou, Eirini P. Kollia, Eleni V. Mikropoulou, Maria Halabalaki, Christos T. Chasapis, Niki Vassilaki, Nikolaos Lougiakis

**Affiliations:** ^1^ Laboratory of Medicinal Chemistry Division of Pharmaceutical Chemistry Department of Pharmacy School of Health Sciences National and Kapodistrian University of Athens Athens Greece; ^2^ Molecular Virology Laboratory Hellenic Pasteur Institute Athens Greece; ^3^ Division of Pharmacognosy and Natural Products Chemistry Department of Pharmacy School of Health Sciences National and Kapodistrian University of Athens Athens Greece; ^4^ Laboratory of Organic Chemistry Department of Chemistry National and Kapodistrian University of Athens Athens Greece

**Keywords:** antiviral agents, drug design, hepatitis C virus (HCV), homology modeling, imidazopyridine, molecular docking, NS4B protein

## Abstract

A series of suitably substituted imidazo[4,5‐*c*]pyridine derivatives was synthesized, starting from 4‐amino‐2‐chloropyridine, and their antiviral activity against hepatitis C virus (HCV) genotype 1b (GT1b) was evaluated in Huh5.2 replicon cells. Some of the *N* ^3^‐substituted analogs exhibited potent anti‐HCV activity (EC_50_ = 6.61–47.24 μM), as determined by assessing their individual effects on viral RNA replication and protein expression levels. Among these, the most promising compound **30b** demonstrated synergistic antiviral activity when combined with clinically approved direct‐acting anti‐HCV therapies, highlighting its potential utility in combination regimens. These new derivatives were rationally designed to target the nonstructural 4B (NS4B) protein of HCV. Our molecular docking studies reinforced the implication of this mechanism of action, suggesting a binding mode similar to the previously reported and structurally related inhibitors clemizole and anguizole. A conserved hydrophobic pocket within NS4B (encompassing TM1–3, the Walker A motif, and the amphipathic helix H1) was identified, supporting the validity of this scaffold for further structure‐based optimization. Collectively, our results support further investigation of the imidazo[4,5‐*c*]pyridine scaffold for the rational development of novel anti‐HCV agents with potential application in combination therapies aimed at the effective elimination of chronic HCV infection.

## Graphical abstract

A series of 18 novel imidazo[4,5‐*c*]pyridines was synthesized, and their anti‐HCV activity was evaluated. Compound **30b** was identified as the most promising one, with EC_50_ = 6.61 µM. Molecular docking studies indicated the NS4B protein of the HCV as a potential target of these derivatives.

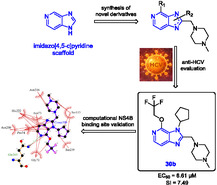

## 1 Introduction

Hepatitis C Virus (HCV) infection poses a major global health burden, affecting approximately 50 million people worldwide according to 2022 estimates [1, 2]. Disease severity can range from a mild, self‐limiting illness that resolves within a few weeks to a life‐threatening condition if left untreated [3, 4]. This outcome is largely attributed to the high rate of underdiagnosis, with less than 40% of the estimated affected population diagnosed [1, 2]. Unless treated, HCV infection can lead to chronic liver inflammation, potentially resulting in liver fibrosis, cirrhosis, hepatocellular carcinoma, and extrahepatic HCV‐related manifestations [5].

HCV is a single‐stranded, positive‐sense RNA ((+)ssRNA) virus, belonging to the *Hepacivirus* genus of the *Flaviviridae* family [6]. The HCV genome encodes a single polyprotein of approximately 3000 amino acids in length, which is subsequently proteolytically processed into 10 functional viral proteins: three structural proteins (the nucleocapsid core protein C, and the envelope glycoproteins E1 and E2), six nonstructural proteins (NS2, NS3, NS4A, NS4B, NS5A and NS5B) and the p7 viroporin [7]. Furthermore, an additional HCV protein has been identified, arising from translation of an alternative open reading frame, known as the frameshift (F) or alternative reading frame (ARF) protein. Notably, this protein is highly conserved across HCV genotypes [8]. Seven distinct HCV genotypes (genotypes 1–7) have been characterized, with this diversity driven by the high error rate of the viral RNA‐dependent RNA polymerase and selective pressure from the host immune system [9, 10].

Between 2001 and 2011, the standard of care for patients with chronic hepatitis C involved the combined administration of interferon analogs (PEG‐IFN‐α2a or PEG‐IFN‐α2b) together with the nucleoside analog Ribavirin (RBV). This therapeutic regimen showed variable efficacy across HCV genotypes and demographic groups, achieving a sustained virologic response in approximately 40%–80% of treated individuals [11, 12, 13], but was also associated with severe adverse effects [14]. In 2011, the first generation of drugs that directly target HCV viral proteins (Direct Acting Antivirals, DAAs) was introduced: boceprevir and telaprevir, two NS3/4A protease inhibitors. Since 2013, several DAAs against HCV have been approved by the FDA, broadly categorized into three main classes: NS3/4A protease inhibitors, NS5B RNA‐dependent RNA polymerase inhibitors, and NS5A protein inhibitors [15].

Combining DAAs enhances antiviral efficacy and shortens the treatment duration, which is critical for lowering the risk of selecting resistant mutants, a consequence of long‐term antiviral therapy [16]. DAAs can achieve sustained virologic response rates exceeding 90% in treated patients [17]. Nonetheless, the emergence of drug‐resistant variants remains a concern and is influenced by both the specific DAA regimen and the viral genotype [18]. The extensive genetic diversity of HCV [19] affects the genetic barrier to resistance, thereby complicating efforts to develop truly pangenotypic DAAs [20]. In addition, current DAA therapies are expensive, often costing several thousand euros per treatment, which restricts their accessibility and limits global treatment coverage. Consequently, there is still a pressing need for new antiviral agents that are both cost‐effective and possess a high barrier to resistance.

The nonstructural protein NS4B was identified early on as an attractive target for antiviral drug development based on multiple studies demonstrating its critical role in viral replication [21, 22, 23, 24]. Within infected cells, NS4B has been implicated in multiple functions, most notably in inducing the formation of specialized membrane structures known as the membranous web (MW), which serves as the site of viral replication [25, 26]. Consequently, inhibition of NS4B can disrupt the early stages of the HCV life cycle.

**FIGURE 1:**
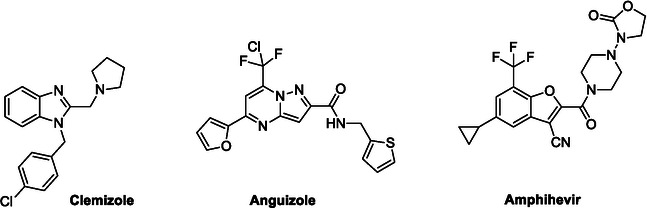
Chemical structures of the known NS4B inhibitors Clemizole, Anguizole and Amphihevir.

Among the first compounds that were reported to inhibit NS4B and suppress HCV replication were the hydrochloride salt of Clemizole, an H1 histamine receptor antagonist, and Anguizole, a substituted pyrazolo[1,5‐*a*]pyrimidine derivative (Figure 1) [27, 28]. Although several structurally related analogs of Anguizole, specifically those bearing the imidazo[1,2‐*a*]pyridine, pyrazolo[1,5‐*a*]pyridine or the indole scaffolds, have also been reported to possess significant anti‐HCV activity through NS4B inhibition [22], only one compound, the benzofuran derivative Amphihevir (Figure 1), has to date entered clinical trials [29].

**FIGURE 2:**
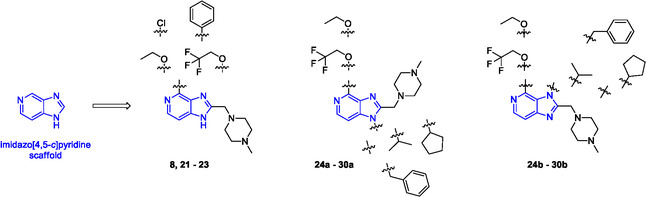
Structure of the novel derivatives of this study, bearing the imidazo[4,5‐*c*]pyridine scaffold.

During the past years, we have been actively involved in the synthesis and pharmacological evaluation of novel bioactive nitrogen‐containing derivatives [30, 31, 32, 33, 34], including compounds with potential antiviral activity [35, 36, 37, 38]. In this work, we synthesized a series of novel compounds bearing the imidazo[4,5‐*c*]pyridine central scaffold in order to evaluate their anti‐HCV activity. Based on the main structural features of Clemizole and Anguizole that appear to be essential for NS4B inhibition, we chose to incorporate in the new derivatives a *N*‐methylpiperazine side chain at position −2, through a methylene group, together with small‐sized substituents at position −4 of this scaffold (**8**, **21–23**, Figure 2). To investigate the effect of substituents on the nitrogen atoms of the imidazole ring on the antiviral activity, we also synthesized selected derivatives that have alkyl groups as substituents at the *N* ^1^ or *N* ^3^ positions (**24a**–**30a** and **24b**–**30b**, Figure 2).

In total, 18 novel target compounds were synthesized, and their anti‐HCV efficacy was evaluated. Additionally, the in silico prediction of their binding mode to the NS4B protein was performed.

## 2 Results and Discussion

### 2.1 Synthesis of the Novel Compounds

**SCHEME 1:**
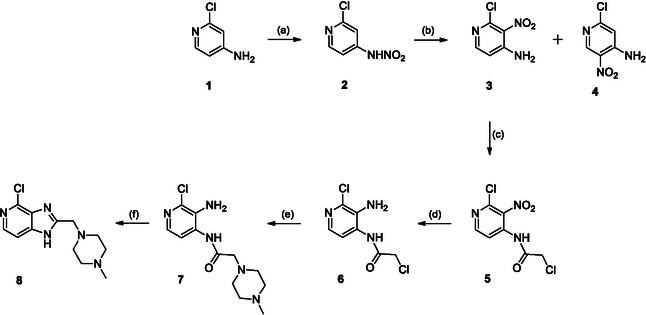
Reagents and conditions: (a) H_2_SO_4_, HNO_3_, r.t.; (b) H_2_SO_4_, 100 °C; (c) from **3**: chloroacetyl chloride, DMF (dry), r.t.; (d) H_2_, Raney nickel, EtOH (abs.), 45 psi, r.t.; (e) KI, *N*‐methylpiperazine, EtOH, 60 °C; (f) polyphosphoric acid, 100 °C.

For the synthesis of all the target derivatives, commercially available 4‐amino‐2‐chloropyridine (**1**, Scheme 1) was used as the starting material. Nitration of **1** provided the corresponding *N*‐nitramine **2**, which was converted to the mixture of regioisomeric nitro compounds **3** and **4** upon heating under acidic conditions [39]. This mixture was separated by column chromatography, and the two regioisomers **3** and **4** were isolated in 60% and 28% yield, respectively. The ^1^H‐NMR spectra and the melting points of compounds **3** and **4** were identical to those described in the literature [39]. The desired nitro derivative **3** was converted to the chloroacetamide **5**, and the nitro group of the latter was reduced by catalytic hydrogenation, resulting in the amine **6**. Raney nickel was selected as the catalyst in this step in order to avoid the simultaneous dehalogenation reaction of the chloroderivative **5** during the hydrogenation. Nucleophilic substitution of the chlorine atom of **6** by *N*‐methylpiperazine provided intermediate compound **7**, which was finally ring‐closed upon heating in polyphosphoric acid, to provide the 4‐chloroimidazopyridine target compound **8** in a moderate yield.

**SCHEME 2:**
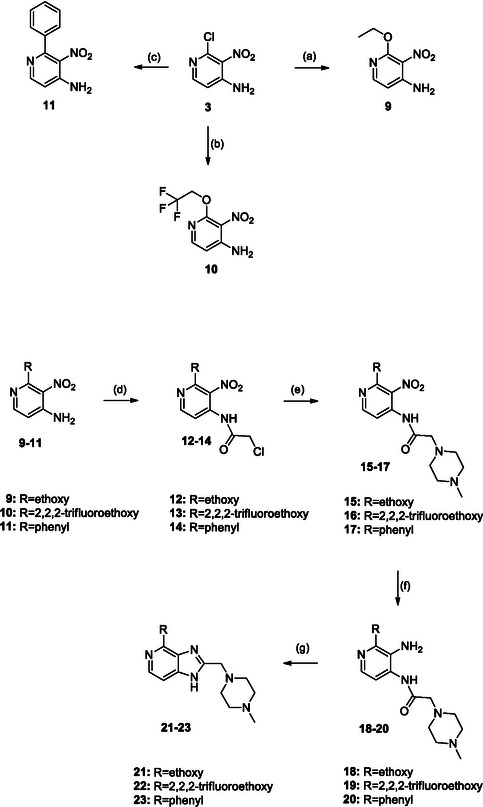
Reagents and conditions: (a) EtONa, EtOH, 75 °C; (b) 2,2,2‐trifluoroethanol, NaH, DMF (dry), 70 °C; (c) phenylboronic acid, Pd(PPh_3_)_4_, K_2_CO_3_, toluene, EtOH, H_2_O, reflux; (d) chloroacetyl chloride, DMF (dry), r.t.; (e) KI, *N*‐methylpiperazine, EtOH, 60 °C; (f) H_2_, Pd/C, EtOH (abs.), 54 psi, r.t.; (g) acetic acid, 100 °C.

The nitropyridine derivative **3** was also used for the preparation of the rest of the 4‐substituted imidazopyridines **21**–**23**, as depicted in Scheme 2. Thus, treatment of **3** with either sodium ethoxide, sodium 2,2,2‐trifluoroethoxide, or phenylboronic acid under Suzuki coupling reaction conditions resulted in the corresponding 2‐substituted‐4‐amino‐3‐nitropyridine compounds **9–** **11**, respectively. The signal of the methylene group of the 2,2,2‐trifluoroethoxy moiety for compound **10** appears in its ^1^H‐NMR spectrum as a quartet peak at 4.82 ppm, whereas in the ^13^C NMR spectrum of **10**, two quartets are observed, one for the methylene carbon at ∼63 ppm, and another one, more deshielded (at ∼123 ppm), for the carbon of the trifluoromethyl group. Reaction of **9–11** with chloroacetyl chloride led to the corresponding amides **12–14**, which upon treatment with *N*‐methylpiperazine yielded the piperazine acetylamides **15–17**. The nitro groups of the latter compounds were reduced by catalytic hydrogenation in the presence of palladium on carbon as the catalyst, resulting in the corresponding amines **18–20**, which were finally ring‐closed upon heating in acetic acid to provide the 4‐ethoxy, 4‐(2,2,2‐trifluoroethoxy), and 4‐phenyl‐substituted imidazopyridine target compounds **21–23**, respectively. The use of acetic acid instead of polyphosphoric acid for the formation of the imidazole ring significantly improved the yield of this reaction; thus, compounds **21**–**23** were isolated in higher yields (63%–93%) compared to that of the derivative **8**.

**SCHEME 3:**
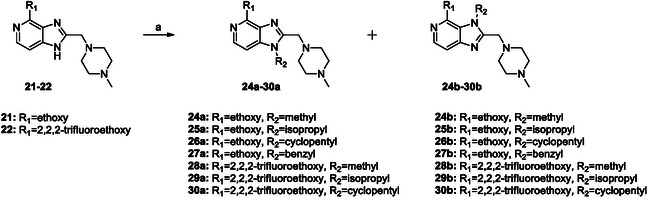
Reagents and conditions: (a) K_2_CO_3_, iodomethane (1.5 eq.) for **24a/24b** and **28a/28b**, or 2‐bromopropane (4 eq.) for **25a/25b** and **29a/29b**, or bromocyclopentane (4 eq.) for **26a/26b** and **30a/30b**, or benzyl bromide (1.5 eq.) for **27a/27b**, DMSO (dry), r.t.

Finally, the selected target derivatives substituted on *N* ^1^ or *N* ^3^ of the imidazole ring (compounds **24a–30a** and **24b–30b**, respectively, Scheme 3) were obtained from the imidazopyridines **21** and **22**, upon reaction with appropriate alkyl/cycloalkyl/benzyl halides, in the presence of potassium carbonate. It should be noted that in the case of the isopropyl and the cyclopentyl substituted derivatives (**25a**/**25b**, **26a**/**26b**, **29a**/**29b** and **30a**/**30b**), an excess of potassium carbonate and 2‐bromopropane or bromocyclopentane, respectively, was necessary since the secondary halides could also undergo an elimination reaction toward the corresponding alkenes.

The site of alkylation for each pair of isomers (*N* ^1^ or *N* ^3^ substituted) was unambiguously confirmed by nOe spectroscopy. Thus, for all the *N* ^1^ substituted isomers (compounds **24a–30a**), distinct correlation peaks were observed between the H‐7 proton of the pyridine ring with the corresponding protons of the alkyl/cycloalkyl/benzyl substituent, respectively. In contrast, such correlation peaks were not observed for their *N* ^3^ substituted counterparts (compounds **24b–30b**) in their nOe spectra. Copies of the ^1^H‐ and ^13^C NMR spectra of all the novel derivatives and representative NOESY spectra of the pairs of *N* ^1^ and *N* ^3^ substituted target compounds are provided in the supplementary material file (Figures S1 and S2, respectively).

### 2.2 Screening of Compounds for Antiviral Activity Against HCV Genotype 1b

The efficacy of the newly synthesized compounds toward HCV was experimentally assessed in Huh5−2 cells. These cells stably harbor the subgenomic HCV genotype 1b replicon encoding the nonstructural proteins required for RNA replication, along with the Renilla luciferase reporter gene for quantitative monitoring of replicon activity [40]. The clinically approved HCV‐NS5A inhibitor daclatasvir (DCV) was used in parallel to ensure the robust performance of the antiviral assays.

Prior to the antiviral assays, the cytotoxic effects of all synthesized derivatives were evaluated by determining their median cytotoxic concentrations (CC_50_), defined as the concentration at which each compound reduces cell viability by 50%.

Specifically, Huh5.2 cells were seeded in 6‐well plates and subsequently treated with serial concentrations of DMSO‐diluted compounds or mock‐treated (treated with the solvent DMSO). At 72 h post‐treatment, the viability of cells was determined using an assay that measures the metabolic activity of living cells. As shown in Table 1, only compound **30b** displayed moderate cytotoxicity, with a CC_50_ value of 49.49 μM.

**TABLE 1:** The EC_50_ and CC_50_ values of the analogs in Huh5.2 cell line harboring HCV Con1 replicon.

Compound	CC_50_, μM^a^	EC_50_, μM^b^	SI^c^
**8**	>200	>50	—
**21**	>200	>50	—
**22**	>200	>50	—
**23**	>200	>50	—
**24a**	>200	>50	—
**24b**	>200	>50	—
**25a**	>200	>50	—
**25b**	>200	36.15 ± 3.30	>5.6
**26a**	>200	>50	—
**26b**	>200	18.37 ± 1.22	>10.9
**27a**	>200	>50	—
**27b**	>200	>50	—
**28a**	>200	>50	—
**28b**	>200	>50	—
**29a**	>200	47.24 ± 2.03	>4.2
**29b**	109.1 ± 3.39	>50	—
**30a**	>200	>50	—
**30b**	49.49 ± 0.65	6.61 ± 0.98	7.49
**DCV**	>17.7	7.72 × 10^−6^	2.29 × 10^6^

a CC_50_ values (mean cytotoxic concentration).

b EC_50_ values (mean effective concentration) represent the mean ± SD of three independent experiments and were calculated against in Huh5‐2 cells (HCV‐GT1b replicon cells).

c Selectivity indices were evaluated by calculating the ratio of the CC_50_/EC_50_.

Next, the efficacy of the novel compounds against HCV genome replication was determined in Huh5.2 cells. In brief, cells were seeded in 96‐well plates at approximately 40% confluence, and 24 h later were treated with noncytotoxic concentrations of each analog, 72 h later the cells were lysed, and the activity of F‐luc was measured. For each compound, the median effective concentration (EC_50_), defined as the minimal concentration of the compound required to reduce viral replication by 50%, was determined. Notably, compound **30b** exerted the most significant antiviral potency against HCV, with an EC_50_ = 6.61 µM and a selectivity index (SI = CC_50_/EC_50_) value of 7.5. Moderate inhibition of viral replication was also observed in the presence of analogs **29a**, **25b** and **26b** (EC_50_ = 47.24, 36.15 and 18.37 μM respectively) (Table 1).

As presented in Table 1, compounds **8**, **21**, **22**, and **23** that are unsubstituted on the imidazole ring were inactive. The same observation was also made for all the *N* ^1^‐substituted derivatives (compounds **24a**–**30a**), except for the *N* ^1^‐isopropyl derivative **29a**, which exhibited an EC_50_ of 47.24 µM. In contrast, three *N* ^3^‐substituted derivatives (compounds **25b**, **26b**, and **30b**) showed promising antiviral activity, with the *N* ^3^‐cyclopentyl‐substituted analogs **26b** and **30b** being the most active within this series, displaying EC_50_ values of 18.37 and 6.61 µM, respectively.

Although the most potent analog, compound **30b**, displayed moderate cytotoxicity, it nevertheless exhibited a selectivity index (SI: CC_50_/EC_50_) of 7.49, suggesting a favorable safety profile. Overall, these data highlight the importance of *N* ^3^ alkylation versus *N* ^1^ alkylation on the imidazole ring in terms of anti‐HCV activity and suggest a potential contribution of hydrophobic interactions to the underlying mechanism of action.

### 2.3 Further Characterization of Compound 30b‐Mediated Inhibition of HCV Replication

**FIGURE 3:**
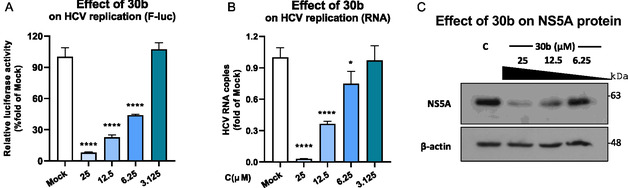
The inhibitory effect of compound 30b on HCV replication in Huh5.2 replicon cells. Huh5.2 cells were treated for 72 h with the indicated DMSO‐diluted drug concentrations (25, 12.5, 6.25 and 3.125 μM, respectively) or were mock‐treated (control), for 72 h. Then the cells were lysed. (A) Luciferase activity is shown as RLU/mg total protein. Data represent means ± SD from three independent experiments performed in triplicate. Statistical analysis was performed using one‐way ANOVA followed by Dunnett's post‐hoc test. ****p < 0.0001 versus control. (B) Levels of (+) strand HCV RNA as determined by RT‐qPCR in Huh5.2 cells treated with 25, 12.5, 6.25, and 3.125 μM of compound **30b**, respectively, or mock‐treated (control). The mRNA levels of the housekeeping gene YWHAZ were used for normalization. Bars represent the average values from three independent experiments, each performed in triplicate, with error bars showing the standard deviations. Statistical analysis was performed using one‐way ANOVA, followed by Dunnett's post‐hoc analysis for multiple comparisons. *p < 0.05, ****p < 0.0001 versus control. (C) Western blot analysis for the detection of NS5A protein. β‐actin was used as a loading control. Numbers on the right refer to the positions of molecular mass marker proteins.

**FIGURE 4:**
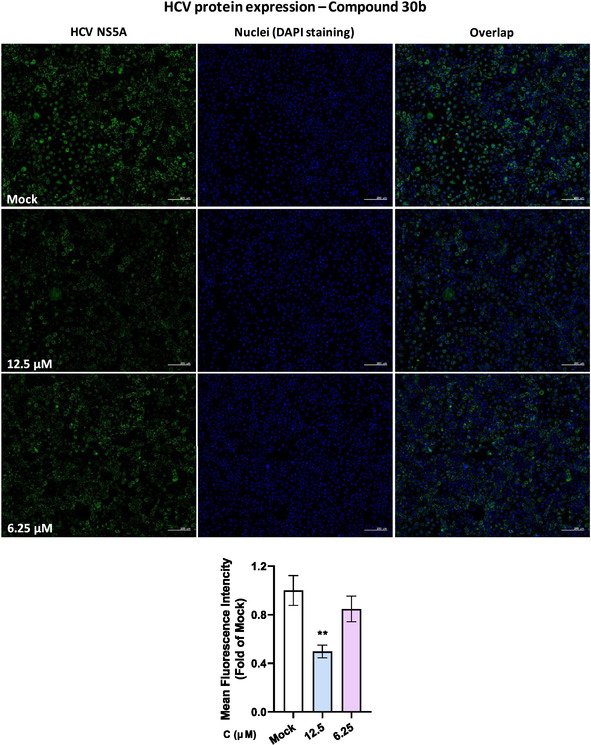
Compound 30b decreases HCV NS5A protein levels. Huh5.2 cells were treated for 72 h with the indicated DMSO‐diluted **30b** concentrations (12.5 or 6.25 μM, respectively) or were mock‐treated (control), for 72 h. Then, the cells were fixed. (Top) The presence of NS5A protein was detected by immunofluorescence (left panels). Nuclei were stained with DAPI (middle panels), and merged images are shown on the right (right panels). Scale bar, 200 μm. (Bottom) Alexa‐488 fluorescence (NS5A staining) was quantified and expressed as mean fluorescence intensity, normalized to the total cell count. The effect of compound **30b** on HCV replication was quantified by analyzing 10 distinct images collected from three independent experiments for each experimental condition. Statistical analysis was performed using one‐way ANOVA, followed by Dunnett's post‐hoc analysis. **p < 0.001 versus Mock‐treated cells.

The inhibitory activity of the most promising compound (**30b**) against HCV proliferation was further characterized by evaluating its effects on viral RNA and protein levels. Specifically, Huh5.2 cells were seeded in 6‐well plates and subsequently treated with serial noncytotoxic concentrations of DMSO‐diluted **30b** or mock‐treated (treated with the solvent DMSO) for 72 h. Then, total RNA was isolated, reverse transcribed, and viral RNA levels were determined by quantitative polymerase chain reaction (qPCR). Additionally, after cell protein extraction and quantification, the HCV NS5A protein levels were assessed by Western Blotting. The expression of HCV NS5A protein in Huh5.2 cells was also detected via immunofluorescence (IF). Consistent with the results obtained from the luciferase reporter assay, treatment of Huh5.2 cells with compound **30b** resulted in a pronounced reduction of both viral RNA (Figure 3B) and NS5A protein levels (Figures 3C and 4), compared to mock‐treated cells. Given that NS4B is a key organizer of the HCV replication organelle, it is plausible that functional inhibition of this protein by compound **30b** may disrupt the assembly or stability of the HCV replication complexes, thereby impairing HCV genome replication.

### 2.4 Combinatory Treatments of Compound 30b With Already Approved HCV Inhibitors

**FIGURE 5:**
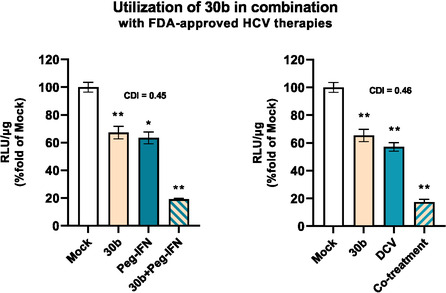
Treatment of Huh5.2 cells with 30b in combination with Peg‐IFN or DCV. Huh5.2 cells were treated or not with Peg‐IFN (0.3125 μM) or DCV (5 pM) in combination or not with **30b** (6.25 μM). The activity of Firefly Luciferase was expressed as RLU/mg of total protein levels. Bars indicate mean values derived from three independent experiments performed in triplicate. Error bars correspond to the standard deviation (SD). Statistical analysis across all panels was performed using one‐way ANOVA, followed by Dunnett's correction. *p < 0.001, ** *p* < 0.0001 compared to Mock‐treated cells.

To further investigate the antiviral potential of compound **30b** against HCV replication, its efficacy was evaluated in combination with pegylated interferon (peg‐IFN) [41] or daclatasvir (DCV) [42], two clinically approved anti‐HCV agents. Combination treatments were performed in Huh5.2 cells using concentrations of 6.25 μM for compound **30b**, 0.3125 μM for peg‐IFN, and 5 pM for DCV. As shown in Figure 5, co‐treatment with compound **30b** and either peg‐IFN or DCV resulted in a more pronounced suppression of viral replication compared with each agent administered alone. To quantify the interaction between the compounds, the coefficient of drug interaction (CDI) was calculated using the formula CDI = AB/(A × B), where A and B represent the antiviral effect of each compound individually and AB represents the antiviral effect when A and B are combined. As shown in Figure 5, the CDI values for the combinations of **30b** with peg‐IFN and DCV were 0.45 and 0.46, respectively, indicating synergistic interactions of **30b** with peg‐IFN and DCV, respectively. The observed synergies suggest that compound **30b** enhances the antiviral activity of both approved agents and are consistent with a mechanism of action distinct from that of daclatasvir. Collectively, these findings support that the core structure of compound **30b** constitutes a promising scaffold for the development of novel anti‐HCV agents suitable for combination therapy.

### 2.5 In Silico Study of the NS4B – Compound Interaction

The structure of the HCV NS4B protein can be functionally divided into three regions [22]: i) N‐terminal region: comprising amphipathic helix 1 (AH1, residues 6–29), followed by a loop (residues 30–41), amphipathic helix 2 (AH2, residues 42–66), and a short loop (residues 67–69) connecting AH2 to the first transmembrane domain. ii) Central core region: containing four predicted transmembrane domains (TM1–TM4; residues 70–190), separated by interhelical loops. Notably, the loop between TM2 and TM3 (residues 129–135) harbors the Walker A‐type nucleotide‐binding motif (NBM), which has been associated with NTPase activity [43]. iii) C‐terminal region: composed of a loop between TM4 and helix H1 (residues 191–199), amphipathic helix H1 (residues 200–213) that is involved in RNA binding, a loop connecting H1 and H2 (residues 214–228), and helix H2 (residues 229–253), with residues 247–248 also implicated in RNA interactions.

**FIGURE 6:**
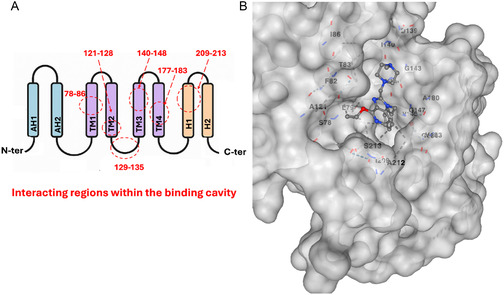
(A) Schematic representation of the interacting residues forming the binding cavity in the NS4B topology. (B) Docking pose of the top‐scoring compound **23** within the binding cavity of NS4B.

Blind docking of the newly synthesized compounds (Table S1) to the modeled NS4B structure consistently revealed a single dominant ligand‐binding site, located within a hydrophobic cavity in the central core of the protein. This binding cavity encompasses residues from multiple functionally critical elements, including TM1 (residues 78–86), TM2 (residues 121–128), the Walker A NBM (residues 129–135), TM3 (residues 140–148), and extends toward helix H1 (residues 200–213) in the C‐terminal region (Figure 6A).

**FIGURE 7:**
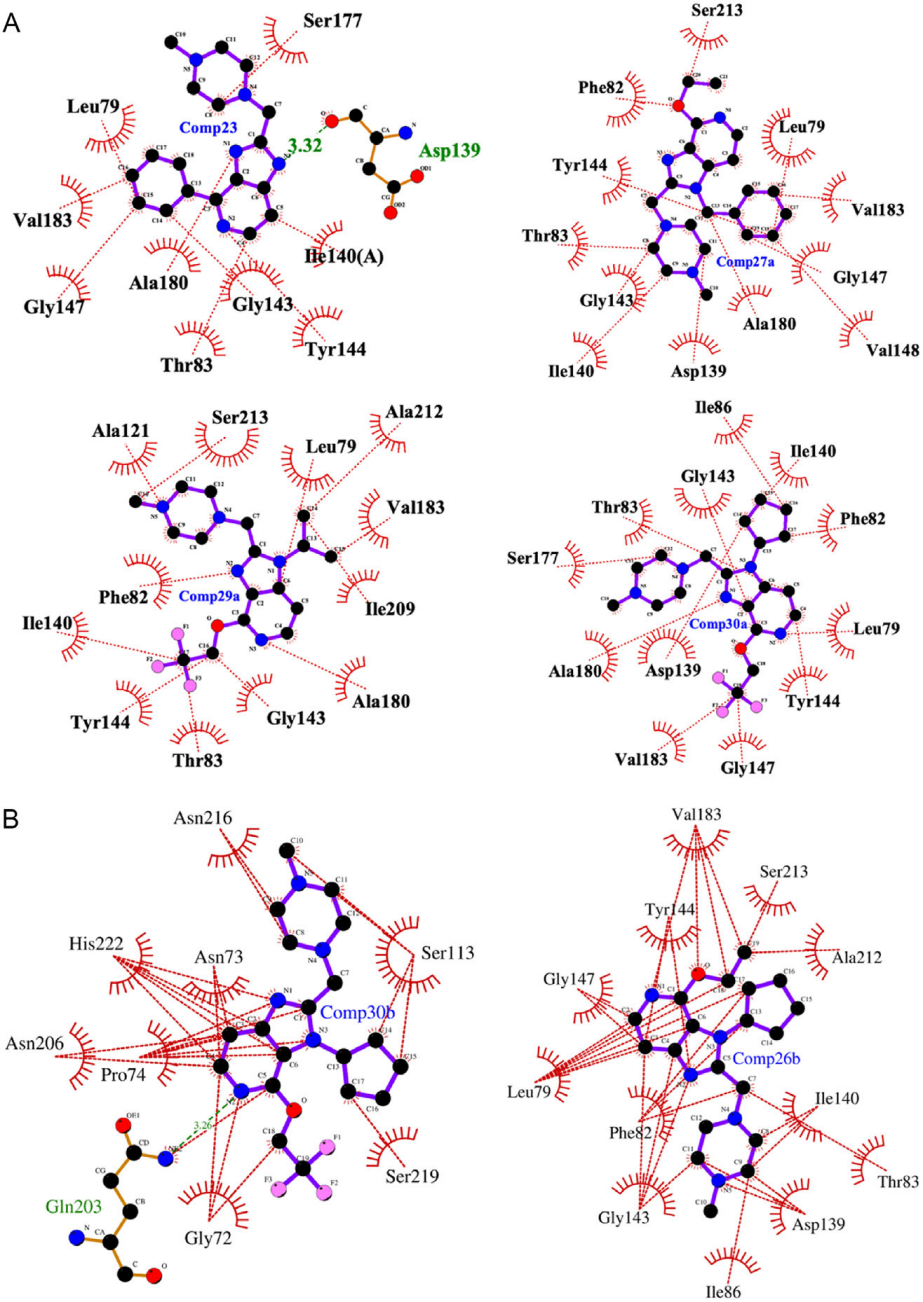
Close‐up view of (A) the top‐scoring compounds: **23**, **27a**, **29a**, and **30a**, and (B) the most active compounds **30b** and **26b**, in the highest‐scoring binding conformation of each compound with amino acid residues of the NS4B protein. The orientation of the ligands within the binding pocket is illustrated, along with key interactions with surrounding residues that contribute to binding stabilization. (Red dashed lines represent hydrophobic interactions, while green dashed lines indicate hydrogen bonds).

Docking simulations yielded binding scores ranging from −5.9 to −7.5 kcal/mol across the newly synthesized compounds (Table S1). The top‐scoring compounds, **23**, **30a**, **29a**, and **27a**, each showed a docking score of −7.5 kcal/mol, localizing stably within the binding cavity. These ligands established interactions with residues Ser78–Ile86 (TM1), Ala121–Val128 (TM2), Ile131–Val136 (Walker A motif), Asp139–Val148 (TM3), Ser177–Val183 (TM4) and Ile209–Ser213 (H1) (Figures 6B and 7). The binding score of these compounds was closely followed by that of **27b** (−7.4 kcal/mol), which showed a binding mode similar to its regioisomeric **27a**. Compound **29b**, exhibiting a docking score of −7.1 kcal/mol, was found to occupy the same cavity and to interact with most of the residues involved in the interaction of NS4B with **29a**. Compounds **28a**, **25b**, and **26a**, each with a docking score of −6.9 kcal/mol, maintained stable interactions within the binding site. In the case of **25a** (docking score of −6.8 kcal/mol), contacts were formed with conserved residues across the transmembrane core of NS4B. A similar interaction profile was exhibited for **28b**, with a docking score of −6.7 kcal/mol. Derivatives **22** and **24a** were also predicted to bind within the cavity, each with a docking score of −6.7 kcal/mol. Compounds **30b** (docking score of −6.6 kcal/mol), **26b** (docking score of −6.5 kcal/mol), and **24b** (docking score of −6.5 kcal/mol) exerted a moderate binding affinity within the same site of NS4B. Among the lowest‐scoring ligands, compound **8** was predicted to bind within the cavity with a score of −6.0 kcal/mol, while compound **21**, which theoretically exhibits the weakest binding to NS4B (docking score of −5.9 kcal/mol), was predicted to interact with amino acid residues located in the identified cavity of NS4B.

A summary of the main hydrogen‐bonding, aromatic and hydrophobic contacts for representative ligands (**23**, **27a**, **29a**, **30a**, **26b**, **30b**) is provided in Table S2, illustrating that higher docking scores are associated with increased hydrophobic complementarity and at least one hydrogen bond involving residues within the Walker A motif or TM1/TM3.

In parallel with the newly synthesized compounds, blind docking experiments were performed for three known NS4B‐targeting agents: amphihevir, anguizole, and clemizole, using the same structural model of NS4B. All three compounds exhibited higher or comparable binding affinities relative to the synthesized ligands. Specifically, amphihevir achieved a docking score of −9.4 kcal/mol, and anguizole scored −8.4 kcal/mol, both significantly stronger than the highest docking score observed among the newly synthesized ligands (−7.5 kcal/mol). Clemizole, with a score of −7.5 kcal/mol, matched the top‐ranked ligands. Notably, the highest‐scoring binding conformations of all three well‐known NS4B‐targeting agents were located within the same cavity identified for the newly synthesized ligands, suggesting a shared binding site of potential functional relevance (Figure S3).

Docking results showed that the ligands adopted overlapping binding modes, engaging residues from TM1 (78–86), TM2 (121–128), the Walker A motif (129–135), TM3 (140–148), and helix H1 (200–213). To ensure that this cavity is compatible with current knowledge on NS4B architecture, we compared the arrangement of the N‐terminal amphipathic helices (AH1–AH2), the four predicted transmembrane segments (TM1–TM4) and the C‐terminal helices H1–H2 in our ColabFold‐derived model with previously reported topology analyses and mutational data, which collectively support this overall organization rather than a fully resolved high‐resolution structure. Within this topology‐guided framework, the hydrophobic pocket identified by blind docking spans TM1–TM3, the Walker A nucleotide‐binding motif and helix H1, i.e., functional regions implicated in MW formation, NTPase activity and RNA binding [22], thereby providing additional biological support for the relevance of this site. The recurring involvement of these regions defines an extended, hydrophobic, and functionally integrated binding site capable of accommodating rigid or partially flexible molecular scaffolds, and the ability of related analog pairs to fit the same pocket suggests a degree of structural adaptability that could be exploited for further optimization.

The presence of the Walker A NBM within this cavity is particularly relevant, as NS4B has been reported to possess NTPase activity that is essential for the assembly of the viral replication complex. Ligand binding at this site may interfere with ATP or GTP coordination, thereby impairing enzymatic or scaffolding functions. Engagement of helix H1—implicated in RNA binding and NS4B oligomerization—suggests additional potential to disrupt RNA recruitment or multimeric viral replication complex formation.

Even compounds with lower docking scores (e.g., **8**, **21**) are predicted to bind to the same cavity of NS4B, indicating that accessibility is not a limiting factor, but specific hydrophobic and conserved interactions with amino acid residues of the cavity determine the strength of binding between the compound and the NS4B protein. Further support for the functional importance of this pocket came from docking three known NS4B inhibitors—amphihevir, anguizole, and clemizole—into the same site. These agents displayed strong predicted affinities, with amphihevir and anguizole outperforming most compounds in this series.

Reported resistance‐associated mutations for these inhibitors map to structurally relevant regions (H94R, F98C/L, V105M in TM1 for amphihevir and anguizole, R214Q near helix H1 for clemizole) [22, 29], which lie within or adjacent to the extended TM1–TM3/H1 region encompassed by our predicted binding pocket. Although **30b** showed a slightly lower predicted affinity than some analogs, its potent in vitro antiviral activity and synergistic effect with peg‐IFN and DCV suggest that NS4B engagement could contribute to its biological mechanism. The similarity in the binding mode between **30b** and other compounds supports a common, functionally important NS4B‐targeting mechanism, positioning **30b** as a promising basis for further development. At the same time, these mechanistic insights arise from a topology‐guided homology model combined with docking analysis and therefore represent a structural hypothesis that we anticipate will be further tested and refined by future experimental and computational studies in membrane‐mimetic environments.

## 3 Conclusions

In the present study, we synthesized a series of novel imidazo[4,5‐*c*]pyridine compounds, based on the main structural features of the known HCV NS4B inhibitors Clemizole and Anguizole that seem to be essential for NS4B inhibition, and evaluated their efficacy against HCV. The synthesis of all the target derivatives was effected starting from 4‐amino‐2‐chloropyridine, which was converted through a series of reactions into the imidazo[4,5‐*c*]pyridine compounds, substituted at position −4 of the scaffold. Then, on selected derivatives, the alkyl substituents were introduced at position −1 or −3 of the imidazole ring.

The findings of this study suggest that a number of the newly synthesized compounds demonstrate efficacy against HCV genotype 1b propagation. The activity of the compounds studied was determined in Huh5.2 cells, which stably express the HCV‐Con1 (1b) subgenomic replicon. Compound **30b** was found to be the most potent against HCV replication, with EC_50_ value of 6.61 µM according to the luciferase reporter assay. These results were further confirmed by the quantification of viral RNA and NS5A protein levels, demonstrating a clear attenuation of HCV replication. Of great significance was the fact that compound **30b** exerted a synergistic effect with peg‐IFN, which renders this novel antiviral agent as a promising candidate in combination therapies.

In silico docking studies identified a conserved hydrophobic pocket in NS4B (TM1‐TM3, Walker A motif, H1 helix) as the common binding site for all compounds, with **30b** exhibiting a docking score of −6.6 kcal/mol through key hydrophobic and H–bond interactions, corroborating its anti‐HCV potency and validating the imidazo[4,5‐*c*]pyridine scaffold for NS4B‐targeted optimization.

Importantly, the intrinsically high mutation rate of HCV, driven by the error‐prone nature of its RNA‐dependent RNA polymerase, continues to facilitate the rapid emergence of resistance‐associated variants, even under highly effective DAA regimens. This evolutionary plasticity highlights the urgent need to expand the repertoire of direct‐acting therapeutics that can be rationally integrated into multidrug combinations to suppress resistance, enhance antiviral durability, and support global efforts toward the sustained elimination of chronic HCV infection. In this context, the pharmacological profile of compound **30b** positions it as a compelling lead candidate for further mechanistic elucidation of its mode of action and development within combination‐oriented antiviral frameworks.

## 4 Experimental Section

### 4.1 Chemistry

#### 4.1.1 General Information

Melting points were determined on a Büchi apparatus and are uncorrected. Flash chromatography was performed on Merck silica gel 60 (0.040–0.063 mm), while basic aluminum oxide (Thermo Scientific chemicals, Brockmann I, 50–200 µm, 90A) was used for the separation of each pair of the regioisomeric target derivatives **24a–30a/24b–30b**. Analytical thin layer chromatography (TLC) was carried out on precoated (0.25 mm) Merck silica gel F‐254 plates. ^1^H NMR and ^13^C NMR spectra were recorded on a Bruker Avance III 600 or a Bruker Avance DRX 400 instrument, in deuterated solvents, and they were referenced to TMS (δ scale). Mass spectra were recorded using a UPLC Triple TOF‐MS instrument (UPLC: Acquity of Waters, SCIEX Triple TOF‐MS 5600+).

#### 4.1.2 2‐Chloro‐N‐(2‐chloro‐3‐nitropyridin‐4‐yl)acetamide (5)

Chloroacetyl chloride (0.27 mL, 3.39 mmol) was added dropwise into a solution of **3** (0.4 g, 2.30 mmol) in anhydrous DMF (2 mL), at 0 °C and under argon, and the reaction mixture was then stirred at room temperature for 17 h. Upon completion of the reaction, the mixture was poured into crushed ice and was further diluted with water. The precipitate formed was filtered under vacuum, washed with water and air‐dried, to afford the derivative **5** (0.53 g, yield 92%) as a white solid. M.p. 104–105 °C (CH_2_Cl_2_). ^1^H NMR (400 MHz, DMSO‐*d*_6_) δ 4.41 (s, 2H, CH_2_), 8.00 (d, 1H, *J* = 5.7 Hz, H‐5), 8.55 (d, 1H, *J* = 5.7 Hz, H‐6), 10.94 (brs, 1H, D_2_O exch., NH). ^13^C NMR (100 MHz, DMSO‐*d*_6_) δ 43.15, 117.54, 136.81, 139.48, 141.95, 151.58, 166.33.

#### 4.1.3 N‐(3‐Amino‐2‐chloropyridin‐4‐yl)‐2‐chloroacetamide (6)

A solution of the nitroderivative **5** (0.52 g, 2.08 mmol) in absolute ethanol (50 mL) was hydrogenated in the presence of Raney nickel (80 mg), under a pressure of 45 psi at room temperature for 3 h. The solution was filtered through a Celite pad to remove the catalyst, and the filtrate was evaporated to dryness. The crude product was purified by column chromatography (silica gel), using a mixture of dichloromethane/methanol as the eluent (from 100/1 up to 85/15, v/v), to provide the pure aminoderivative **6** (0.2 g, yield 44%) as a gray solid. M.p. 141–142 °C (CHCl_3_). ^1^H NMR (600 MHz, DMSO‐*d*_6_) δ 4.35 (s, 2H, CH_2_), 5.40 (brs, 2H, D_2_O exch., NH_2_), 7.55 (d, 1H, *J* = 5.2 Hz, H‐5), 7.60 (d, 1H, *J* = 5.2 Hz, H‐6), 9.72 (brs, 1H, D_2_O exch., NH). ^13^C NMR (151 MHz, DMSO‐*d*_6_) δ 43.34, 117.46, 130.45, 133.26, 136.05, 136.42, 165.32.

#### 4.1.4 N‐(3‐Amino‐2‐chloropyridin‐4‐yl)‐2‐(4‐methylpiperazin‐1‐yl)acetamide (7)

Potassium iodide (0.17 g, 1.02 mmol) was added to a suspension of intermediate **6** (0.16 g, 0.73 mmol) in absolute ethanol (5 mL) under argon, followed by the dropwise addition of a solution of *N*‐methylpiperazine (0.49 mL, 4.42 mmol) in ethanol (0.5 mL). The resulting reaction mixture was then heated at 60 °C for 2 h. Upon completion of the reaction, the organic solvent was removed under reduced pressure, water was added into the flask, and the residue was extracted with ethyl acetate. The combined organic layers were dried over sodium sulfate and evaporated. The crude product was purified by column chromatography (silica gel), using a mixture of dichloromethane/methanol as the eluent (from 95/5 up to 75/25, v/v), to provide the pure derivative **7** (145 mg, yield 70%) as a white solid. M.p. 159–160 °C (CH_2_Cl_2_/*n*‐pentane). ^1^H NMR (600 MHz, CDCl_3_) δ 2.32 (s, 3H, piperazine‐CH_3_), 2.52 (brs, 4H, piperazine H), 2.69 (brs, 4H, piperazine H), 3.19 (s, 2H, CH_2_), 4.11 (brs, 2H, D_2_O exch., NH_2_), 7.54 (d, 1H, *J* = 5.2 Hz, H‐5), 7.82 (d, 1H, *J* = 5.2 Hz, H‐6), 9.43 (brs, 1H, D_2_O exch., NH). ^13^C NMR (151 MHz, CDCl_3_) δ 45.99, 53.48, 55.33, 61.69, 115.84, 131.19, 133.45, 140.00, 140.43, 169.19.

#### 4.1.5 4‐Chloro‐2‐((4‐methylpiperazin‐1‐yl)methyl)‐1*H*‐imidazo[4,5‐*c*]pyridine (8)

The aminoderivative **7** (180 mg, 0.64 mmol) was added to a flask containing polyphosphoric acid (7 g) at 60 °C, and this mixture was heated at 100 °C for 3 h. Then, upon cooling, the oily residue was poured into crushed ice, diluted with water, basified to pH = 10 with ammonium hydroxide solution, and extracted with ethyl acetate. The combined organic layers were dried over sodium sulfate and evaporated. The crude product was purified by column chromatography (silica gel), using a mixture of dichloromethane/methanol as the eluent (from 95/5 up to 85/15, v/v), to provide the pure target derivative **8** (60 mg, yield 36%) as a beige solid. M.p. 216–218 °C (EtOH). ^1^H NMR (400 MHz, DMSO‐*d*_6_) δ 2.19 (s, 3H, piperazine‐CH_3_), 2.40 (brs, 4H, piperazine H), 2.48 (brs, 4H, piperazine H, overlapping with DMSO‐*d*_6_), 3.77 (s, 2H, CH_2_), 7.51 (d, 1H, *J* = 5.4 Hz, H‐7), 8.07 (d, 1H, *J* = 5.4 Hz, H‐6), 13.01 (brs, 1H, D_2_O exch., NH). ^13^C NMR (101 MHz, DMSO‐*d*_6_) δ 45.50, 52.54, 54.37, 55.32, 107.70, 129.51, 136.91, 140.49, 141.25, 154.72. HRMS (ESI) m/z: calculated for C_12_H_17_ClN_5_ [M + H]^+^: 266.1167; found 266.1169.

#### 4.1.6 2‐Ethoxy‐3‐nitropyridin‐4‐amine (9)

Sodium ethoxide (0.68 g, 10 mmol) was added to a solution of the chloroderivative **3** (0.60 g, 3.46 mmol) in absolute ethanol (30 mL), at 0 °C and under argon, and then this reaction mixture was heated at 75 °C for 90 min. Upon completion of the reaction, the organic solvent was evaporated, cold water was carefully added into the flask, followed by addition of a 2N hydrochloric acid solution until the pH was 5. Then this mixture was extracted with ethyl acetate, the combined organic layers were dried over sodium sulfate and evaporated, to provide the pure derivative **9** (0.61 g, yield 96%) as a beige solid. M.p. 60–61 °C (EtOAc/*n*‐pentane). ^1^H NMR (400 MHz, CDCl_3_) δ 1.39 (t, 3H, *J* = 7.1 Hz, C*H*_3_CH_2_), 4.45 (q, 2H, *J* = 7.1 Hz, CH_3_C*H*_2_), 6.02 (brs, 2H, D_2_O exch., NH_2_), 6.29 (d, 1H, *J* = 5.9 Hz, H‐5), 7.74 (d, 1H, *J* = 5.9 Hz, H‐6). ^13^C NMR (100 MHz, CDCl_3_) δ 14.56, 63.45, 106.70, 120.69, 148.49, 150.66, 158.79.

#### 4.1.7 3‐Nitro‐2‐(2,2,2‐trifluoroethoxy)pyridin‐4‐amine (10)

Sodium hydride (60% dispersion in mineral oil, 346 mg, 8.64 mmol) was added to a solution of 2,2,2‐trifluoroethanol (0.63 mL, 8.64 mmol) in anhydrous DMF (5 mL), at 0 °C and under argon, and this mixture was stirred at room temperature for 30 min. Then, the reaction was cooled again to 0 °C, the chloroderivative **3** (0.5 g, 2.88 mmol) was added, and this mixture was heated at 70 °C for 2 h. Upon completion of the reaction, the mixture was poured into cold water and extracted with ethyl acetate; the combined organic layers were washed with brine, dried over sodium sulfate, and evaporated. The crude product was purified by column chromatography (silica gel), using a mixture of cyclohexane/ethyl acetate as the eluent (from 90/10 up to 20/80, v/v), to provide the pure derivative **10** (320 mg, yield 47%) as a yellow solid. M.p. 83–84 °C (CH_2_Cl_2_/*n*‐pentane). ^1^H NMR (600 MHz, CDCl_3_) δ 4.82 (q, 2H, *J* = 8.3 Hz, OCH_2_CF_3_), 6.12 (brs, 2H, D_2_O exch., NH_2_), 6.40 (d, 1H, *J* = 5.9 Hz, H‐5), 7.74 (d, 1H, *J* = 5.9 Hz, H‐6). ^13^C NMR (151 MHz, CDCl_3_) δ 62.51 (CH_2_CF_3_), 62.76 (CH_2_CF_3_), 63.00 (CH_2_CF_3_), 63.24 (CH_2_CF_3_), 108.57, 120.32, 120.62 (CH_2_ CF_3_), 122.46 (CH_2_ CF_3_), 124.30 (CH_2_ CF_3_), 126.14 (CH_2_ CF_3_), 147.94, 150.98, 156.65.

#### 4.1.8 3‐Nitro‐2‐phenylpyridin‐4‐amine (11)

The chloroderivative **3** (0.5 g, 2.88 mmol) was dissolved under argon in a mixture of toluene (56 mL) and ethanol (6 mL), followed by addition of phenylboronic acid (0.39 g, 3.20 mmol), tetrakis(triphenylphosphine)palladium (0) (0.23 g, 0.20 mmol) and a solution of potassium carbonate (1 g, 7.25 mmol) in 4 mL of water, and this mixture was refluxed for 16 h. Then, the organic solvents were removed under reduced pressure, and the residue was extracted with ethyl acetate. The combined organic layers were dried over sodium sulfate and evaporated. The crude product was purified by column chromatography (silica gel), using a mixture of cyclohexane/ethyl acetate as the eluent (from 70/30 up to 20/80, v/v), to provide the pure derivative **11** (410 mg, yield 66%) as a yellow solid. M.p. 205–206 °C (Et_2_O). ^1^H NMR (400 MHz, CDCl_3_) δ 5.67 (brs, 2H, D_2_O exch., NH_2_), 6.61 (d, 1H, *J* = 5.8 Hz, H‐5), 7.40–7.44 (m, 3H, phenyl H‐3′, H‐4′, H‐5′), 7.47–7.52 (m, 2H, phenyl H‐2′, H‐6′), 8.21 (d, 1H, *J* = 5.8 Hz, H‐6). ^13^C NMR (100 MHz, CDCl_3_) δ 110.90, 127.68, 128.77, 129.43, 132.66, 138.04, 147.76, 150.06, 156.11.

#### 4.1.9 2‐Chloro‐N‐(2‐ethoxy‐3‐nitropyridin‐4‐yl)acetamide (12)

This compound was synthesized by following an analogous procedure to that described for the preparation of the derivative **5**, upon reaction of compound **9** (0.51 g, 2.79 mmol) with chloroacetyl chloride (0.33 mL, 4.19 mmol). The pure compound **12** (0.63 g, yield 87%) was obtained as a white solid, m.p. 127–128  °C (CH_2_Cl_2_/Et_2_O). ^1^H NMR (600 MHz, CDCl_3_) δ 1.42 (t, 3H, *J* = 7.1 Hz, C*H*_3_CH_2_), 4.21 (s, 2H, CH_2_Cl), 4.52 (q, 2H, *J* = 7.1 Hz, CH_3_C*H*_2_), 8.07 (d, 1H, *J* = 5.9 Hz, H‐5), 8.20 (d, 1H, *J* = 5.9 Hz, H‐6), 10.28 (brs, 1H, D_2_O exch., NH). ^13^C NMR (151 MHz, CDCl_3_) δ 14.46, 43.05, 64.13, 108.51, 124.33, 140.28, 150.86, 157.34, 165.29.

#### 4.1.10 2‐Chloro‐N‐(3‐nitro‐2‐(2,2,2‐trifluoroethoxy)pyridin‐4‐yl)acetamide (13)

This compound was synthesized by following an analogous procedure to that described for the preparation of the derivative **5**, upon reaction of compound **10** (2.6 g, 10.97 mmol) with chloroacetyl chloride (1.31 mL, 16.46 mmol). The pure compound **13** (3.12 g, yield 91%) was obtained as a beige solid, m.p. 93–94  °C (CH_2_Cl_2_/*n*‐pentane). ^1^H NMR (400 MHz, CDCl_3_) δ 4.23 (s, 2H, CH_2_Cl), 4.90 (q, 2H, *J* = 8.2 Hz, OCH_2_CF_3_), 8.23 (d, 1H, *J* = 5.9 Hz, H‐5), 8.27 (d, 1H, *J* = 5.9 Hz, H‐6), 10.42 (brs, 1H, D_2_O exch., NH). ^13^C NMR (100 MHz, CDCl_3_) δ 43.05, 62.67 (CH_2_CF_3_), 63.04 (CH_2_CF_3_), 63.41 (CH_2_CF_3_), 63.78 (CH_2_CF_3_), 110.44, 119.14 (CH_2_ CF_3_), 121.69 (CH_2_ CF_3_), 124.04, 124.46 (CH_2_ CF_3_), 127.22 (CH_2_ CF_3_), 141.06, 150.45, 155.21, 165.42.

#### 4.1.11 2‐Chloro‐N‐(3‐nitro‐2‐phenylpyridin‐4‐yl)acetamide (14)

This compound was synthesized by following an analogous procedure to that described for the preparation of the derivative **5**, upon reaction of compound **11** (0.86 g, 4.0 mmol) with chloroacetyl chloride (0.48 mL, 6.0 mmol). An additional amount of chloroacetyl chloride (0.1 mL, 1.25 mmol) was added to the reaction mixture after 17 h, and the reaction mixture was then stirred for an additional 3 h at room temperature in order to complete. Then, the mixture was poured into cold water and extracted with ethyl acetate; the combined organic layers were washed with brine, dried over sodium sulfate, and evaporated. The crude product was purified by column chromatography (silica gel), using a mixture of *n*‐hexane/ethyl acetate as the eluent (from 80/20 up to 20/80, v/v), to provide the pure derivative **14** (0.84 g, yield 72%) as a white solid. M.p. 112–113 °C (CH_2_Cl_2_/*n*‐pentane). ^1^H NMR (400 MHz, DMSO‐*d*_6_) δ 4.41 (s, 2H, CH_2_Cl), 7.45–7.52 (m, 5H, phenyl H), 7.95 (d, 1H, *J* = 5.6 Hz, H‐5), 8.77 (d, 1H, *J* = 5.6 Hz, H‐6), 10.65 (brs, 1H, D_2_O exch., NH). ^13^C NMR (100 MHz, DMSO‐*d*_6_) δ 43.10, 117.15, 127.58, 128.71, 129.65, 136.18, 137.55, 138.54, 151.62, 152.08, 166.22.

#### 4.1.12 N‐(2‐Ethoxy‐3‐nitropyridin‐4‐yl)‐2‐(4‐methylpiperazin‐1‐yl)acetamide (15)

This compound was synthesized by following an analogous procedure to that described for the preparation of the derivative **7**, starting from compound **12** (1.19 g, 4.59 mmol). The crude product was purified by column chromatography (silica gel), using a mixture of dichloromethane/methanol as the eluent (from 96/4 up to 75/25, v/v), to provide the pure derivative **15** (0.97 g, yield 66%) as an orange‐colored solid. M.p. 58–59 °C (Et_2_O). ^1^H NMR (600 MHz, CDCl_3_) δ 1.39 (t, 3H, *J* = 7.1 Hz, C*H*_3_CH_2_), 2.36 (s, 3H, piperazine‐CH_3_), 2.53–2.70 (m, 8H, piperazine H), 3.16 (s, 2H, CH_2_‐piperazine), 4.48 (q, 2H, *J* = 7.1 Hz, CH_3_C*H*_2_), 8.11–8.15 (m, 2H, H‐5, H‐6), 10.90 (brs, 1H, D_2_O exch., NH). ^13^C NMR (151 MHz, CDCl_3_) δ 14.48, 45.85, 53.33, 54.85, 61.95, 63.76, 108.35, 124.34, 140.53, 150.26, 156.96, 170.16.

#### 4.1.13 2‐(4‐Methylpiperazin‐1‐yl)‐N‐(3‐nitro‐2‐(2,2,2‐trifluoroethoxy)pyridin‐4‐yl)acetamide (16)

This compound was synthesized by following an analogous procedure to that described for the preparation of the derivative **7**, starting from compound **13** (0.36 g, 1.15 mmol). The crude product was purified by column chromatography (silica gel), using a mixture of dichloromethane/methanol as the eluent (from 99/1 up to 75/25, v/v), to provide the pure derivative **16** (0.32 g, yield 74%) as an orange‐colored oil. ^1^H NMR (400 MHz, CDCl_3_) δ 2.39 (s, 3H, piperazine‐CH_3_), 2.57–2.74 (m, 8H, piperazine H), 3.19 (s, 2H, CH_2_‐piperazine), 4.87 (q, 2H, *J* = 8.3 Hz, OCH_2_CF_3_), 8.14 (d, 1H, *J* = 5.9 Hz, H‐5), 8.33 (d, 1H, *J* = 5.9 Hz, H‐6), 11.08 (brs, 1H, D_2_O exch., NH). ^13^C NMR (100 MHz, CDCl_3_) δ 45.77, 53.23, 54.80, 61.89, 62.45 (CH_2_CF_3_), 62.81 (CH_2_CF_3_), 63.18 (CH_2_CF_3_), 63.55 (CH_2_CF_3_), 110.22, 118.99 (CH_2_ CF_3_), 121.75 (CH_2_ CF_3_), 123.94, 124.50 (CH_2_ CF_3_), 127.26 (CH_2_ CF_3_), 141.33, 149.88, 154.86, 170.26.

#### 4.1.14 2‐(4‐Methylpiperazin‐1‐yl)‐N‐(3‐nitro‐2‐phenylpyridin‐4‐yl)acetamide (17)

This compound was synthesized by following an analogous procedure to that described for the preparation of the derivative **7**, starting from compound **14** (0.24 g, 0.82 mmol). The crude product was purified by column chromatography (silica gel), using a mixture of dichloromethane/methanol as the eluent (from 99/1 up to 75/25, v/v), to provide the pure derivative **17** (0.32 g, yield 74%) as a pale yellow solid. M.p. 134–135 °C (EtOAc/*n*‐pentane). ^1^H NMR (400 MHz, CDCl_3_) δ 2.33 (s, 3H, piperazine‐CH_3_), 2.57 (brs, 4H, piperazine H), 2.65 (brs, 4H, piperazine H), 3.17 (s, 2H, CH_2_‐piperazine), 7.40–7.46 (m, 3H, phenyl H‐3′, H‐4′, H‐5′), 7.52–7.57 (m, 2H, phenyl H‐2′, H‐6′), 8.51 (d, 1H, *J* = 5.7 Hz, H‐5), 8.63 (d, 1H, *J* = 5.7 Hz, H‐6), 10.73 (brs, 1H, D_2_O exch., NH). ^13^C NMR (100 MHz, CDCl_3_) δ 45.84, 53.32, 54.89, 61.76, 113.35, 127.82, 128.93, 129.91, 136.37, 136.54, 138.14, 151.90, 153.68, 170.08.

#### 4.1.15 N‐(3‐Amino‐2‐ethoxypyridin‐4‐yl)‐2‐(4‐methylpiperazin‐1‐yl)acetamide (18)

A solution of the nitroderivative **15** (0.94 g, 2.91 mmol) in absolute ethanol (75 mL) was hydrogenated in the presence of 10% Pd/C (120 mg), under a pressure of 54 psi at room temperature for 5 h. Then, the solution was filtered through a Celite pad to remove the catalyst, and the filtrate was evaporated to dryness. The crude product was purified by column chromatography (silica gel), using a mixture of dichloromethane/methanol as the eluent (from 95/5 up to 75/25, v/v), to provide the pure aminoderivative **18** (0.83 g, yield 97%) as an oil. ^1^H NMR (600 MHz, CDCl_3_) δ 1.39 (t, 3H, *J* = 7.1 Hz, C*H*_3_CH_2_), 2.33 (s, 3H, piperazine‐CH_3_), 2.54 (brs, 4H, piperazine H), 2.69 (brs, 4H, piperazine H), 3.17 (s, 2H, CH_2_‐piperazine), 3.92 (brs, 2H, D_2_O exch., NH_2_), 4.39 (q, 2H, *J* = 7.1 Hz, CH_3_C*H*_2_), 6.98 (d, 1H, *J* = 5.5 Hz, H‐5), 7.55 (d, 1H, *J* = 5.5 Hz, H‐6), 9.21 (brs, 1H, D_2_O exch., NH). ^13^C NMR (151 MHz, CDCl_3_) δ 14.89, 45.88, 53.33, 55.23, 61.65, 62.18, 111.27, 122.54, 130.74, 136.08, 154.98, 168.66.

#### 4.1.16 N‐(3‐Amino‐2‐(2,2,2‐trifluoroethoxy)pyridin‐4‐yl)‐2‐(4‐methylpiperazin‐1‐yl)acetamide (19)

A solution of the nitroderivative **16** (2.48 g, 6.58 mmol) in absolute ethanol (100 mL) was hydrogenated in the presence of 10% Pd/C (150 mg), under a pressure of 54 psi at room temperature for 6 h. Then, the solution was filtered through a Celite pad to remove the catalyst, and the filtrate was evaporated to dryness. The crude product was purified by column chromatography (silica gel), using a mixture of dichloromethane/methanol as the eluent (from 98/2 up to 75/25, v/v), to provide the pure aminoderivative **19** (2.19 g, yield 96%) as an oil. ^1^H NMR (600 MHz, CDCl_3_) δ 2.29 (s, 3H, piperazine‐CH_3_), 2.49 (brs, 4H, piperazine H), 2.66 (brs, 4H, piperazine H), 3.15 (s, 2H, CH_2_‐piperazine), 3.87 (brs, 2H, D_2_O exch., NH_2_), 4.76 (q, 2H, *J* = 8.6 Hz, OCH_2_CF_3_), 7.17 (d, 1H, *J* = 5.4 Hz, H‐5), 7.53 (d, 1H, *J* = 5.5 Hz, H‐6), 9.32 (brs, 1H, D_2_O exch., NH). ^13^C NMR (100 MHz, CDCl_3_) δ 45.56, 52.90, 55.05, 61.55, 62.06 (CH_2_CF_3_), 62.41 (CH_2_CF_3_), 62.77 (CH_2_CF_3_), 63.13 (CH_2_CF_3_), 112.74, 119.69 (CH_2_ CF_3_), 122.10, 122.45 (CH_2_ CF_3_), 125.20 (CH_2_ CF_3_), 127.96 (CH_2_ CF_3_), 132.22, 135.93, 152.56, 168.72.

#### 4.1.17 N‐(3‐Amino‐2‐phenylpyridin‐4‐yl)‐2‐(4‐methylpiperazin‐1‐yl)acetamide (20)

This compound was synthesized by following an analogous procedure to that described for the preparation of the derivative **18**, starting from compound **17** (0.65 g, 1.83 mmol). The reaction time was 3 h. The crude product was purified by column chromatography (silica gel), using a mixture of dichloromethane/methanol as the eluent (from 96/4 up to 75/25, v/v), to provide the pure aminoderivative **20** (0.55 g, yield 92%) as an orange‐colored solid. M.p. 164–166 °C (CH_2_Cl_2_/Et_2_O). ^1^H NMR (400 MHz, CDCl_3_) δ 2.31 (s, 3H, piperazine‐CH_3_), 2.53 (brs, 4H, piperazine H), 2.70 (brs, 4H, piperazine H), 3.20 (s, 2H, CH_2_‐piperazine), 3.81 (brs, 2H, D_2_O exch., NH_2_), 7.40 (t, 1H, *J* = 7.3 Hz, phenyl H‐4′), 7.47 (t, 2H, *J* = 7.4 Hz, phenyl H‐3′, H‐5′), 7.57 (d, 2H, *J* = 7.3 Hz, phenyl H‐2′, H‐6′), 7.64 (d, 1H, *J* = 5.4 Hz, H‐5), 8.14 (d, 1H, *J* = 5.4 Hz, H‐6), 9.43 (brs, 1H, D_2_O exch., NH). ^13^C NMR (100 MHz, CDCl_3_) δ 45.90, 53.40, 55.29, 61.80, 115.32, 128.64, 128.76, 129.06, 131.41, 133.10, 138.36, 141.36, 148.70, 169.21.

#### 4.1.18 4‐Ethoxy‐2‐((4‐methylpiperazin‐1‐yl)methyl)‐1*H*‐imidazo[4,5‐*c*]pyridine (21)

The aminoderivative **18** (330 mg, 1.13 mmol) was dissolved in glacial acetic acid (4 mL), and this mixture was heated at 100 °C under argon for 5 h. Upon completion of the reaction, the mixture was diluted with water, an aqueous solution of sodium hydroxide (2N) was added until the pH was 10, followed by extraction with ethyl acetate. The combined organic layers were dried over sodium sulfate and evaporated. The crude product was purified by column chromatography (silica gel), using a mixture of dichloromethane/methanol as the eluent (from 96/4 up to 75/25, v/v), to provide the pure target compound **21** (195 mg, yield 63%) as an oil. ^1^H NMR (400 MHz, DMSO‐*d*_6_) δ 1.37 (t, 3H, *J* = 7.0 Hz, C*H*_3_CH_2_), 2.14 (s, 3H, piperazine‐CH_3_), 2.33 (brs, 4H, piperazine H), 2.44 (brs, 4H, piperazine H), 3.68 (s, 2H, CH_2_‐piperazine), 4.44 (q, 2H, *J* = 7.1 Hz, CH_3_C*H*_2_), 7.06 (brs, 1H, H‐7), 7.77 (d, 1H, *J* = 5.7 Hz, H‐6), 12.63 (brs, 1H, D_2_O exch., NH). ^13^C NMR (100 MHz, DMSO‐*d*_6_) δ 14.70, 45.68, 52.66, 54.51, 55.50, 60.80, 102.22, 127.42, 138.02, 140.94, 151.10, 154.81. HRMS (ESI) m/z: calculated for C_14_H_22_N_5_O [M + H]^+^: 276.1819; found 276.1821.

#### 4.1.19 2‐((4‐Methylpiperazin‐1‐yl)methyl)‐4‐(2,2,2‐trifluoroethoxy)‐1*H*‐imidazo[4,5‐*c*]pyridine (22)

This compound was synthesized by following an analogous procedure to that described for the preparation of the derivative **21**, starting from compound **19** (2 g, 5.76 mmol). The crude product was purified by column chromatography (silica gel), using a mixture of dichloromethane/methanol as the eluent (from 96/4 up to 75/25, v/v), to provide the pure target compound **22** (1.76 g, yield 93%) as a white solid. M.p. 110–111 °C (CH_2_Cl_2_/Et_2_O). ^1^H NMR (600 MHz, DMSO‐*d*_6_) δ 2.15 (s, 3H, piperazine‐CH_3_), 2.35 (brs, 4H, piperazine H), 2.45 (brs, 4H, piperazine H), 3.72 (s, 2H, CH_2_‐piperazine), 5.12 (q, 2H, *J* = 9.2 Hz, OCH_2_CF_3_), 7.22 (d, 1H, *J* = 5.6 Hz, H‐7), 7.82 (d, 1H, *J* = 5.7 Hz, H‐6), 12.82 (brs, 1H, D_2_O exch., NH). ^13^C NMR (151 MHz, DMSO‐*d*_6_) δ 45.63, 52.65, 54.48, 55.41, 60.45 (CH_2_CF_3_), 60.79 (CH_2_CF_3_), 61.13 (CH_2_CF_3_), 61.48 (CH_2_CF_3_), 103.96, 120.12 (CH_2_ CF_3_), 122.88 (CH_2_ CF_3_), 125.64 (CH_2_ CF_3_), 127.08, 128.40 (CH_2_ CF_3_), 137.46, 141.80, 152.40. HRMS (ESI) m/z: calculated for C_14_H_19_F_3_N_5_O [M + H]^+^: 330.1537; found 330.1549.

#### 4.1.20 2‐((4‐Methylpiperazin‐1‐yl)methyl)‐4‐phenyl‐1*H*‐imidazo[4,5‐*c*]pyridine (23)

This compound was synthesized by following an analogous procedure to that described for the preparation of the derivative **21**, starting from compound **20** (400 mg, 1.23 mmol). The crude product was purified by column chromatography (silica gel), using a mixture of dichloromethane/methanol as the eluent (from 95/5 up to 85/15, v/v), to provide the pure target compound **23** (320 mg, yield 85%) as a white solid. M.p. 211–212 °C (CHCl_3_/Et_2_O). ^1^H NMR (600 MHz, DMSO‐*d*_6_) δ 2.17 (s, 3H, piperazine‐CH_3_), 2.38 (brs, 4H, piperazine H), 2.49 (brs, 4H, piperazine H, overlapping with DMSO‐*d*_6_), 3.82 (s, 2H, CH_2_‐piperazine), 7.40–7.46 (m, 2H, H‐7, phenyl H‐4′), 7.52 (t, 2H, *J* = 7.4 Hz, phenyl H‐3′, H‐5′), 8.37 (d, 1H, J = 5.4 Hz, H‐6), 8.72 (brs, 2H, phenyl H‐2′, H‐6′), 12.81 (brs, 1H, D_2_O exch., NH). ^13^C NMR (100 MHz, DMSO‐*d*_6_) δ 45.67, 52.74, 54.49, 55.63, 106.25, 128.11, 128.65, 128.77, 137.72, 138.03, 140.53, 140.81, 146.45, 153.52. HRMS (ESI) m/z: calculated for C_18_H_22_N_5_ [M + H]^+^: 308.1870; found 308.1873.

#### 4.1.21 4‐Ethoxy‐1‐methyl‐2‐((4‐methylpiperazin‐1‐yl)methyl)‐1*H*‐imidazo[4,5‐*c*]pyridine (24a) and 4‐ethoxy‐3‐methyl‐2‐((4‐methylpiperazin‐1‐yl)methyl)‐3*H*‐imidazo[4,5‐*c*]pyridine (24b)

Potassium carbonate (211 mg, 1.53 mmol) was added to a solution of compound **21** (420 mg, 1.53 mmol) in DMSO (4 mL), and this mixture was stirred at room temperature for 20 min. Then, a solution of iodomethane (95 µL, 1.53 mmol) in DMSO (1 mL) was added dropwise, over a period of 10 min, and the reaction mixture was stirred at room temperature for 5 h. After that time, additional amounts of potassium carbonate (106 mg, 0.77 mmol) and iodomethane (48 µL, 0.77 mmol, in 1 mL of DMSO) were added, and the reaction was stirred for 16 h. Upon completion of the reaction, the mixture was diluted with water and extracted with ethyl acetate. The combined organic layers were washed with brine, dried over sodium sulfate, and evaporated. The residue was subjected to column chromatography (silica gel), using a mixture of dichloromethane/methanol as the eluent (from 98/2 up to 75/25, v/v), to provide a mixture of the two regioisomers **24a** and **24b**. The latter was separated by a second column chromatography, using basic aluminum oxide as the stationary phase and a mixture of chloroform/methanol as the eluent (from 99.8/0.2 up to 99.5/0.5, v/v), to yield the pure target compounds **24a** and **24b**.

**[24a]**: 70 mg, yield 16%. Beige solid, m.p. 204–205 °C (CH_2_Cl_2_/Et_2_O). ^1^H NMR (400 MHz, CDCl_3_) δ 1.49 (t, 3H, *J* = 7.1 Hz, C*H*_3_CH_2_), 2.30 (s, 3H, piperazine‐CH_3_), 2.47 (brs, 4H, piperazine H), 2.58 (brs, 4H, piperazine H), 3.82 (s, 2H, CH_2_‐piperazine), 3.83 (s, 3H, imidazole‐CH_3_), 4.57 (q, 2H, *J* = 7.1 Hz, CH_3_C*H*_2_), 6.89 (d, 1H, *J* = 5.8 Hz, H‐7), 7.90 (d, 1H, *J* = 5.8 Hz, H‐6). ^13^C NMR (100 MHz, CDCl_3_) δ 14.87, 30.83, 45.64, 52.62, 54.88, 55.15, 62.00, 100.02, 127.07, 139.20, 142.79, 150.57, 156.05. HRMS (ESI) m/z: calculated for C_15_H_24_N_5_O [M + H]^+^: 290.1976; found 290.1988.

**[24b]**: 140 mg, yield 32%. Beige solid, m.p. 189–190 °C (CH_2_Cl_2_/Et_2_O). ^1^H NMR (400 MHz, CDCl_3_) δ 1.46 (t, 3H, *J* = 7.1 Hz, C*H*_3_CH_2_), 2.30 (s, 3H, piperazine‐CH_3_), 2.49 (brs, 4H, piperazine H), 2.58 (brs, 4H, piperazine H), 3.77 (s, 2H, CH_2_‐piperazine), 4.08 (s, 3H, imidazole‐CH_3_), 4.52 (q, 2H, *J* = 7.1 Hz, CH_3_C*H*_2_), 7.19 (d, 1H, *J* = 5.8 Hz, H‐7), 7.82 (d, 1H, *J* = 5.8 Hz, H‐6). ^13^C NMR (100 MHz, CDCl_3_) δ 14.82, 32.77, 45.75, 52.79, 54.91, 55.13, 61.81, 109.42, 121.44, 137.94, 149.18, 151.75, 152.84. HRMS (ESI) m/z: calculated for C_15_H_24_N_5_O [M + H]^+^: 290.1976; found 290.1970.

#### 4.1.22 4‐Ethoxy‐1‐isopropyl‐2‐((4‐methylpiperazin‐1‐yl)methyl)‐1*H*‐imidazo[4,5‐*c*]pyridine (25a) and 4‐ethoxy‐3‐isopropyl‐2‐((4‐methylpiperazin‐1‐yl)methyl)‐3*H*‐imidazo[4,5‐*c*]pyridine (25b)

Potassium carbonate (477 mg, 3.45 mmol) was added to a solution of compound **21** (315 mg, 1.15 mmol) in DMSO (4 mL), and this mixture was stirred at room temperature for 20 min. Then, 2‐bromopropane (0.32 mL, 3.45 mmol) was added dropwise, and the reaction mixture was stirred at room temperature for 5 h. After that time, additional amounts of potassium carbonate (159 mg, 1.15 mmol) and 2‐bromopropane (0.11 mL, 1.15 mmol) were added, and the reaction was stirred for 16 h. Upon completion of the reaction, the mixture was diluted with water and extracted with ethyl acetate. The combined organic layers were washed with brine, dried over sodium sulfate, and evaporated. The residue was subjected to column chromatography (silica gel), using a mixture of dichloromethane/methanol as the eluent (from 95/5 up to 85/15, v/v), to provide a mixture of the two regioisomers **25a** and **25b**. The latter was separated by a second column chromatography, using basic aluminum oxide as the stationary phase and a mixture of chloroform/methanol as the eluent (from 100/0 up to 99.5/0.5, v/v), to yield the pure target compounds **25a** and **25b**.

**[25a]**: 110 mg, yield 30%, amorphous solid. ^1^H NMR (400 MHz, CDCl_3_) δ 1.48 (t, 3H, *J* = 7.1 Hz, C*H*_3_CH_2_), 1.58 (d, 6H, *J* = 7.0 Hz, isopropyl‐CH_3_), 2.30 (s, 3H, piperazine‐CH_3_), 2.45 (brs, 4H, piperazine H), 2.55 (brs, 4H, piperazine H), 3.82 (s, 2H, CH_2_‐piperazine), 4.56 (q, 2H, *J* = 7.1 Hz, CH_3_C*H*_2_), 5.00 (hept, 1H, *J* = 7.0 Hz, isopropyl‐CH), 7.04 (d, 1H, *J* = 5.9 Hz, H‐7), 7.84 (d, 1H, *J* = 5.9 Hz, H‐6). ^13^C NMR (100 MHz, CDCl_3_) δ 14.92, 21.54, 45.81, 48.61, 52.74, 55.10, 55.69, 61.91, 102.53, 128.05, 138.52, 140.31, 149.91, 156.47. HRMS (ESI) m/z: calculated for C_17_H_28_N_5_O [M + H]^+^: 318.2289; found 318.2278.

**[25b]**: 80 mg, yield 22%, amorphous solid. ^1^H NMR (400 MHz, CDCl_3_) δ 1.51 (t, 3H, *J* = 7.1 Hz, C*H*_3_CH_2_), 1.63 (d, 6H, *J* = 6.9 Hz, isopropyl‐CH_3_), 2.34 (s, 3H, piperazine‐CH_3_), 2.45–2.61 (m, 8H, piperazine H), 3.82 (s, 2H, CH_2_‐piperazine), 4.58 (q, 2H, *J* = 7.1 Hz, CH_3_C*H*_2_), 5.06 (brs, 1H, isopropyl‐CH), 7.23 (d, 1H, *J* = 5.7 Hz, H‐7), 7.87 (d, 1H, *J* = 5.7 Hz, H‐6). ^13^C NMR (100 MHz, CDCl_3_) δ 14.91, 22.43, 45.73, 49.41, 52.64, 55.02, 56.24, 62.07, 109.40, 120.14, 138.02, 150.38, 150.64, 152.36. HRMS (ESI) m/z: calculated for C_17_H_28_N_5_O [M + H]^+^: 318.2289; found 318.2277.

#### 4.1.23 1‐Cyclopentyl‐4‐ethoxy‐2‐((4‐methylpiperazin‐1‐yl)methyl)‐1*H*‐imidazo[4,5‐*c*]pyridine (26a) and 3‐cyclopentyl‐4‐ethoxy‐2‐((4‐methylpiperazin‐1‐yl)methyl)‐3*H*‐imidazo[4,5‐*c*]pyridine (26b)

These target compounds were synthesized by following an analogous procedure to that described for the preparation of the derivatives **25a** and **25b**, upon reaction of compound **21** (310 mg, 1.13 mmol) with bromocyclopentane. After the work‐up of the reaction, the residue was subjected to column chromatography (silica gel), using a mixture of dichloromethane/methanol as the eluent (from 97/3 up to 80/20, v/v), to provide a mixture of the two regioisomers **26a** and **26b**. The latter was separated by a second column chromatography, using basic aluminum oxide as the stationary phase and a mixture of dichloromethane/methanol as the eluent (from 100/0 up to 99/1, v/v), to yield the pure target compounds **26a** and **26b**.

**[26a]**: 150 mg, yield 39%. White solid, m.p. 104–105 °C (CH_2_Cl_2_/Et_2_O). ^1^H NMR (400 MHz, CDCl_3_) δ 1.47 (t, 3H, *J* = 7.1 Hz, C*H*_3_CH_2_), 1.72–1.83 (m, 2H, cyclopentyl H), 1.95–2.04 (m, 2H, cyclopentyl H), 2.06–2.17 (m, 4H, cyclopentyl H), 2.29 (s, 3H, piperazine‐CH_3_), 2.33–2.56 (m, 8H, piperazine H), 3.83 (s, 2H, CH_2_‐piperazine), 4.56 (q, 2H, *J* = 7.1 Hz, CH_3_C*H*_2_), 5.12 (p, 1H, *J* = 8.8 Hz, cyclopentyl H‐1′), 6.95 (d, 1H, *J* = 5.9 Hz, H‐7), 7.83 (d, 1H, *J* = 5.9 Hz, H‐6). ^13^C NMR (100 MHz, CDCl_3_) δ 14.91, 25.35, 30.76, 45.85, 52.73, 55.21, 55.73, 57.14, 61.91, 102.17, 128.05, 138.46, 140.02, 150.69, 156.52. HRMS (ESI) m/z: calculated for C_19_H_30_N_5_O [M + H]^+^: 344.2445; found 344.2449.

**[26b]**: 70 mg, yield 18%, amorphous solid. ^1^H NMR (400 MHz, CDCl_3_) δ 1.47 (t, 3H, *J* = 7.1 Hz, C*H*_3_CH_2_), 1.68–1.76 (m, 2H, cyclopentyl H), 1.97–2.08 (m, 4H, cyclopentyl H), 2.25–2.35 (m, 5H, piperazine‐CH_3_, cyclopentyl H), 2.42–2.62 (m, 8H, piperazine H), 3.82 (s, 2H, CH_2_‐piperazine), 4.56 (q, 2H, *J* = 7.1 Hz, CH_3_C*H*_2_), 5.07 (p, 1H, *J* = 8.8 Hz, cyclopentyl H‐1′), 7.22 (d, 1H, *J* = 5.7 Hz, H‐7), 7.86 (d, 1H, *J* = 5.7 Hz, H‐6). ^13^C NMR (100 MHz, CDCl_3_) δ 15.02, 24.23, 32.19, 45.69, 52.55, 55.07, 56.14, 58.46, 62.00, 109.43, 120.14, 138.03, 150.58, 150.71, 153.09. HRMS (ESI) m/z: calculated for C_19_H_30_N_5_O [M + H]^+^: 344.2445; found 344.2469.

#### 4.1.24 1‐Benzyl‐4‐ethoxy‐2‐((4‐methylpiperazin‐1‐yl)methyl)‐1*H*‐imidazo[4,5‐*c*]pyridine (27a) and 3‐benzyl‐4‐ethoxy‐2‐((4‐methylpiperazin‐1‐yl)methyl)‐3*H*‐imidazo[4,5‐*c*]pyridine (27b)

These target compounds were synthesized by following an analogous procedure to that described for the preparation of the derivatives **24a** and **24b**, upon reaction of compound **21** (190 mg, 0.69 mmol) with benzyl bromide. After the work‐up of the reaction, the residue was subjected to column chromatography (silica gel), using a mixture of dichloromethane/methanol as the eluent (from 100/0 up to 75/25, v/v), to provide a mixture of the two regioisomers **27a** and **27b**. The latter was separated by a second column chromatography, using basic aluminum oxide as the stationary phase and a mixture of chloroform/methanol as the eluent (from 100/0 up to 99.5/0.5, v/v), to yield the pure target compounds **27a** and **27b**.

**[27a]**: 30 mg, yield 12%, amorphous solid. ^1^H NMR (400 MHz, CDCl_3_) δ 1.52 (t, 3H, *J* = 7.1 Hz, C*H*_3_CH_2_), 2.43 (s, 3H, piperazine‐CH_3_), 2.50–2.75 (m, 8H, piperazine H), 3.81 (s, 2H, CH_2_‐piperazine), 4.61 (q, 2H, *J* = 7.1 Hz, CH_3_C*H*_2_), 5.48 (s, 2H, benzyl‐CH_2_), 6.83 (d, 1H, *J* = 5.8 Hz, H‐7), 6.99–7.03 (m, 2H, benzyl H), 7.28–7.35 (m, 3H, benzyl H), 7.90 (d, 1H, *J* = 5.8 Hz, H‐6). ^13^C NMR (100 MHz, CDCl_3_) δ 14.91, 43.60, 48.02, 49.57, 53.28, 54.17, 62.29, 100.37, 126.00, 127.21, 128.15, 129.24, 136.40, 140.10, 142.83, 149.48, 156.32. HRMS (ESI) m/z: calculated for C_21_H_28_N_5_O [M + H]^+^: 366.2289; found 366.2276.

**[27b]**: 25 mg, yield 10%, amorphous solid. ^1^H NMR (400 MHz, CDCl_3_) δ 1.28 (t, 3H, *J* = 7.1 Hz, C*H*_3_CH_2_), 2.45 (s, 3H, piperazine‐CH_3_), 2.55–2.75 (m, 8H, piperazine H), 3.74 (s, 2H, CH_2_‐piperazine), 4.47 (q, 2H, *J* = 7.1 Hz, CH_3_C*H*_2_), 5.78 (s, 2H, benzyl‐CH_2_), 7.00–7.05 (m, 2H, benzyl H), 7.27–7.34 (m, 4H, 3 x benzyl H, H‐7), 7.89 (d, 1H, *J* = 5.8 Hz, H‐6). ^13^C NMR (151 MHz, CDCl_3_) δ 14.65, 43.78, 49.21, 49.90, 53.52, 54.21, 61.99, 109.65, 121.26, 126.08, 127.78, 128.98, 137.75, 138.58, 149.39, 151.55, 152.03. HRMS (ESI) m/z: calculated for C_21_H_28_N_5_O [M + H]^+^: 366.2289; found 366.2274.

#### 4.1.25 1‐Methyl‐2‐((4‐methylpiperazin‐1‐yl)methyl)‐4‐(2,2,2‐trifluoroethoxy)‐1*H*‐imidazo[4,5‐*c*]pyridine (28a) and 3‐methyl‐2‐((4‐methylpiperazin‐1‐yl)methyl)‐4‐(2,2,2‐trifluoroethoxy)‐3*H*‐imidazo[4,5‐*c*]pyridine (28b)

These target compounds were synthesized by following an analogous procedure to that described for the preparation of the derivatives **24a** and **24b**, upon reaction of compound **22** (120 mg, 0.36 mmol) with iodomethane. After the work‐up of the reaction, the residue was subjected to column chromatography (silica gel), using a mixture of dichloromethane/methanol as the eluent (from 100/0 up to 85/15, v/v), to provide a mixture of the two regio isomers **28a** and **28b**. The latter was separated by a second column chromatography, using basic aluminum oxide as the stationary phase and a mixture of dichloromethane/ethyl acetate as the eluent (from 100/0 up to 0/100, v/v), to yield the pure target compounds **28a** and **28b**.

**[28a]**: 35 mg, yield 28%. White solid, m.p. 149–150 °C (CH_2_Cl_2_/*n*‐pentane). ^1^H NMR (400 MHz, methanol‐*d*_4_) δ 2.26 (s, 3H, piperazine‐CH_3_), 2.35–2.66 (m, 8H, piperazine H), 3.85 (s, 2H, CH_2_‐piperazine), 3.91 (s, 3H, imidazole‐CH_3_), 5.05 (q, 2H, *J* = 8.8 Hz, OCH_2_CF_3_), 7.25 (d, 1H, *J* = 5.8 Hz, H‐7), 7.92 (d, 1H, *J* = 5.8 Hz, H‐6). ^13^C NMR (100 MHz, methanol‐*d*_4_) δ 31.53, 46.10, 53.80, 55.29, 56.01, 62.45 (CH_2_CF_3_), 62.81 (CH_2_CF_3_), 63.16 (CH_2_CF_3_), 63.52 (CH_2_CF_3_), 103.45, 121.46 (CH_2_ CF_3_), 124.21 (CH_2_ CF_3_), 126.96 (CH_2_ CF_3_), 127.02, 129.70 (CH_2_ CF_3_), 139.93, 145.03, 153.81, 154.43. HRMS (ESI) m/z: calculated for C_15_H_21_F_3_N_5_O [M + H]^+^: 344.1693; found 344.1704.

**[28b]**: 20 mg, yield 16%. White solid, m.p. 144–146 °C (Et_2_O/petroleum ether). ^1^H NMR (400 MHz, methanol‐*d*_4_) δ 2.27 (s, 3H, piperazine‐CH_3_), 2.38–2.70 (m, 8H, piperazine H), 3.85 (s, 2H, CH_2_‐piperazine), 4.12 (s, 3H, imidazole‐CH_3_), 5.05 (q, 2H, *J* = 8.7 Hz, OCH_2_CF_3_), 7.27 (d, 1H, *J* = 5.8 Hz, H‐7), 7.86 (d, 1H, *J* = 5.8 Hz, H‐6). ^13^C NMR (100 MHz, methanol‐*d*_4_) δ 33.61, 46.08, 53.86, 55.41, 55.97, 62.58 (CH_2_CF_3_), 62.94 (CH_2_CF_3_), 63.29 (CH_2_CF_3_), 63.65 (CH_2_CF_3_), 111.15, 121.43 (CH_2_ CF_3_), 122.27, 124.18 (CH_2_ CF_3_), 126.93 (CH_2_ CF_3_), 129.67 (CH_2_ CF_3_), 139.08, 150.56, 150.63, 155.80. HRMS (ESI) m/z: calculated for C_15_H_21_F_3_N_5_O [M + H]^+^: 344.1693; found 344.1681.

#### 4.1.26 1‐Isopropyl‐2‐((4‐methylpiperazin‐1‐yl)methyl)‐4‐(2,2,2‐trifluoroethoxy)‐1*H*‐imidazo[4,5‐*c*]pyridine (29a) and 3‐isopropyl‐2‐((4‐methylpiperazin‐1‐yl)methyl)‐4‐(2,2,2‐trifluoroethoxy)‐3*H*‐imidazo[4,5‐*c*]pyridine (29b)

These target compounds were synthesized by following an analogous procedure to that described for the preparation of the derivatives **25a** and **25b**, upon reaction of compound **22** (120 mg, 0.36 mmol) with 2‐bromopropane. After the work‐up of the reaction, the residue was subjected to column chromatography (silica gel), using a mixture of dichloromethane/methanol as the eluent (from 95/5 up to 90/10, v/v), to provide a mixture of the two regioisomers **29a** and **29b**. The latter was separated by a second column chromatography, using basic aluminum oxide as the stationary phase and a mixture of dichloromethane/ethyl acetate as the eluent (from 100/0 up to 0/100, v/v), to yield the pure target compounds **29a** and **29b**.

**[29a]**: 45 mg, yield 33%. White solid, m.p. 164–165 °C (CH_2_Cl_2_/Et_2_O). ^1^H NMR (400 MHz, CDCl_3_) δ 1.60 (d, 6H, *J* = 6.8 Hz, isopropyl‐CH_3_), 2.34 (s, 3H, piperazine‐CH_3_), 2.43 – 2.65 (m, 8H, piperazine H), 3.85 (s, 2H, CH_2_‐piperazine), 4.94–5.05 (m, 3H, OCH_2_CF_3_, isopropyl‐CH), 7.16 (d, 1H, *J* = 5.9 Hz, H‐7), 7.84 (d, 1H, *J* = 5.9 Hz, H‐6). ^13^C NMR (100 MHz, CDCl_3_) δ 21.55, 45.65, 48.81, 52.53, 54.97, 55.56, 61.21 (CH_2_CF_3_), 61.57 (CH_2_CF_3_), 61.93 (CH_2_CF_3_), 62.29 (CH_2_CF_3_), 104.06, 119.69 (CH_2_ CF_3_), 122.45 (CH_2_ CF_3_), 125.21 (CH_2_ CF_3_), 127.73, 127.98 (CH_2_ CF_3_), 137.91, 141.08, 150.80, 153.99. HRMS (ESI) m/z: calculated for C_17_H_25_F_3_N_5_O [M + H]^+^: 372.2006; found 372.1992.

**[29b]**: 40 mg, yield 30%, gum. ^1^H NMR (400 MHz, CDCl_3_) δ 1.62 (d, 6H, *J* = 6.9 Hz, isopropyl‐CH_3_), 2.29 (s, 3H, piperazine‐CH_3_), 2.35–2.65 (m, 8H, piperazine H), 3.83 (s, 2H, CH_2_‐piperazine), 4.93 (q, 2H, *J* = 8.5 Hz, OCH_2_CF_3_), 5.04 – 5.12 (m, 1H, isopropyl‐CH), 7.33 (d, 1H, *J* = 5.6 Hz, H‐7), 7.86 (d, 1H, *J* = 5.6 Hz, H‐6). ^13^C NMR (100 MHz, CDCl_3_) δ 22.05, 45.95, 49.56, 52.98, 55.13, 56.32, 61.97 (CH_2_CF_3_), 62.32 (CH_2_CF_3_), 62.68 (CH_2_CF_3_), 63.04 (CH_2_CF_3_), 111.01, 119.48, 119.89 (CH_2_ CF_3_), 122.64 (CH_2_ CF_3_), 125.39 (CH_2_ CF_3_), 128.14 (CH_2_ CF_3_), 137.51, 147.55, 151.40, 153.43. HRMS (ESI) m/z: calculated for C_17_H_25_F_3_N_5_O [M + H]^+^: 372.2006; found 372.1997.

#### 4.1.27 1‐Cyclopentyl‐2‐((4‐methylpiperazin‐1‐yl)methyl)‐4‐(2,2,2‐trifluoroethoxy)‐1*H*‐imidazo[4,5‐*c*]pyridine (30a) and 3‐cyclopentyl‐2‐((4‐methylpiperazin‐1‐yl)methyl)‐4‐(2,2,2‐trifluoroethoxy)‐3*H*‐imidazo[4,5‐*c*]pyridine (30b)

These target compounds were synthesized by following an analogous procedure to that described for the preparation of the derivatives **25a** and **25b**, upon reaction of compound **22** (120 mg, 0.36 mmol) with bromocyclopentane. After the work‐up of the reaction, the residue was subjected to column chromatography (silica gel), using a mixture of dichloromethane/methanol as the eluent (from 98/2 up to 90/10, v/v), to provide a mixture of the two regioisomers **30a** and **30b**. The latter was separated by a second column chromatography, using basic aluminum oxide as the stationary phase and a mixture of dichloromethane/ethyl acetate as the eluent (from 95/5 up to 0/100, v/v), to yield the pure target compounds **30a** and **30b**.

**[30a]**: 45 mg, yield 31%. Beige solid, m.p. 133–135 °C (CH_2_Cl_2_/*n*‐pentane). ^1^H NMR (400 MHz, CDCl_3_) δ 1.74–1.86 (m, 2H, cyclopentyl H), 1.97–2.18 (m, 6H, cyclopentyl H), 2.27 (s, 3H, piperazine‐CH_3_), 2.34–2.60 (m, 8H, piperazine H), 3.85 (s, 2H, CH_2_‐piperazine), 4.99 (q, 2H, *J* = 8.6 Hz, OCH_2_CF_3_), 5.16 (p, 1H, *J* = 8.8 Hz, cyclopentyl H‐1′), 7.06 (d, 1H, *J* = 5.8 Hz, H‐7), 7.83 (d, 1H, *J* = 5.8 Hz, H‐6). ^13^C NMR (100 MHz, CDCl_3_) δ 25.36, 30.84, 45.31, 52.02, 54.80, 55.33, 57.29, 61.23 (CH_2_CF_3_), 61.59 (CH_2_CF_3_), 61.95 (CH_2_CF_3_), 62.31 (CH_2_CF_3_), 103.72, 119.68 (CH_2_ CF_3_), 122.44 (CH_2_ CF_3_), 125.20 (CH_2_ CF_3_), 127.73, 127.96 (CH_2_ CF_3_), 137.93, 140.75, 151.35, 154.04. HRMS (ESI) m/z: calculated for C_19_H_27_F_3_N_5_O [M + H]^+^: 398.2163; found 398.2154.

**[30b]**: 30 mg, yield 21%. Beige solid, m.p. 188–189 °C (CH_2_Cl_2_/*n*‐pentane). ^1^H NMR (400 MHz, CDCl_3_) δ 1.66–1.77 (m, 2H, cyclopentyl H), 1.98–2.10 (m, 4H, cyclopentyl H), 2.22–2.33 (m, 5H, cyclopentyl H, piperazine‐CH_3_), 2.37–2.65 (m, 8H, piperazine H), 3.85 (s, 2H, CH_2_‐piperazine), 4.96 (q, 2H, *J* = 8.7 Hz, OCH_2_CF_3_), 5.03–5.13 (m, 1H, cyclopentyl H‐1′), 7.34 (d, 1H, *J* = 5.6 Hz, H‐7), 7.86 (d, 1H, *J* = 5.6 Hz, H‐6). ^13^C NMR (100 MHz, CDCl_3_) δ 23.52, 32.00, 45.81, 52.74, 55.14, 56.26, 58.76, 61.73 (CH_2_CF_3_), 62.09 (CH_2_CF_3_), 62.45 (CH_2_CF_3_), 62.80 (CH_2_CF_3_), 111.11, 119.59, 119.82 (CH_2_ CF_3_), 122.58 (CH_2_ CF_3_), 125.34 (CH_2_ CF_3_), 128.09 (CH_2_ CF_3_), 137.49, 147.74, 151.54, 154.10. HRMS (ESI) m/z: calculated for C_19_H_27_F_3_N_5_O [M + H]^+^: 398.2163; found 398.2152.

### 4.2 Biology

#### 4.2.1 Cells and Viral Constructs

Huh5−2 cells (RRID: N/A), which stably express the subgenomic HCV reporter replicon I389luc‐ubi‐neo/NS3‐3′/Con1/5.1 (genotype 1b, strain Con1), were established as previously described [40] and kindly provided by Dr. R. Bartenschlager (Department of Infectious Diseases, Heidelberg University, Germany). Cells were cultured in high‐glucose (25 mM) Dulbecco's modified minimal essential medium (Invitrogen, Waltham, MA, USA), containing 10% (v/v) fetal calf serum, 2 mM L‐glutamine, 100 U/mL penicillin, 100 µg/mL streptomycin, and 0.1 mM nonessential amino acids (referred to as complete DMEM). Geneticin (G418) (500 μg/mL) was used to select the cells that stably express the HCV viral replicon.

#### 4.2.2 Cytotoxicity Assays

The cytotoxicity of each compound was evaluated using the AlamarBlue assay, which employs a redox‐sensitive indicator, the color of which changes when it is reduced by cellular metabolic activity. Huh5.2 cells were seeded in 96‐well plates at a density of 10^4^ cells per well and incubated for 24 h in 200 μl of complete DMEM, at 37 °C (5% CO_2_). Then, the culture medium was replaced with 100 μl of complete DMEM containing different concentrations of the DMSO‐diluted compound (22, 66, 200 μM) or DMSO (mock‐treated cells used as control). After 72 h of incubation of cells at 37 °C (5% CO_2_), 10 μl of AlamarBlue reagent was added to each well and immediately afterward, the absorbance was measured at 570 and 600 nm, using a Bio‐Rad Model 680 plate reader. Following absorbance measurement, the cells were incubated for 4 h and then the absorbance was again measured, as before. Nonlinear regression analysis was performed using Prism 9.0 (GraphPad Software Inc., San Diego, CA, USA), correlating the logarithmic concentrations of each compound with the respective reductions of the indicator during the 4 h period, compared to that of mock‐treated cells, to determine the CC_50_ value of each compound.

#### 4.2.3 Cell‐Based Antiviral Assay

The relative viral replication levels were determined based on the activity of F‐Luciferase, which is expressed by the HCV replicon. The activity of F‐Luciferase was measured using the Luciferase Assay System (Promega Corporation, Madison, WI, USA). Huh5.2 cells were seeded in 96‐well plates at a density of 10^4^ cells per well and incubated for 24 h in 200 μl of complete DMEM, at 37 °C (5% CO_2_). Then, the culture medium was replaced with 100 μl of complete DMEM containing different noncytotoxic concentrations (3.125, 6.25, 12.5, 25 μM) of DMSO‐diluted compounds or DMSO (mock‐treated cells used as control). After 72 h of incubation at 37 °C with 5% CO_2_, the cells were lysed, and the activity of F‐Luciferase was measured in the GloMax 20/20 single‐tube luminometer (Promega Corporation), for 10s in accordance with the manufacturer's protocol. To normalize luciferase activity, total protein concentrations of the lysates were quantified using the Bradford assay (Bio‐Rad, Hercules, CA, USA). Nonlinear regression analysis was performed using Prism 9.0 (GraphPad Software Inc., San Diego, CA, USA), plotting the logarithm of the tested concentrations of the compound against the percentage levels of reduction in F‐luciferase activity, relative to the control, to determine the EC_50_ values. The selectivity index (SI) was calculated as the ratio of CC_50_ to EC_50_.

#### 4.2.4 Total RNA Extraction and Quantification of Viral Replicons

Huh5.2 cells were seeded into 12‐well flat‐bottom plates at a density of 0.5 × 10^6^ cells per well, using 1.5 ml of complete DMEM per well. Following 24 h of incubation at 37 °C (5% CO_2_), the culture medium was replaced with 500 μl of complete DMEM containing different noncytotoxic concentrations (3.125, 6.25, 12.5, 25 μM) of the DMSO‐diluted compound or DMSO (mock‐treated cells used as control). After a 72 h incubation at 37 °C (5% CO_2_), the cells were lysed using TRIzol reagent (Thermo Fisher Scientific, Waltham, MA, USA) and total RNA was extracted according to the manufacturer's protocol. Reverse transcription (RT) was carried out using Moloney Murine Leukemia Virus (MMLV) reverse transcriptase (Promega), using gene‐specific reverse primers for Con1 IRES and the housekeeping gene YWHAZ, used for normalization (Con1‐IRES‐R: 5′‐GGATTCGTGCTCATGGTGCA‐3′, YWHAZ‐R: 5′‐GGATGTGTTGGTTGCATTTCCT‐3′). Following RT, qPCR was performed using the Universal qPCR Master Mix Luna (New England Biolabs, Inc., Ipswich, MA, USA) and forward and reverse primers for Con1 IRES and YWHAZ (Con1‐IRES‐F: 5′‐GGCCTTGTGGTACTGCCTGATA‐3′, YWHAZ‐F: 5′‐GCTGGTGATGACAAGAAAGG‐3′, reverse primers same as above).

#### 4.2.5 Gel Electrophoresis and Western Blot Analysis

For quantification of viral protein expression, Huh5.2 cells were seeded in 6‐well plates at a density of 0.3 × 10^6^ cells, using 3 mL of complete DMEM per well. Following 24 h of incubation at 37 °C (5% CO_2_), the medium was replaced with 1 ml of complete DMEM containing different noncytotoxic concentrations (25, 12.5, 6.25, 3.125 μM) of DMSO‐diluted compounds or DMSO (mock‐treated cells used as control). After 72 h of incubation at 37 °C (5% CO_2_), the cells were harvested, lysed, and after protein quantification, the cell extracts were analyzed on a 13% SDS/PAGE gel as described previously [44]. The following antibodies were used for immunoblotting: HCV NS5A (9E10) monoclonal antibody (kindly provided by Prof. C.M. Rice) at a dilution of 1:2000, β‐actin monoclonal antibody (Merck‐Millipore) at 1:6000, and secondary anti‐mouse horseradish peroxidase‐conjugated antibody (Cell Signaling) at 1:2000.

#### 4.2.6 Indirect Immunofluorescence Assays

Indirect immunofluorescence assays were performed using the anti‐NS5A monoclonal antibody (9E10; kindly provided by Prof. C. Rice), as previously described [45]. An Alexa Fluor 488–conjugated goat anti‐mouse IgG antibody was used as secondary antibody. Alexa Fluor 488 fluorescence intensity was measured using a Spark Cyto hybrid spectrophotometer/imager (Tecan) and normalized to the total number of cells, as determined by the staining of the cell's nucleus using DAPI (1:10,000 dilution). Images were acquired using the same instrument, and ten images were collected for each experimental condition.

#### 4.2.7 Statistical Analysis

In all diagrams, bars represent mean values of at least three independent experiments performed in triplicate. Error bars represent standard deviation. The statistical analysis of the presented results was conducted using One‐Way ANOVA, followed by the appropriate post hoc analysis for multiple comparisons using GraphPad Prism, version 9.0.0 (GraphPad Software Inc., San Diego, CA, USA). Statistical significance was set at *p* < 0.05.

### 4.3 In Silico Studies of the NS4B – Compound Interaction

#### 4.3.1 Protein Structure Preparation

The three‐dimensional structure of the HCV NS4B was obtained through homology modeling using ColabFold [46], a platform that accelerates protein structure prediction by integrating the rapid homology search capabilities of MMseqs2 with the advanced folding algorithms AlphaFold2 and RoseTTAFold. The model includes four predicted transmembrane helices (TM1–TM4), N‐ and C‐terminal amphipathic helices (AH1 and AH2), and conserved functional regions such as the Walker A NTP‐binding motif (residues 129–135) and arginine‐rich RNA‐binding segments in the C‐terminus. The resulting NS4B model was subsequently inspected and annotated in light of published topology and functional studies, ensuring that the positions of AH1–AH2, TM1–TM4 and helices H1–H2 were consistent with experimentally supported models of NS4B organization [21, 22, 23, 24]. No manual reshaping of the binding cavity was performed; instead, docking calculations for both the newly synthesized imidazo[4,5‐*c*]pyridine derivatives and three reference NS4B‐targeting agents (amphihevir, anguizole and clemizole) were carried out on the same model, and their top‐scoring poses converged within a common hydrophobic pocket, indicating that this site can accommodate both our ligands and known NS4B inhibitors in a coherent manner. The structure was optimized for docking using the UCSF Chimera software package [47]. NS4B was modeled in its predominant protonation state at physiological pH, with residues embedded in the transmembrane core kept neutral.

#### 4.3.2 Ligand Preparation

A series of synthetic compounds (**8**, **23**, **22**, **30a/b**, **28a/b**, **21**, **29a/b**, **25a/b**, **26a/b**, **24a/b**, **27a/b**) were chemically designed, and their three‐dimensional structures were generated using ChemDraw. The 3D structures of all compounds were generated and geometrically optimized to obtain their most stable (lowest energy) conformations. Initial conformational searches were performed using the Monte Carlo method and the MMFF94 molecular mechanics force field, as implemented in the Spartan’14 Molecular Modeling suite. Geometry optimization of the lowest‐energy conformers was further refined through quantum chemical calculations using the Hartree–Fock method with the 6‐31G* basis set in the gas phase. All ligands were modeled in their predominant protonation state at physiological pH (7.4), and the geometries of the lowest‐energy conformers were subsequently refined by quantum‐chemical calculations using the Hartree–Fock method with the 6‐31G* basis set in the gas phase.

#### 4.3.3 Molecular Docking

Blind molecular docking was performed using the CB‐Dock2 web server, which integrates cavity detection, docking, and homologous template fitting to predict ligand binding sites and poses in protein targets [48]. The NS4B protein structure was submitted without predefined binding sites to allow unbiased identification of potential ligand‐binding cavities. For each ligand, the top five docking poses were generated, and the pose with the highest docking score (lowest binding free energy) was selected for further analysis. The docking scores (kcal/mol) and interacting residues within 4 Å of the ligand were recorded.

#### 4.3.4 Analysis of Docking Results

Protein–ligand interactions, including hydrogen bonds, hydrophobic and electrostatic interactions, were visualized and analyzed using UCSF Chimera and LigPlot+ [49]. The localization of ligand binding sites was mapped onto the NS4B structural domains to assess the functional relevance of each interaction.

#### 4.3.5 Structural Characterization of NS4B

The initially generated structural model of NS4B was subsequently refined to align with the reported topology in the literature [22]. Key structural features—including transmembrane domains, amphipathic helices, conserved motifs, and the flexible loop region were manually annotated and adjusted based on published data [22]. These refinements enabled the model to more accurately reflect established domain boundaries and membrane‐associated elements. The final model was used to identify functionally relevant regions and to support the interpretation of molecular docking results.

## Conflicts of Interest

The authors declare no conflicts of interest.

## Supporting information

Supplementary Material

## Data Availability

The data that support the findings of this study are available in the Supporting Information of this article.
