# Supplementary material for: Design and Synthesis of Novel Imidazo[4,5‐c]pyridine Derivatives, Evaluation of Their Activity Against Hepatitis C Virus and In Silico Prediction of Their Binding Mode to NS4B Protein
Source: ChemMedChem. 2026 Jul 13;21(13):e70378. doi: 10.1002/cmdc.70378 (PMC13363010; doi:10.1002/cmdc.70378)

Design and Synthesis of Novel Imidazo[4,5-*c*]pyridine Derivatives, Evaluation of their Activity Against Hepatitis C Virus and In Silico Prediction of their Binding Mode to NS4B Protein

Eftychia Karampella[1,#], George Mpekoulis[2,#], Maria Georgiou[1], Iris Kolida[2], Katerina I. Kalliampakou[2], Eirini P. Kollia[2], Eleni V. Mikropoulou[3], Maria Halabalaki[3], Christos T. Chasapis[4], Niki Vassilaki[2,*], Nikolaos Lougiakis[1,*]

[1] Laboratory of Medicinal Chemistry, Division of Pharmaceutical Chemistry, Department of Pharmacy, School of Health Sciences, National and Kapodistrian University of Athens, Panepistimiopolis Zografou, 15771 Athens, Greece.
[2] Molecular Virology Laboratory, Hellenic Pasteur Institute, 11521, Athens, Greece.
[3] Division of Pharmacognosy and Natural Products Chemistry, Department of Pharmacy, School of Health Sciences, National and Kapodistrian University of Athens, Panepistimiopolis Zografou, 15771 Athens, Greece.
[4] Laboratory of Organic Chemistry, Department of Chemistry, National and Kapodistrian University of Athens, 12 Panepistimiopolis Zografou, 15771 Athens, Greece

[#] The contribution to this work was equal among these authors and should be considered as joint first authors

* Correspondence: Nikolaos Lougiakis (nlougiak@pharm.uoa.gr), Niki Vassilaki (nikiv@pasteur.gr)

Table S1. Docking Scores of tested compounds with the HCV NS4B protein.

Compound	Docking Score (kcal/mol)
**23**	−7.5
**30a**	−7.5
**29a**	−7.5
**27a**	−7.5
**27b**	−7.4
**29b**	−7.1
**28a**	−6.9
**25b**	−6.9
**26a**	−6.9
**25a**	−6.8
**28b**	−6.7
**22**	−6.7
**24a**	−6.7
**30b**	−6.6
**24b**	−6.5
**26b**	−6.5
**8**	−6.0
**21**	−5.9

Table S2. Summary of key interactions for selected ligands within the NS4B binding cavity.

Compound	Docking score (kcal/mol)	H-bonds (n) / main partner	Main hydrophobic contact residues (within ~4 Å)
**23**	−7.5	1 (Asp139)	Leu79, Phe82, Thr83, Gly143, Gly147, Ala180, Val183, Ser177, Ser213, Ile140
**27a**	−7.5	0	Leu79, Phe82, Thr83, Asp139, Ile140, Tyr144, Gly143, Gly147, Ala180, Val148, Val183, Ser213
**29a**	−7.5	0	Ala 121, Ser213, Leu79, Ala212, Phe82, Val183, Ile209, Ile140, Try144, Gly143, Thr83, Ala180
**30a**	−7.5	0	Ile86, Ile140, Gly143, Thr83, Phe82, Ser177, Ala180, Asp139, Tyr144, Leu79, Ile183, Gly147
**30b**	−6.6	1 (Gln203)	Asn216, Ser113, His222, Asn73, Asn206, Pro74, Gly72, Ser219
**26b**	−6.5	0	Val183, Ser213, Tyr144, Ala212, Gly147, Leu79, Phe82, Ile140, Gly143, Ile86, Asp139, Thr83

Figure S1. Copies of ^{1}H- and ^{13}C-NMR spectra of all the novel derivatives.

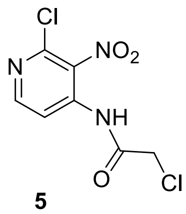

5

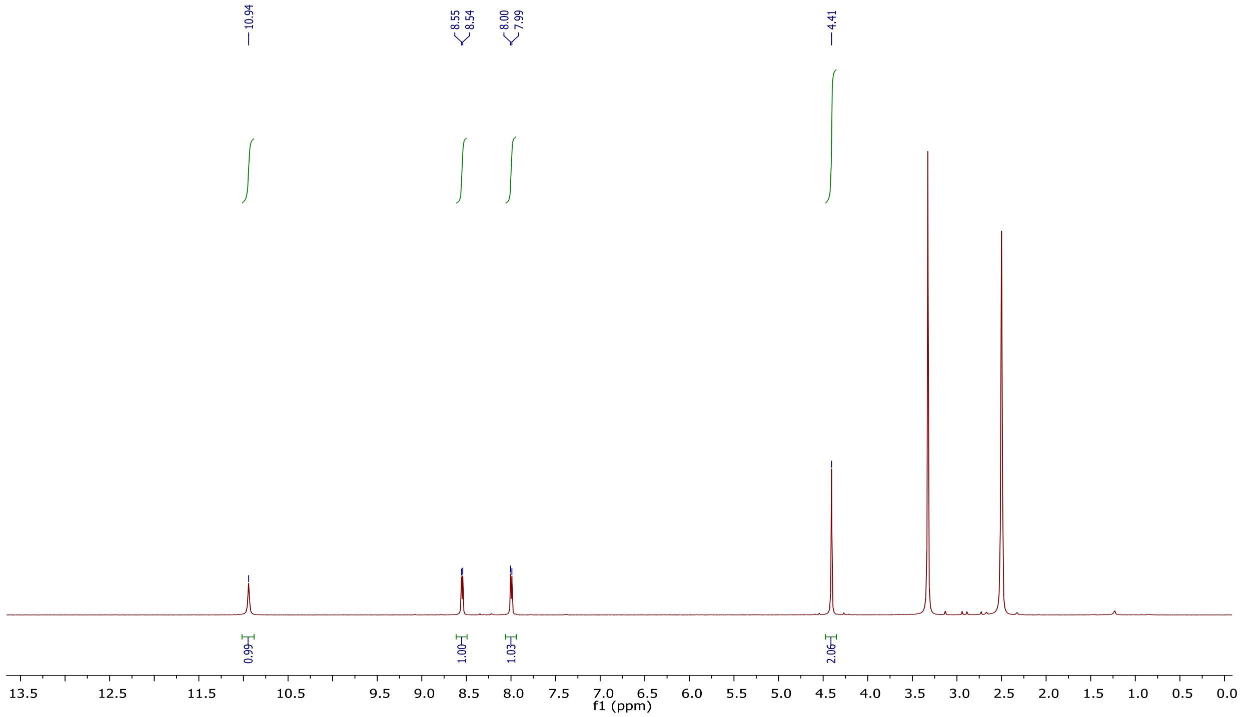

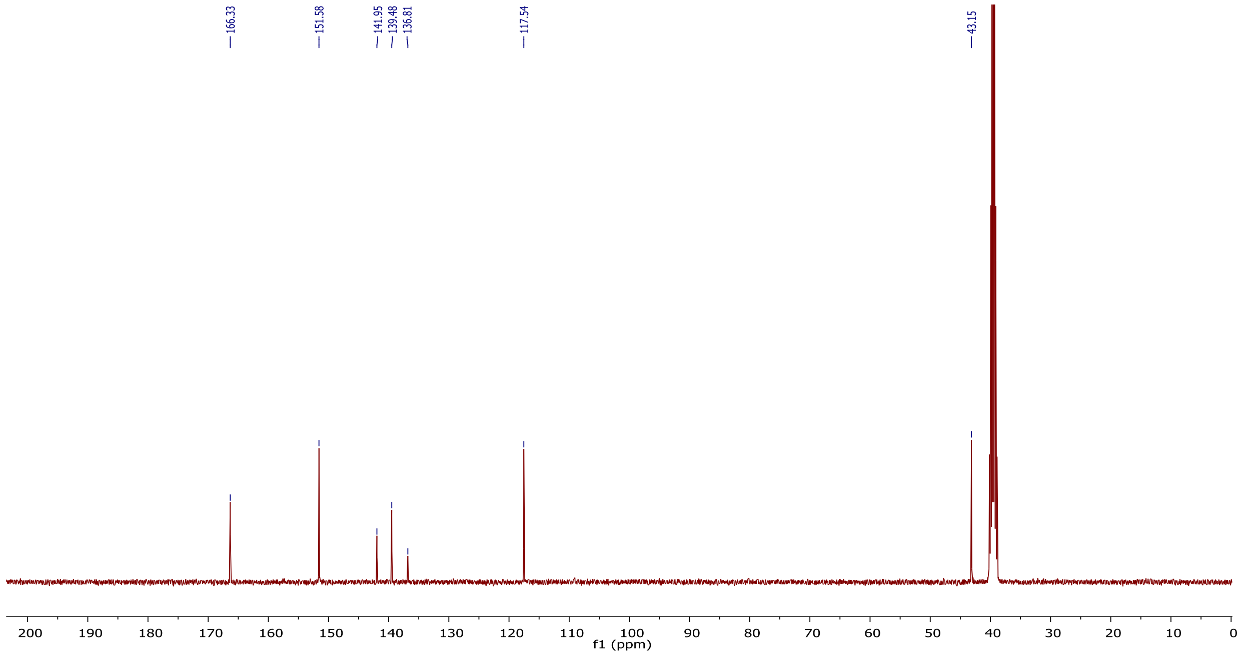

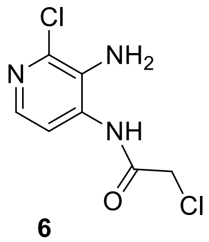
Cl
NH2
N
NH
O
Cl

6

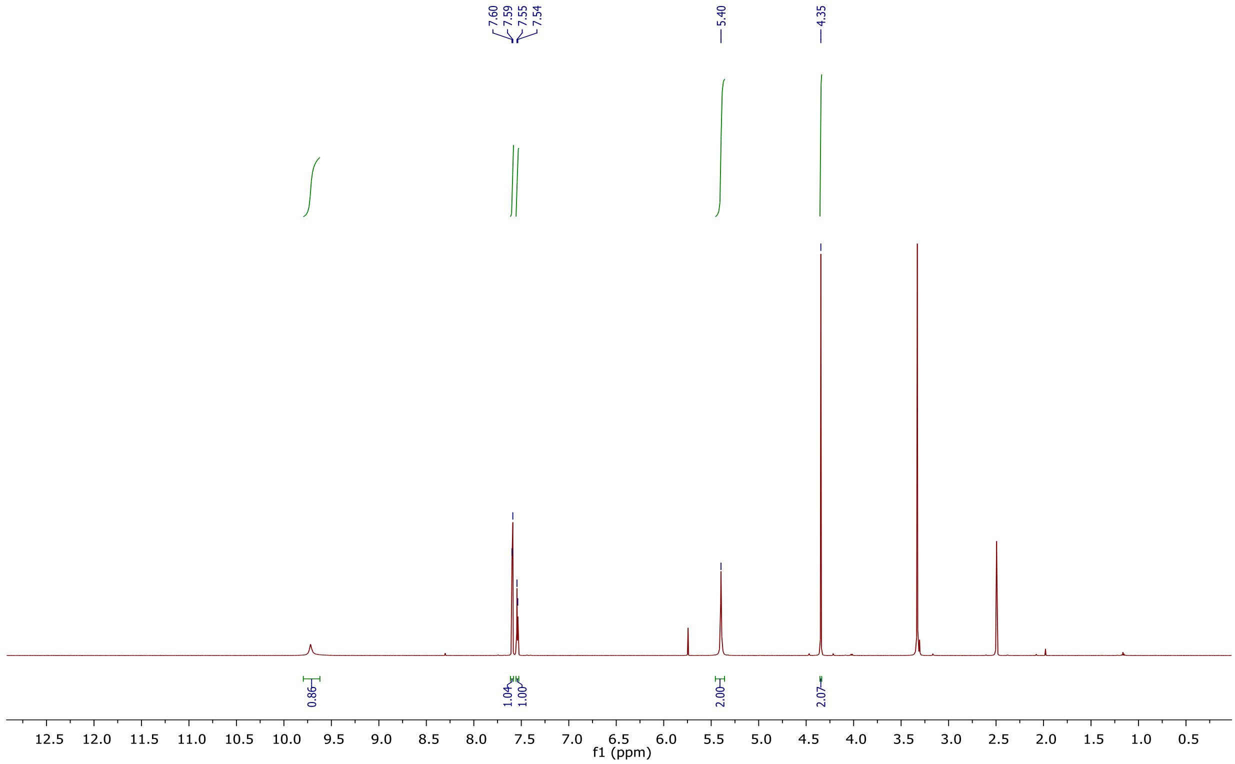
7.60
7.59
7.55
7.54
5.40
4.35
0.86
1.04
1.00
2.00
2.07
12.5 12.0 11.5 11.0 10.5 10.0 9.5 9.0 8.5 8.0 7.5 7.0 6.5 6.0 5.5 5.0 4.5 4.0 3.5 3.0 2.5 2.0 1.5 1.0 0.5
f1 (ppm)

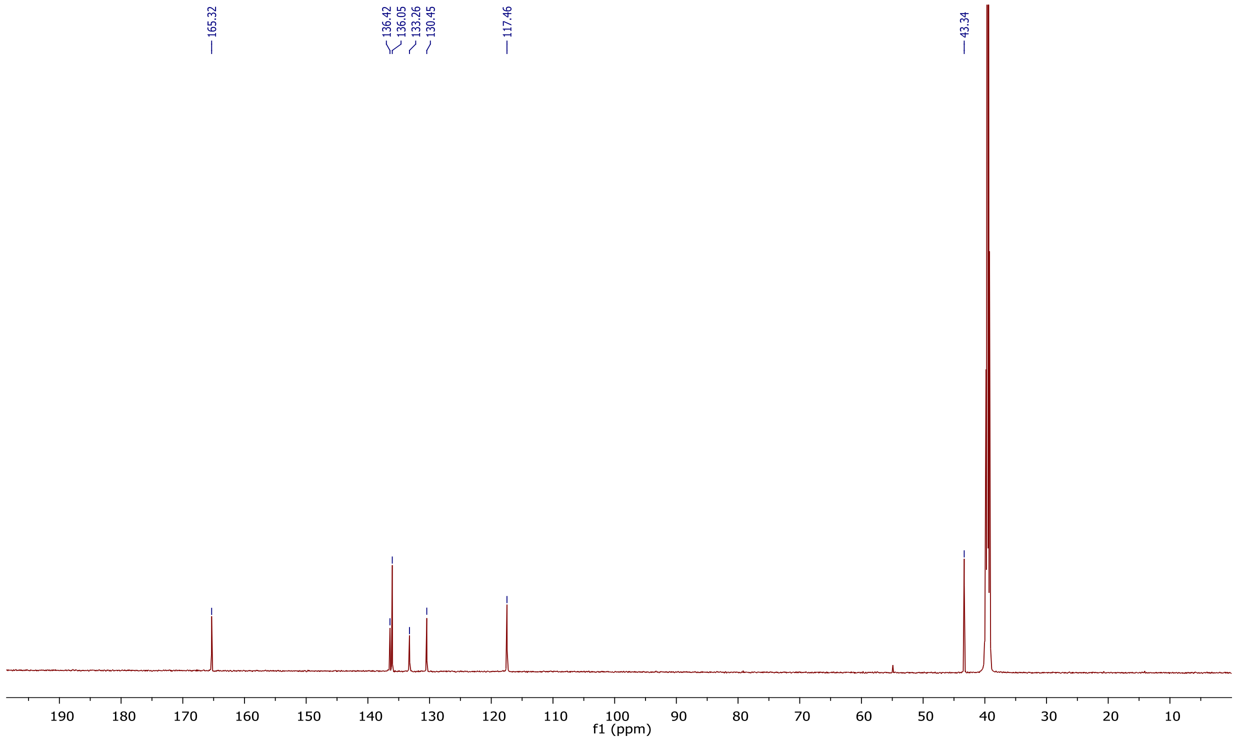
165.32
136.42
136.05
133.26
130.45
117.46
43.34
190 180 170 160 150 140 130 120 110 100 90 80 70 60 50 40 30 20 10
f1 (ppm)

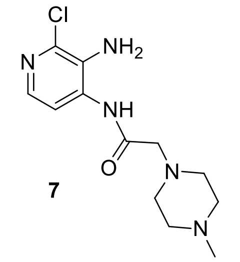
Cl
NH2
N
NH
O
N
N

7

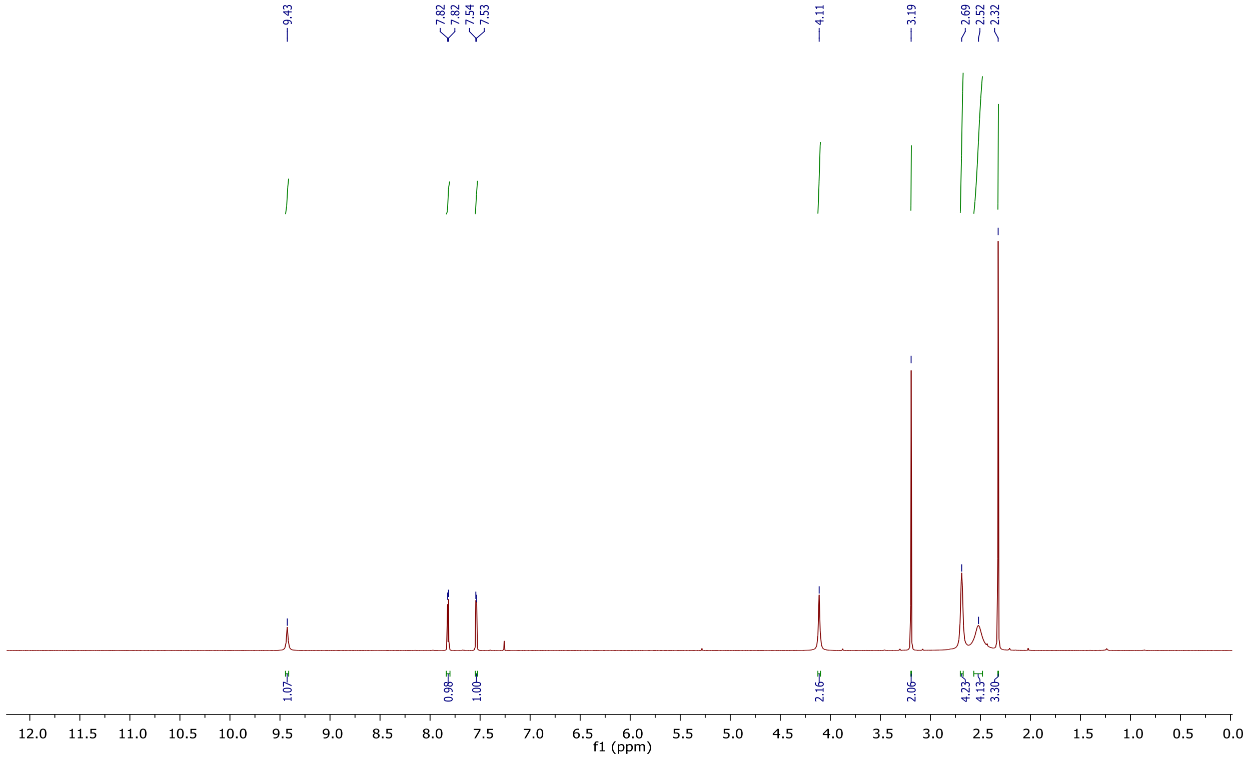
9.43
7.82
7.82
7.54
7.53
4.11
3.19
2.69
2.52
2.32
1.07
0.98
1.00
2.16
2.06
4.23
4.13
3.30
12.0 11.5 11.0 10.5 10.0 9.5 9.0 8.5 8.0 7.5 7.0 6.5 6.0 5.5 5.0 4.5 4.0 3.5 3.0 2.5 2.0 1.5 1.0 0.5 0.0
f1 (ppm)

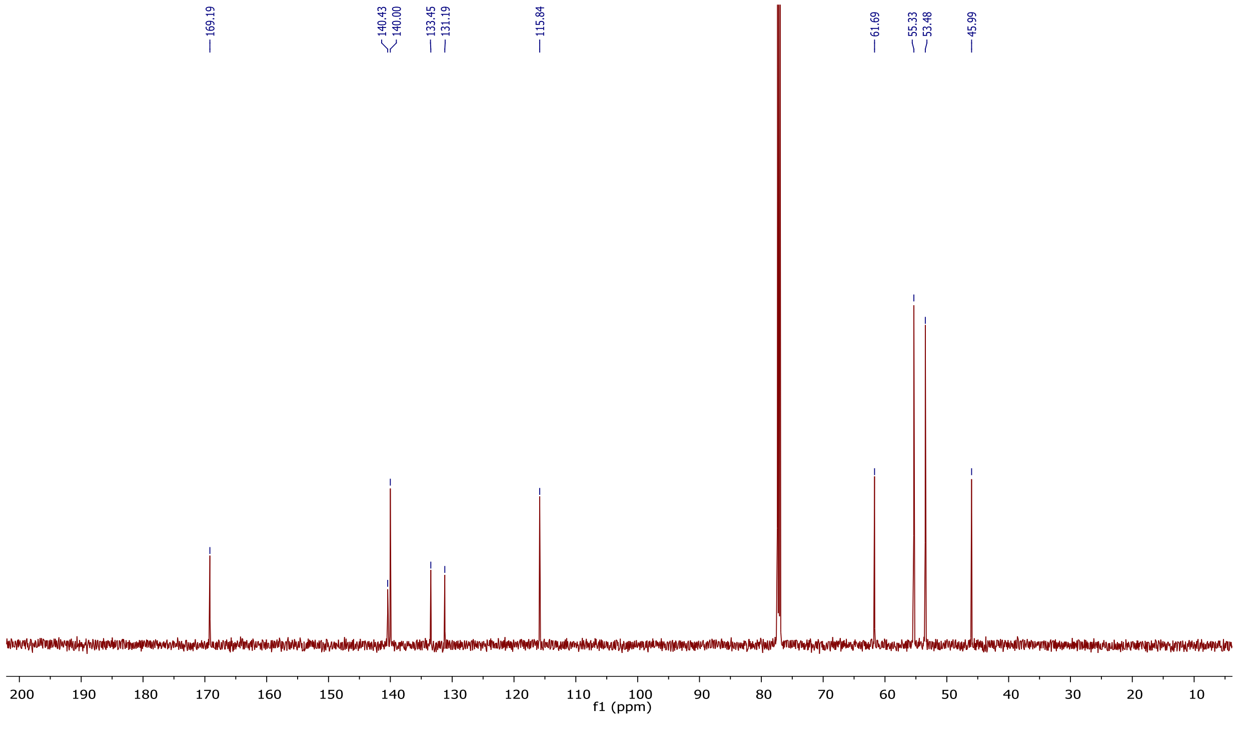
169.19
140.43
140.00
133.45
131.19
115.84
61.69
55.33
53.48
45.99
200 190 180 170 160 150 140 130 120 110 100 90 80 70 60 50 40 30 20 10
f1 (ppm)

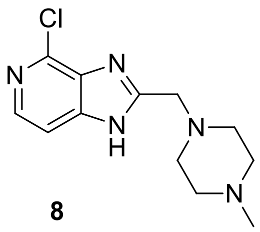
Cl
N
N
N
H
N
N

8

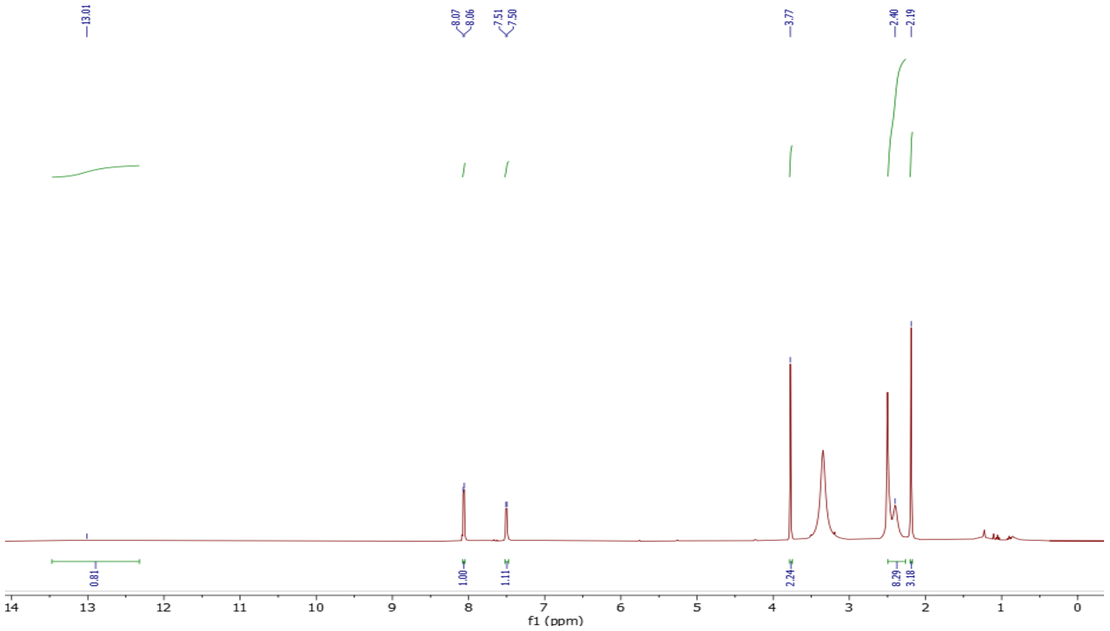
13.01
8.07
8.06
7.51
7.50
3.77
2.40
2.19
0.81
1.00
1.11
2.24
8.29
3.18
14 13 12 11 10 9 8 7 6 5 4 3 2 1 0
f1 (ppm)

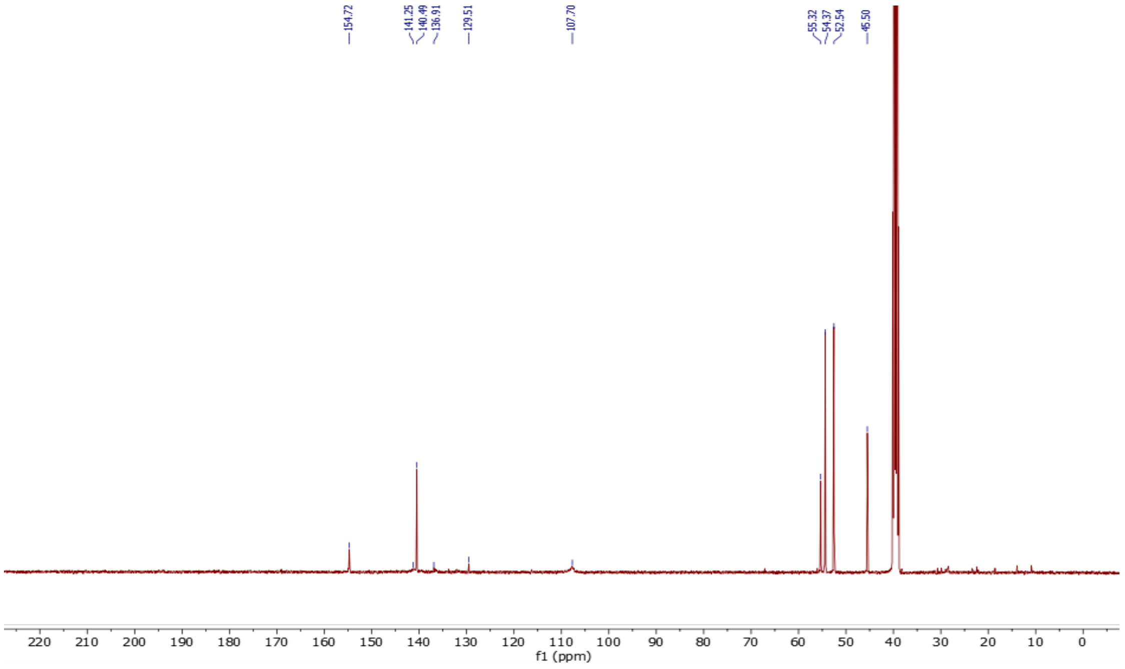
154.72
141.25
140.49
136.91
129.51
107.70
55.32
54.37
52.54
45.50
220 210 200 190 180 170 160 150 140 130 120 110 100 90 80 70 60 50 40 30 20 10 0
f1 (ppm)

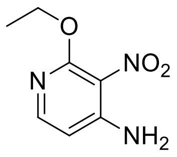
O
NO2
N
NH2

9

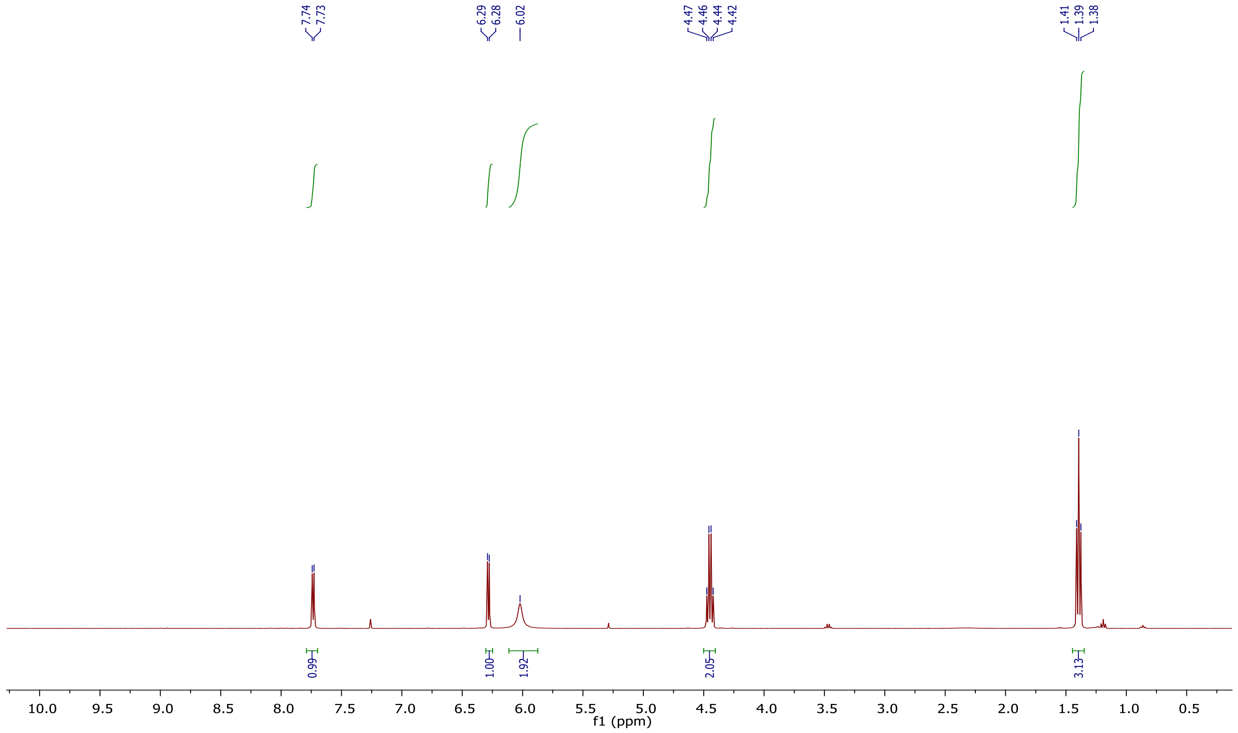
7.74
7.73
6.29
6.28
6.02
4.47
4.46
4.44
4.42
1.41
1.39
1.38
0.99
1.00
1.92
2.05
3.13
10.0 9.5 9.0 8.5 8.0 7.5 7.0 6.5 6.0 5.5 5.0 4.5 4.0 3.5 3.0 2.5 2.0 1.5 1.0 0.5
f1 (ppm)

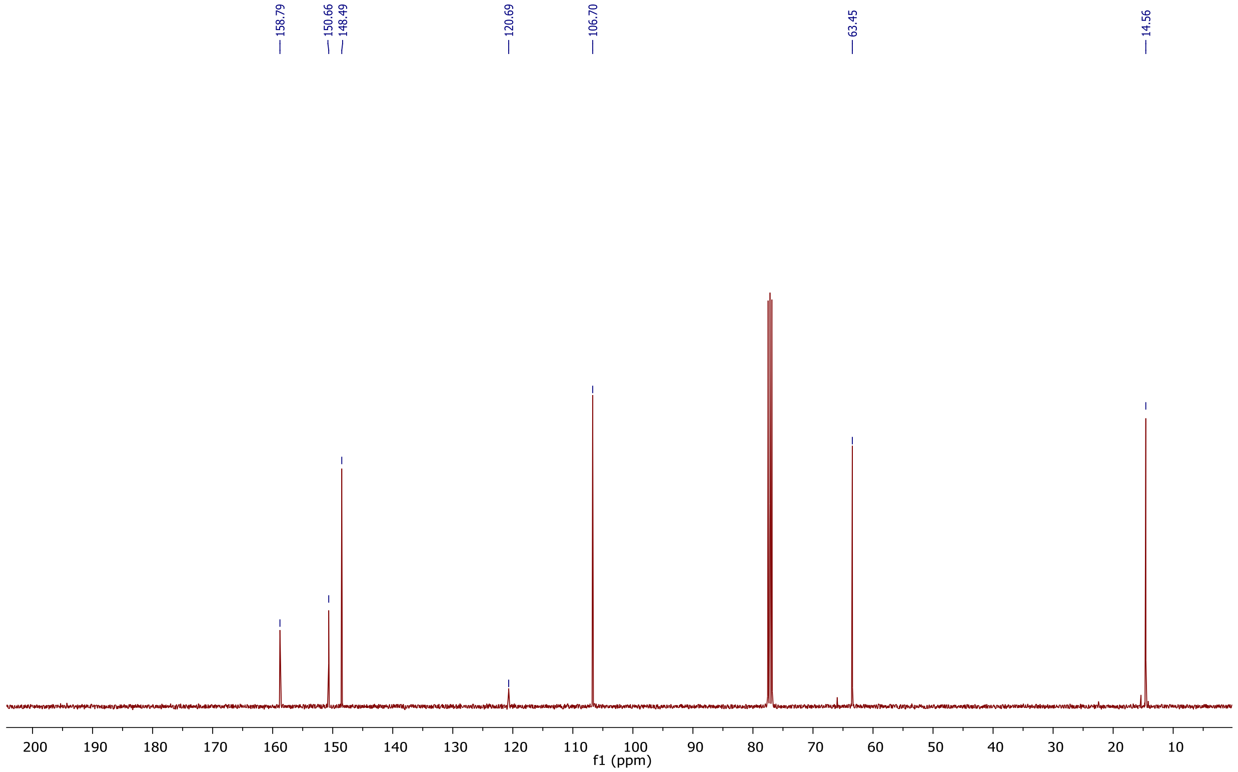
158.79
150.66
148.49
120.69
106.70
63.45
14.56
200 190 180 170 160 150 140 130 120 110 100 90 80 70 60 50 40 30 20 10
f1 (ppm)

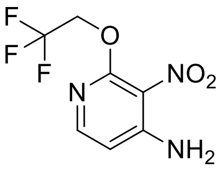
F
F
F
O
N
NO2
NH2

10

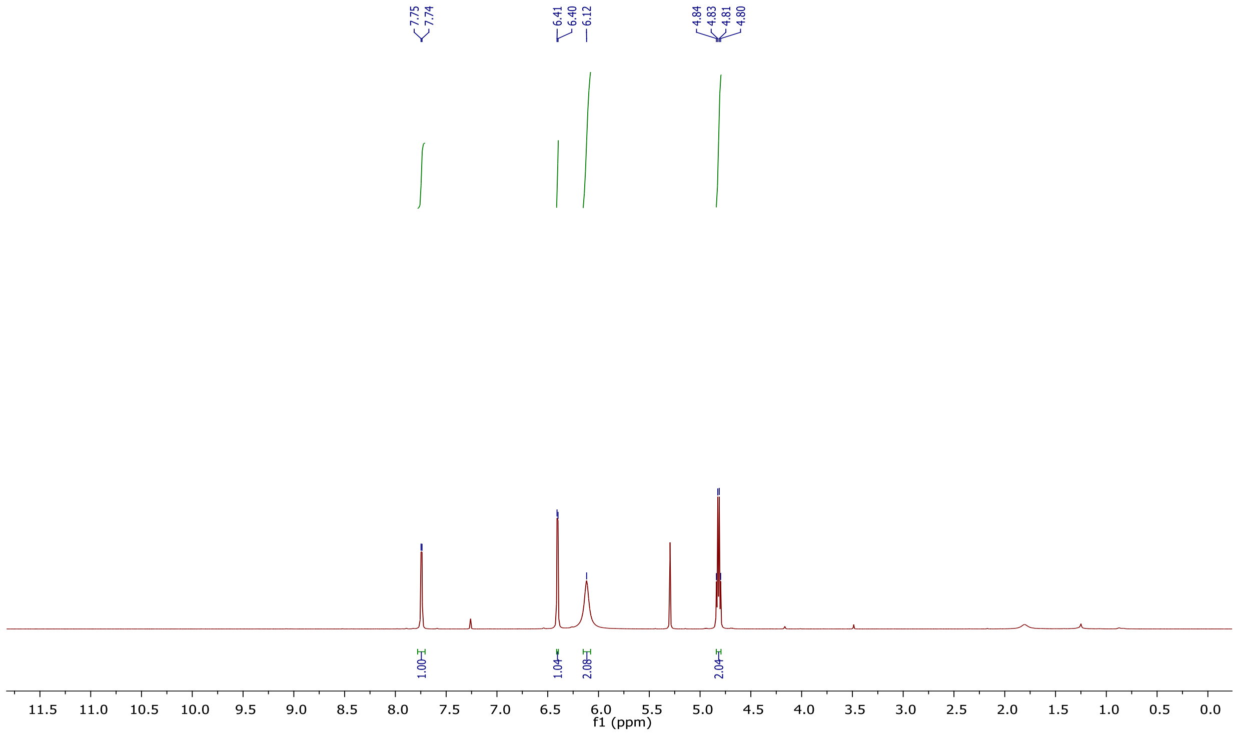
7.75
7.74
6.41
6.40
6.12
4.84
4.83
4.81
4.80
1.00
1.04
2.08
2.04
f1 (ppm)

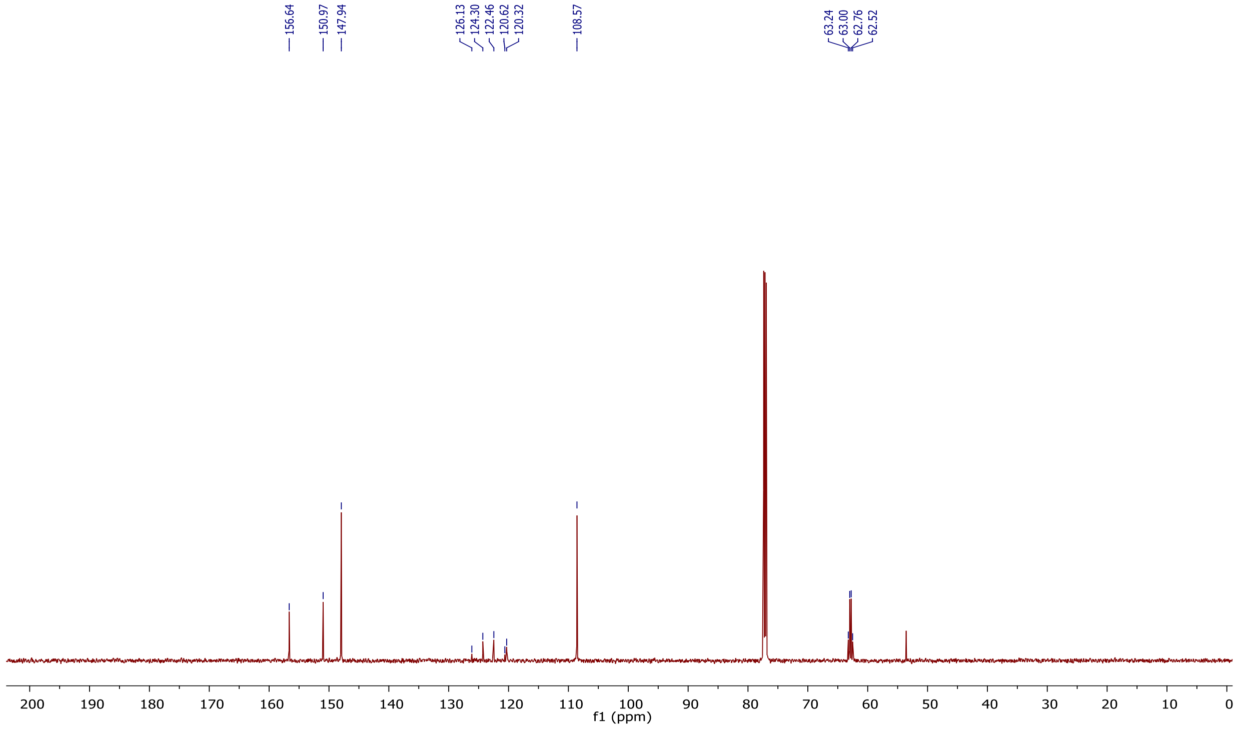
156.64
150.97
147.94
126.13
124.30
122.46
120.62
120.32
108.57
63.24
63.00
62.76
62.52
f1 (ppm)

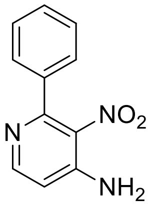
N
NO2
NH2

11

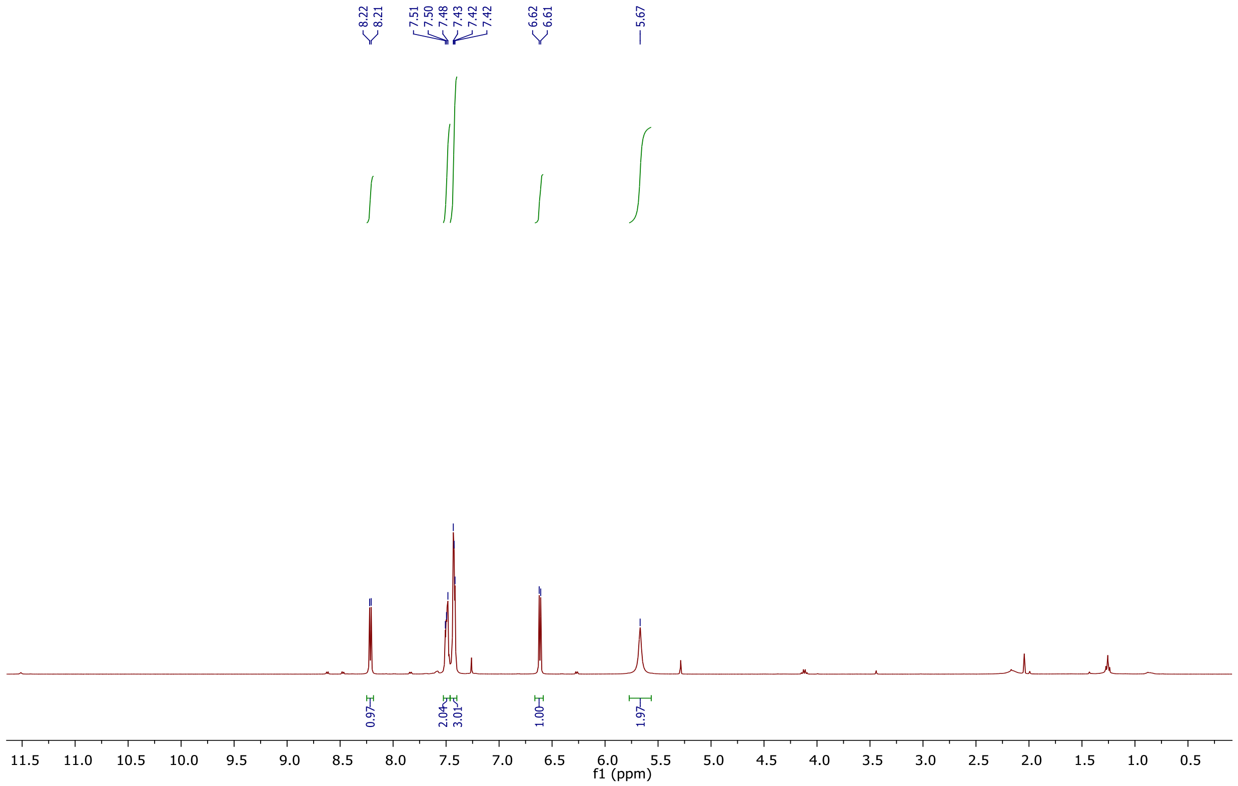
8.22
8.21
7.51
7.50
7.48
7.43
7.42
7.42
6.62
6.61
5.67
0.97
2.04
3.01
1.00
1.97
11.5 11.0 10.5 10.0 9.5 9.0 8.5 8.0 7.5 7.0 6.5 6.0 5.5 5.0 4.5 4.0 3.5 3.0 2.5 2.0 1.5 1.0 0.5
f1 (ppm)

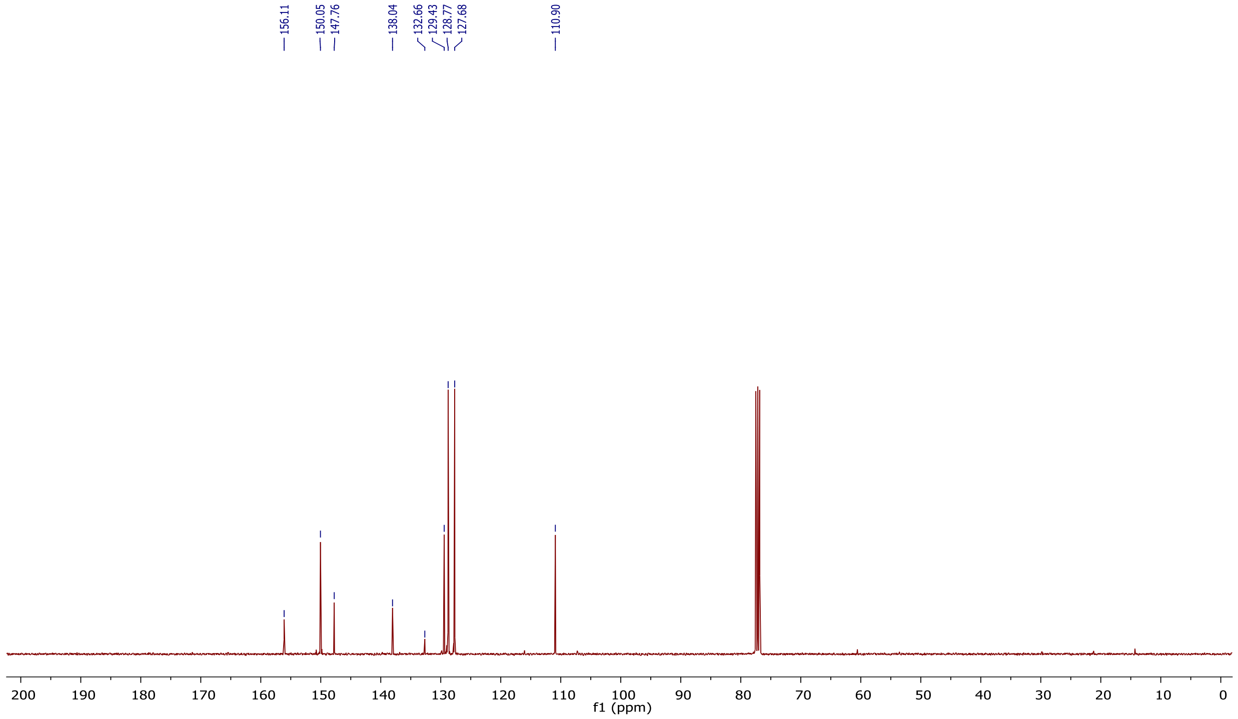
156.11
150.05
147.76
138.04
132.66
129.43
128.77
127.68
110.90
200 190 180 170 160 150 140 130 120 110 100 90 80 70 60 50 40 30 20 10 0
f1 (ppm)

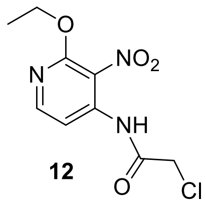

12

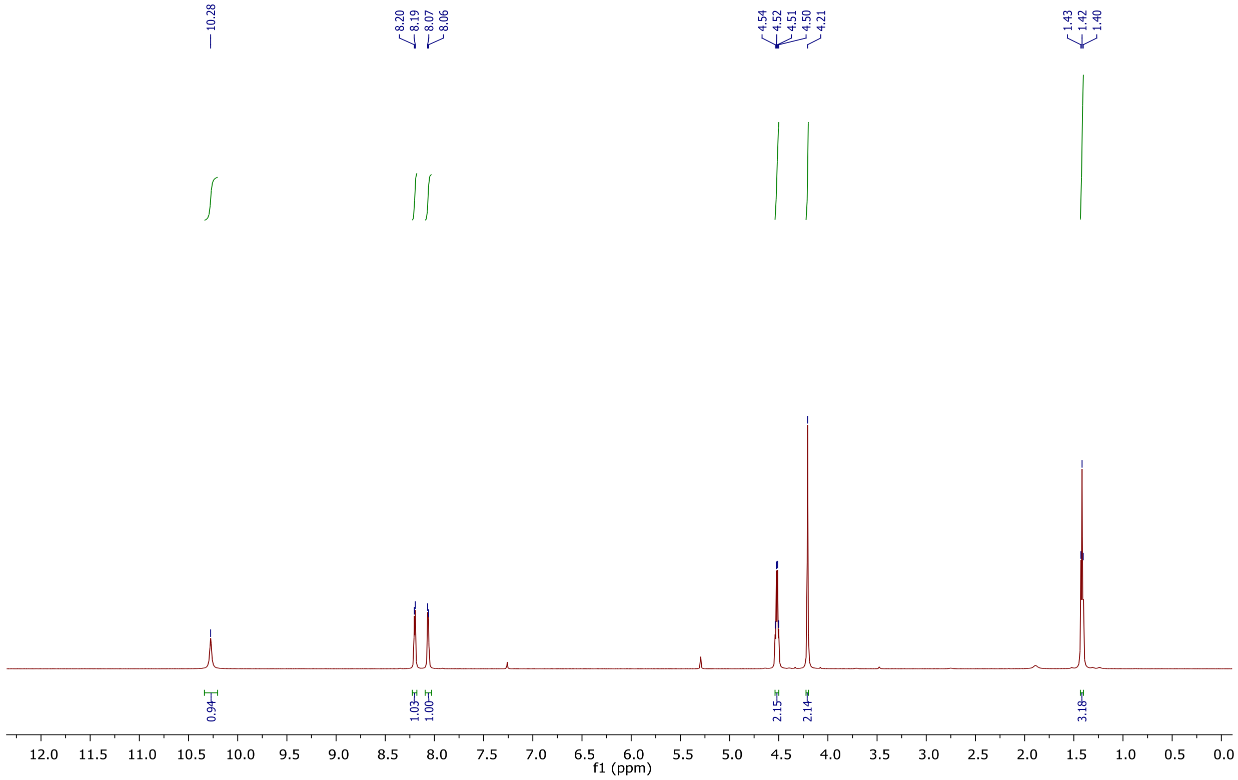
10.28
8.20
8.19
8.07
8.06
4.54
4.52
4.51
4.50
4.21
1.43
1.42
1.40
0.94
1.03
1.00
2.15
2.14
3.18
12.0 11.5 11.0 10.5 10.0 9.5 9.0 8.5 8.0 7.5 7.0 6.5 6.0 5.5 5.0 4.5 4.0 3.5 3.0 2.5 2.0 1.5 1.0 0.5 0.0
f1 (ppm)

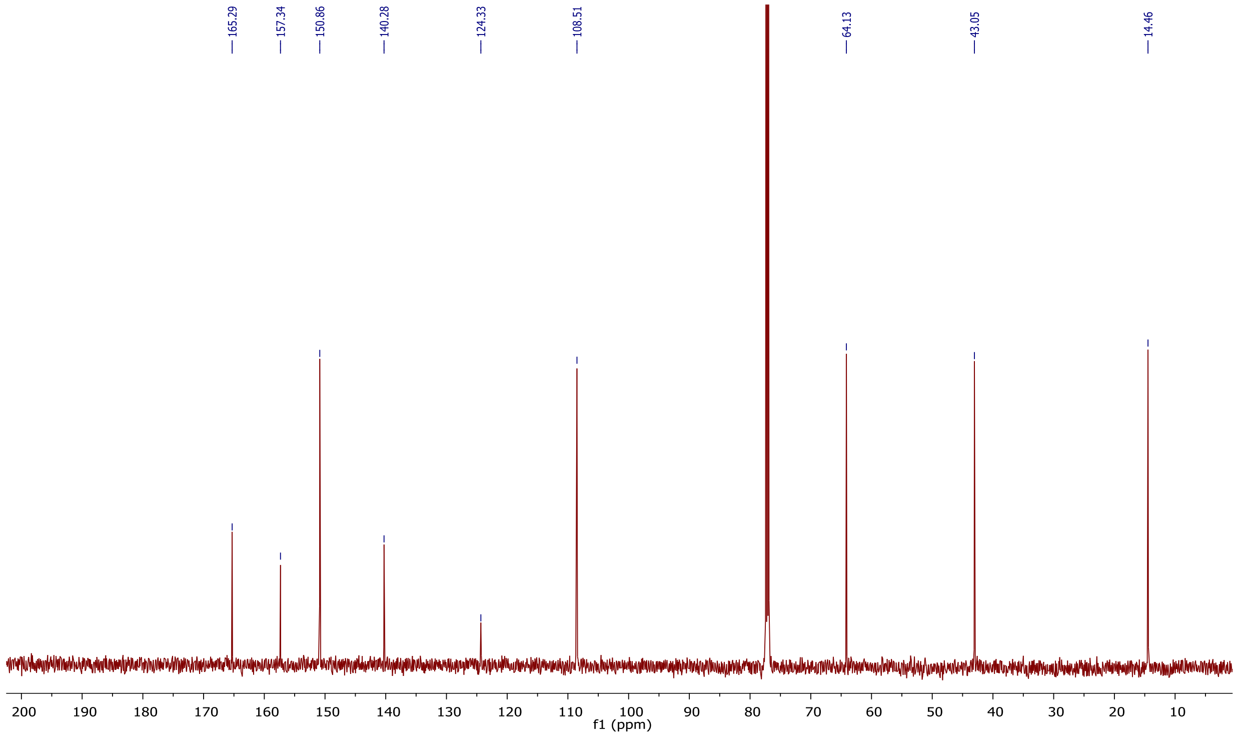
165.29
157.34
150.86
140.28
124.33
108.51
64.13
43.05
14.46
200 190 180 170 160 150 140 130 120 110 100 90 80 70 60 50 40 30 20 10
f1 (ppm)

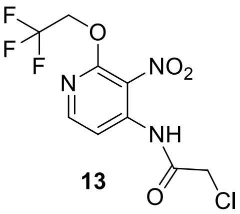
F
F
F
O
N
NO2
NH
O
Cl

13

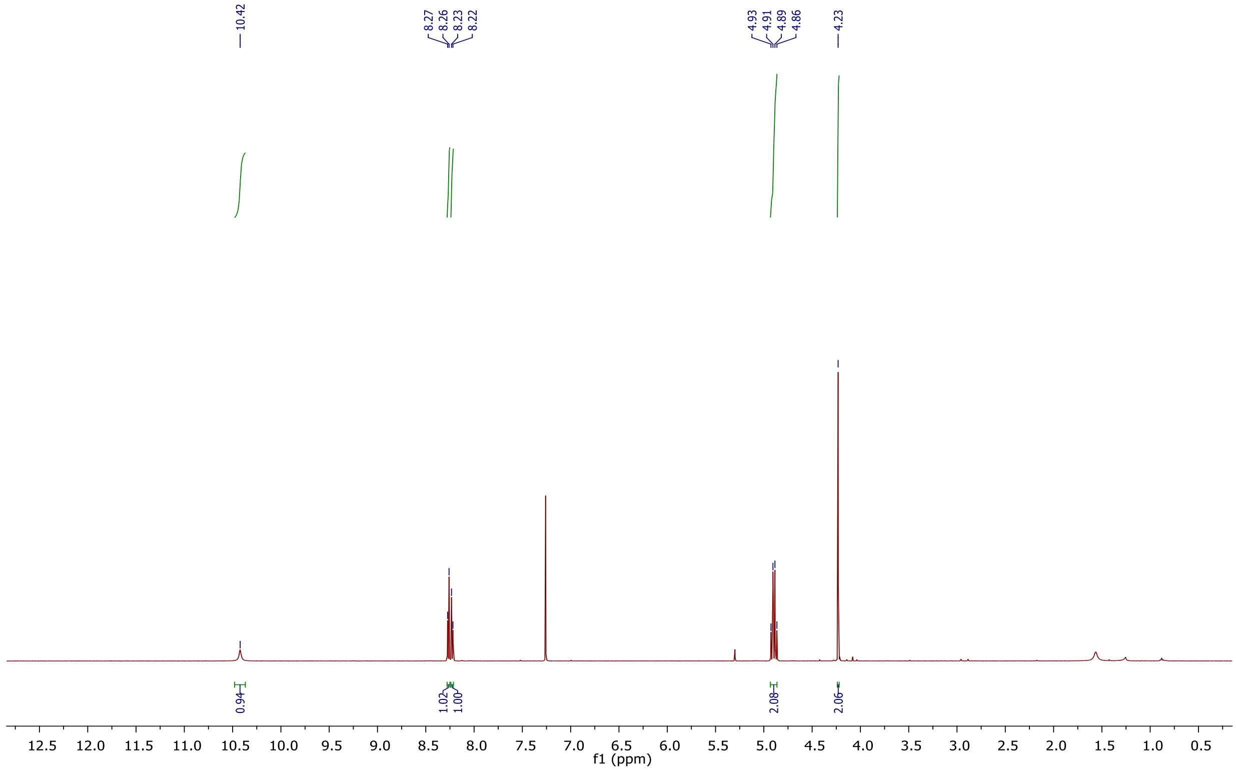
10.42
8.27
8.26
8.23
8.22
4.93
4.91
4.89
4.86
4.23
0.94
1.02
1.00
2.08
2.06
12.5 12.0 11.5 11.0 10.5 10.0 9.5 9.0 8.5 8.0 7.5 7.0 6.5 6.0 5.5 5.0 4.5 4.0 3.5 3.0 2.5 2.0 1.5 1.0 0.5
f1 (ppm)

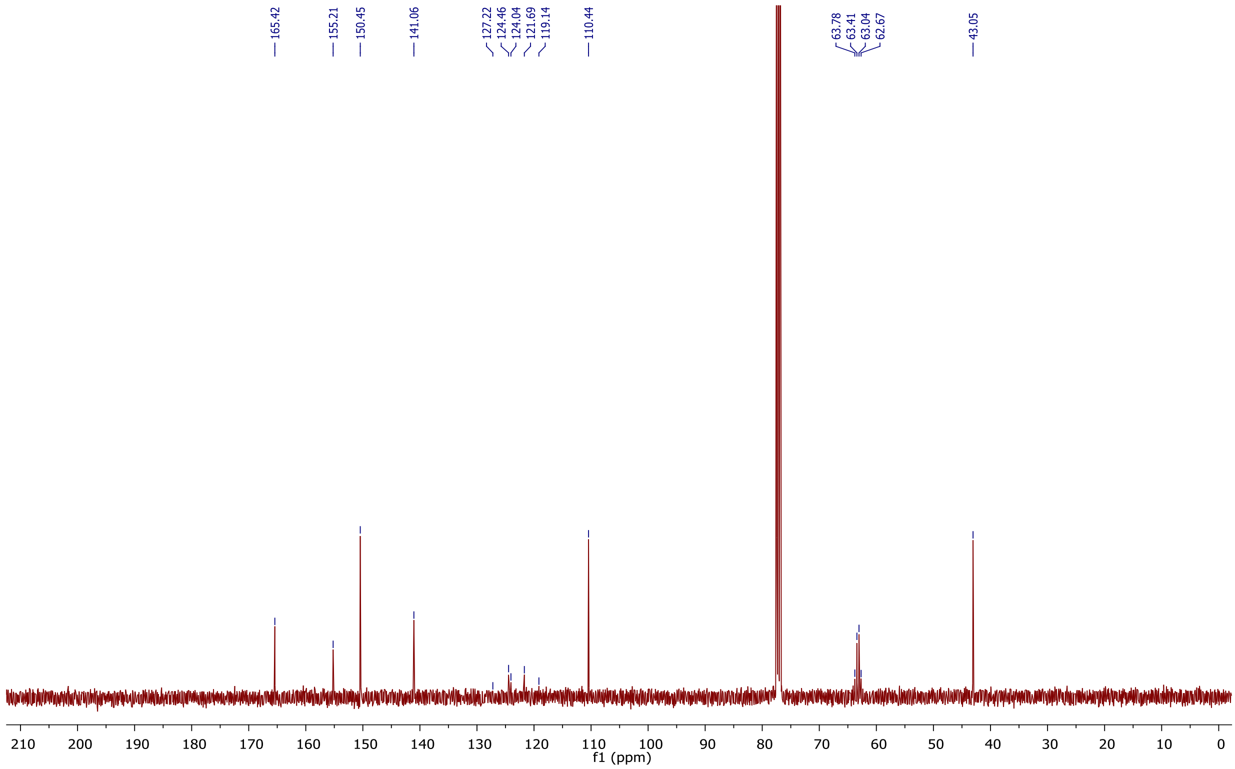
165.42
155.21
150.45
141.06
127.22
124.46
124.04
121.69
119.14
110.44
63.78
63.41
63.04
62.67
43.05
210 200 190 180 170 160 150 140 130 120 110 100 90 80 70 60 50 40 30 20 10 0
f1 (ppm)

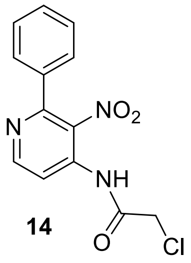

NO2
N
NH
O
Cl

14

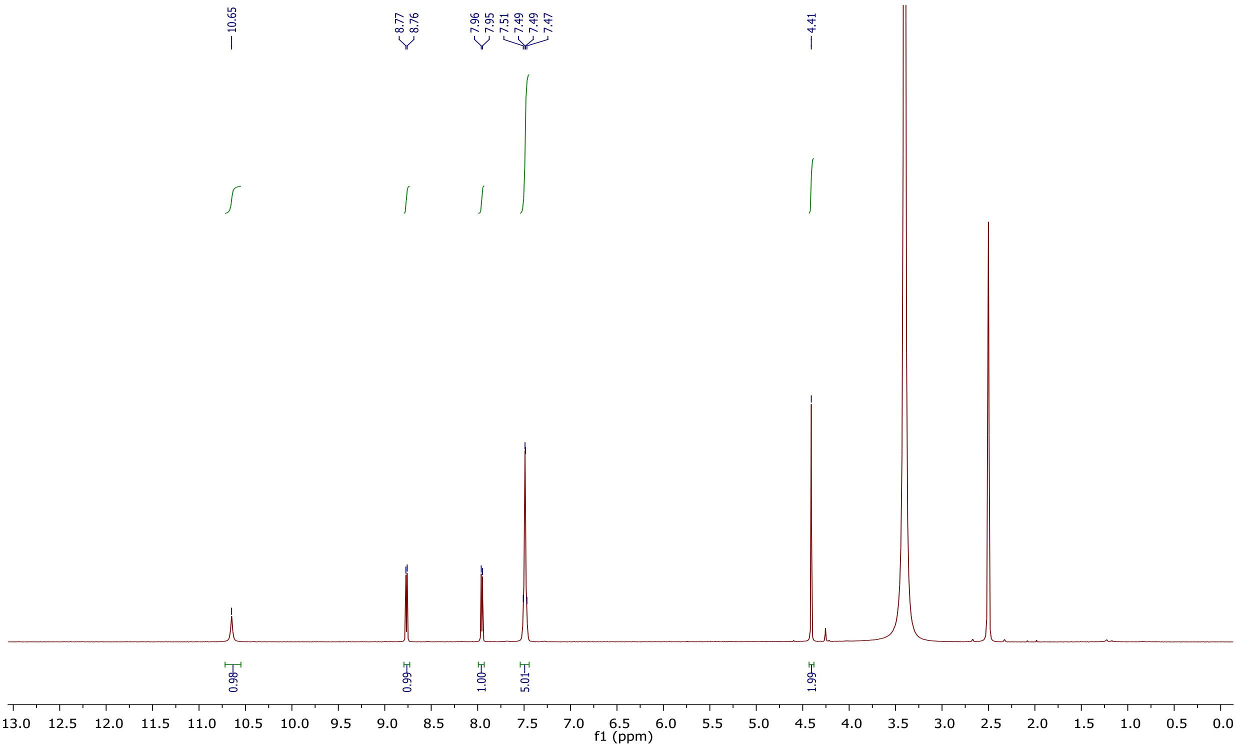

10.65
8.77
8.76
7.96
7.95
7.51
7.49
7.49
7.47
4.41
0.98
0.99
1.00
5.01
1.99
13.0 12.5 12.0 11.5 11.0 10.5 10.0 9.5 9.0 8.5 8.0 7.5 7.0 6.5 6.0 5.5 5.0 4.5 4.0 3.5 3.0 2.5 2.0 1.5 1.0 0.5 0.0
f1 (ppm)

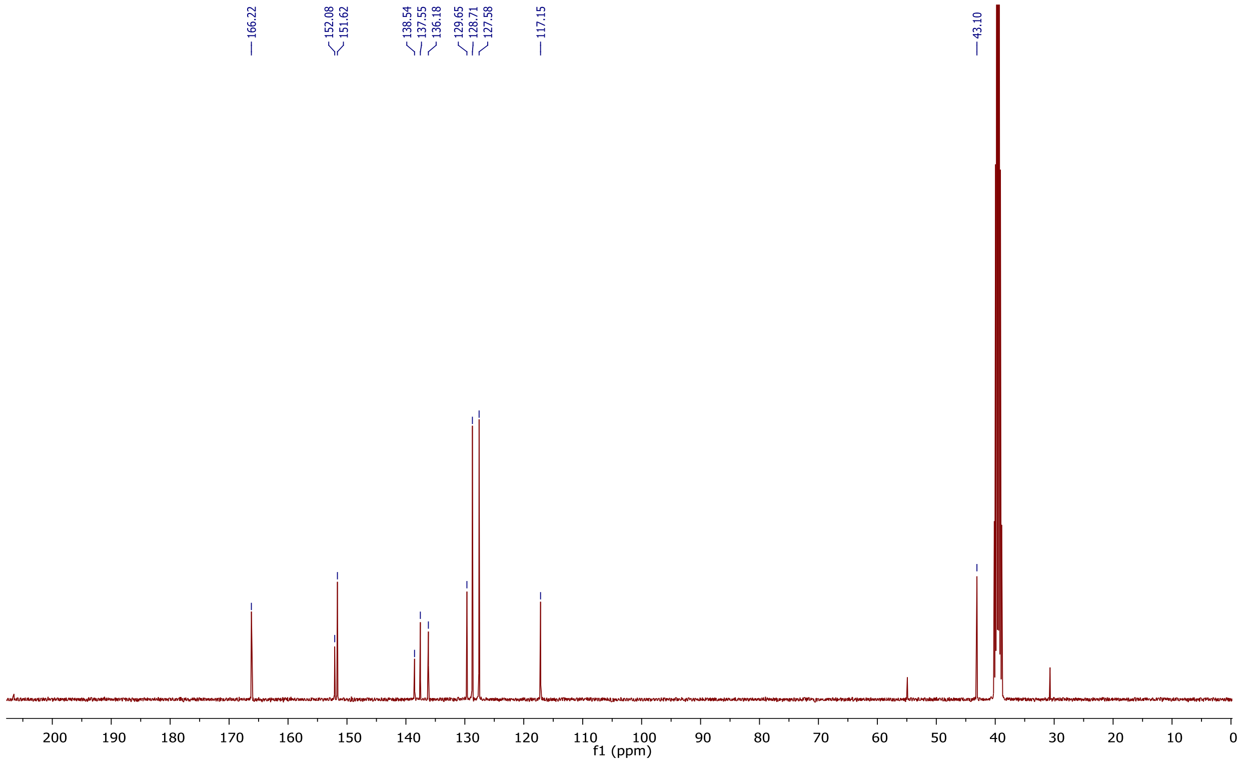

166.22
152.08
151.62
138.54
137.55
136.18
129.65
128.71
127.58
117.15
43.10
200 190 180 170 160 150 140 130 120 110 100 90 80 70 60 50 40 30 20 10 0
f1 (ppm)

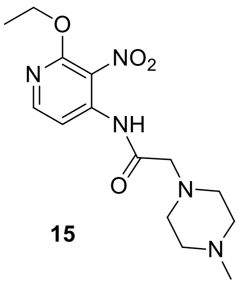
O
NO2
N
NH
O
N
N

15

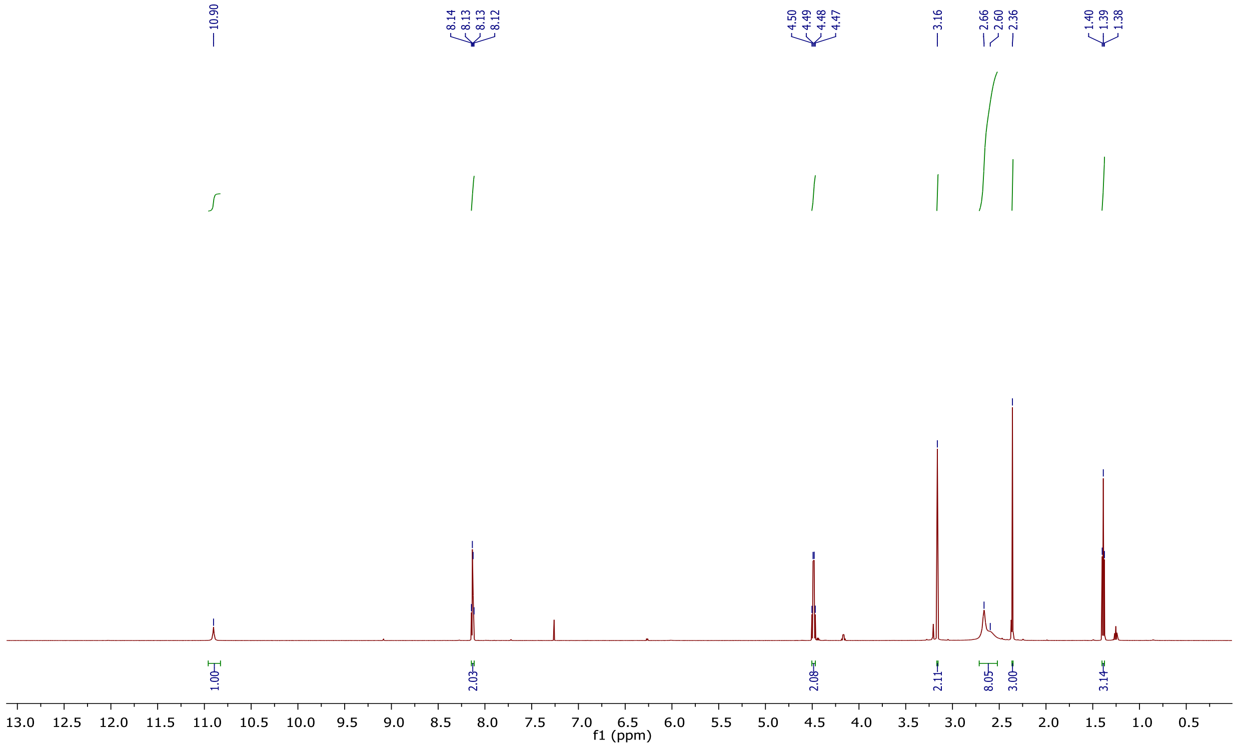
10.90
8.14
8.13
8.13
8.12
4.50
4.49
4.48
4.47
3.16
2.66
2.60
2.36
1.40
1.39
1.38
1.00
2.03
2.08
2.11
8.05
3.00
3.14
13.0 12.5 12.0 11.5 11.0 10.5 10.0 9.5 9.0 8.5 8.0 7.5 7.0 6.5 6.0 5.5 5.0 4.5 4.0 3.5 3.0 2.5 2.0 1.5 1.0 0.5
f1 (ppm)

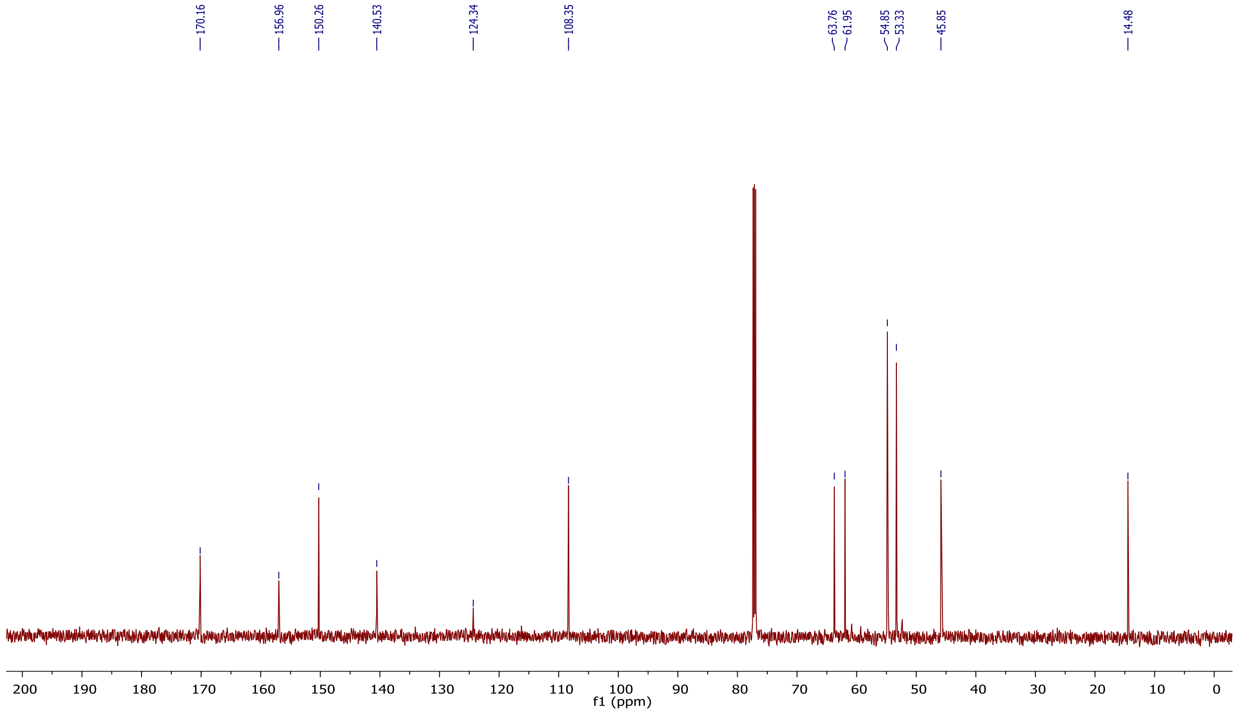
170.16
156.96
150.26
140.53
124.34
108.35
63.76
61.95
54.85
53.33
45.85
14.48
200 190 180 170 160 150 140 130 120 110 100 90 80 70 60 50 40 30 20 10 0
f1 (ppm)

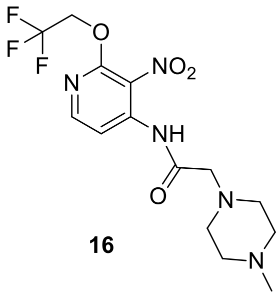
F
F
F
O
NO2
N
NH
O
N
N

16

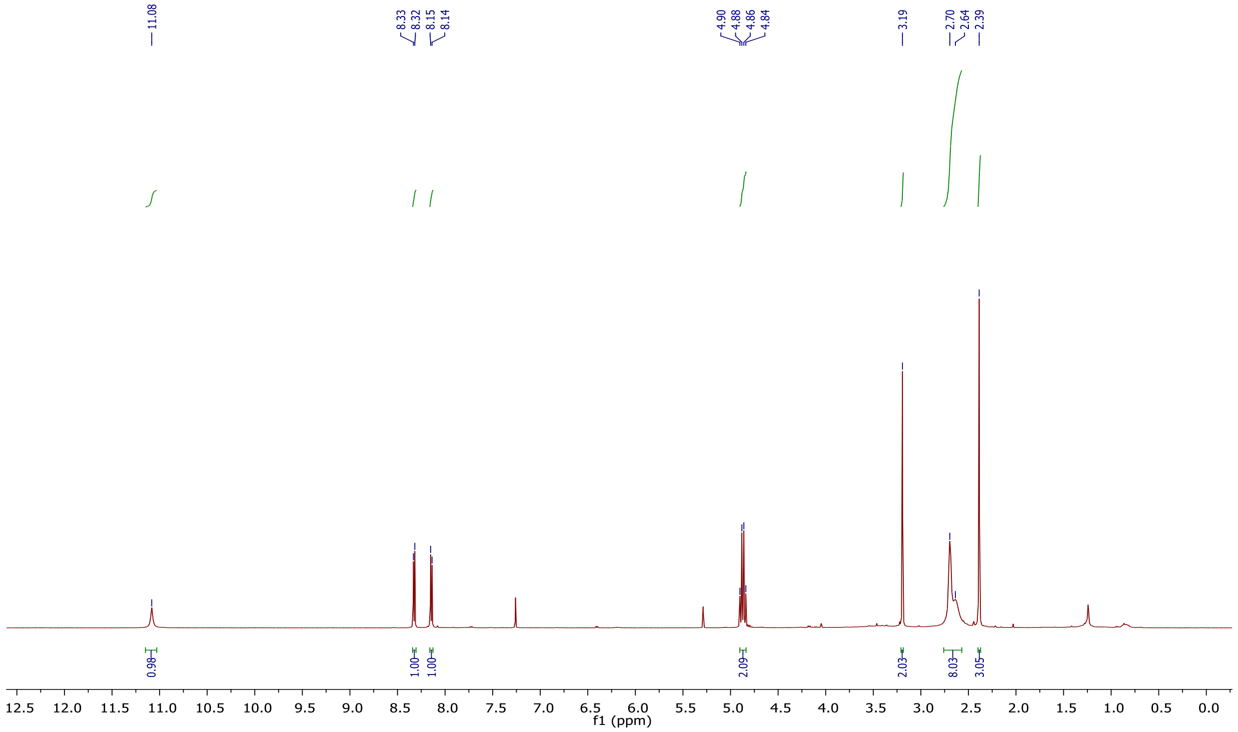
11.08
8.33
8.32
8.15
8.14
4.90
4.88
4.86
4.84
3.19
2.70
2.64
2.39
0.98
1.00
1.00
2.09
2.03
8.03
3.05
12.5 12.0 11.5 11.0 10.5 10.0 9.5 9.0 8.5 8.0 7.5 7.0 6.5 6.0 5.5 5.0 4.5 4.0 3.5 3.0 2.5 2.0 1.5 1.0 0.5 0.0
f1 (ppm)

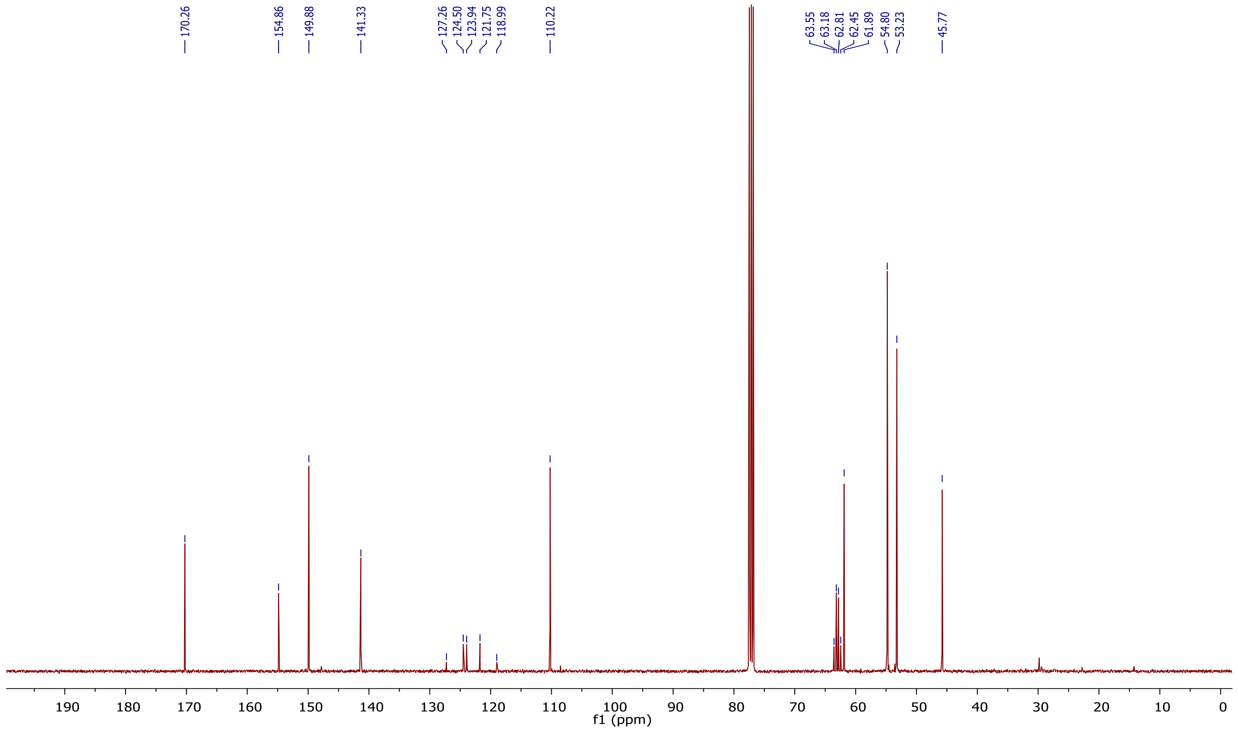
170.26
154.86
149.88
141.33
127.26
124.50
123.94
121.75
118.99
110.22
63.55
63.18
62.81
62.45
61.89
54.80
53.23
45.77
190 180 170 160 150 140 130 120 110 100 90 80 70 60 50 40 30 20 10 0
f1 (ppm)

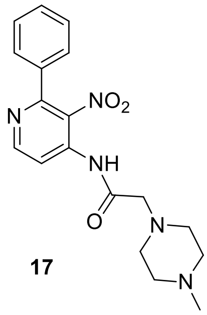

17

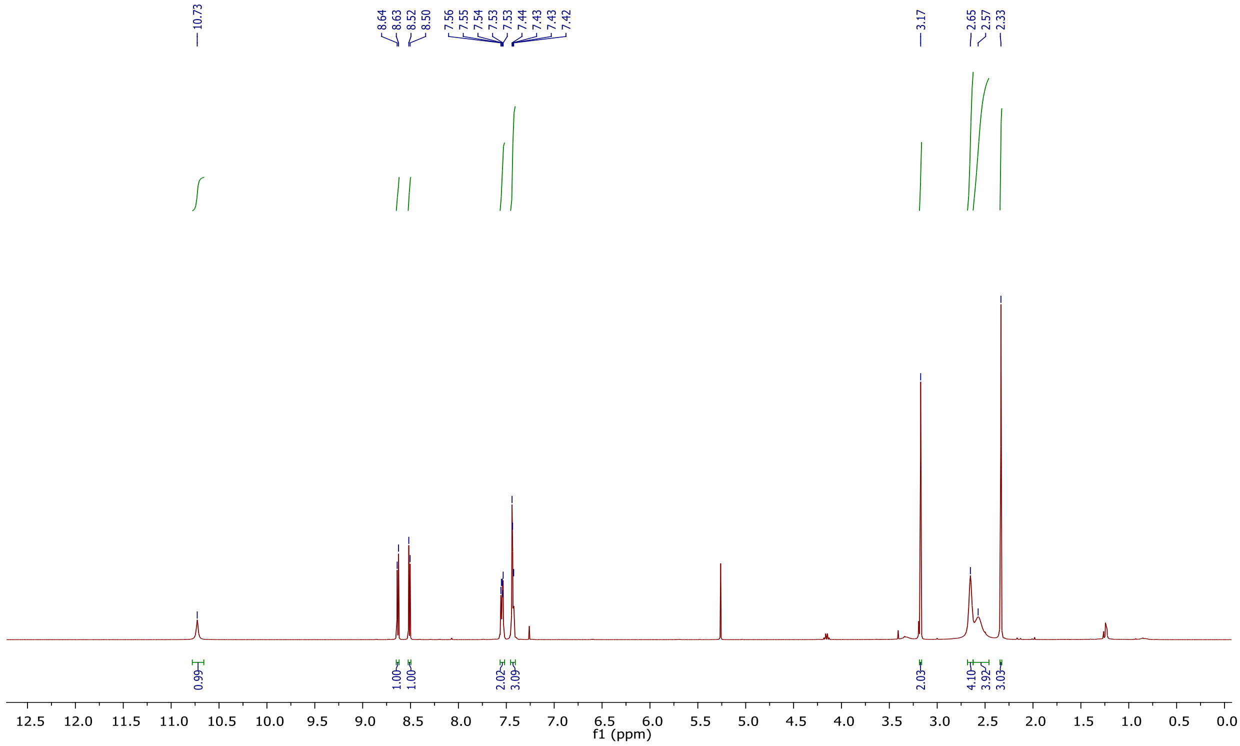
10.73
8.64 8.63 8.52 8.50
7.56 7.55 7.54 7.53 7.53 7.44 7.43 7.43 7.42
3.17
2.65 2.57 2.33
0.99
1.00 1.00
2.02 3.09
2.03
4.10 3.92 3.03
12.5 12.0 11.5 11.0 10.5 10.0 9.5 9.0 8.5 8.0 7.5 7.0 6.5 6.0 5.5 5.0 4.5 4.0 3.5 3.0 2.5 2.0 1.5 1.0 0.5 0.0
f1 (ppm)

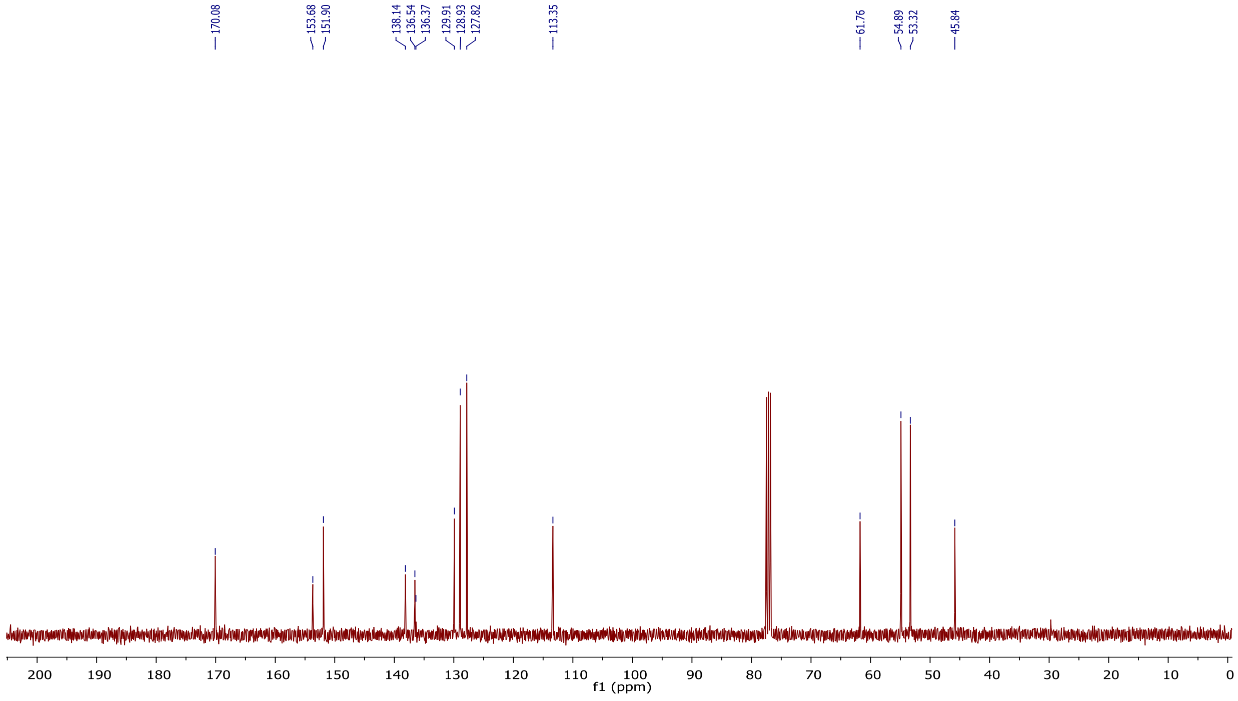
170.08
153.68 151.90
138.14 136.54 136.37 129.91 128.93 127.82
113.35
61.76
54.89 53.32
45.84
200 190 180 170 160 150 140 130 120 110 100 90 80 70 60 50 40 30 20 10 0
f1 (ppm)

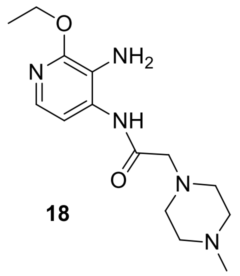
O
NH2
N
NH
O
N
N

18

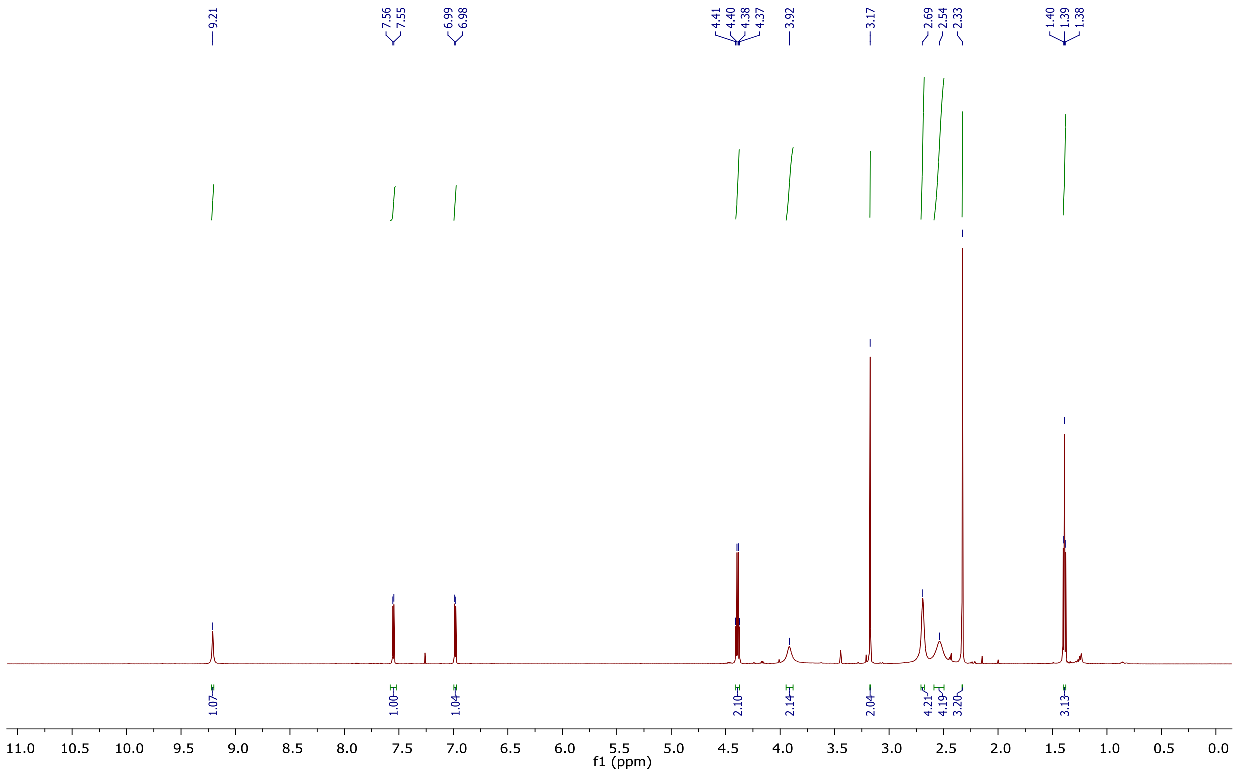
9.21
7.56
7.55
6.99
6.98
4.41
4.40
4.38
4.37
3.92
3.17
2.69
2.54
2.33
1.40
1.39
1.38
1.07
1.00
1.04
2.10
2.14
2.04
4.21
4.19
3.20
3.13
11.0 10.5 10.0 9.5 9.0 8.5 8.0 7.5 7.0 6.5 6.0 5.5 5.0 4.5 4.0 3.5 3.0 2.5 2.0 1.5 1.0 0.5 0.0
f1 (ppm)

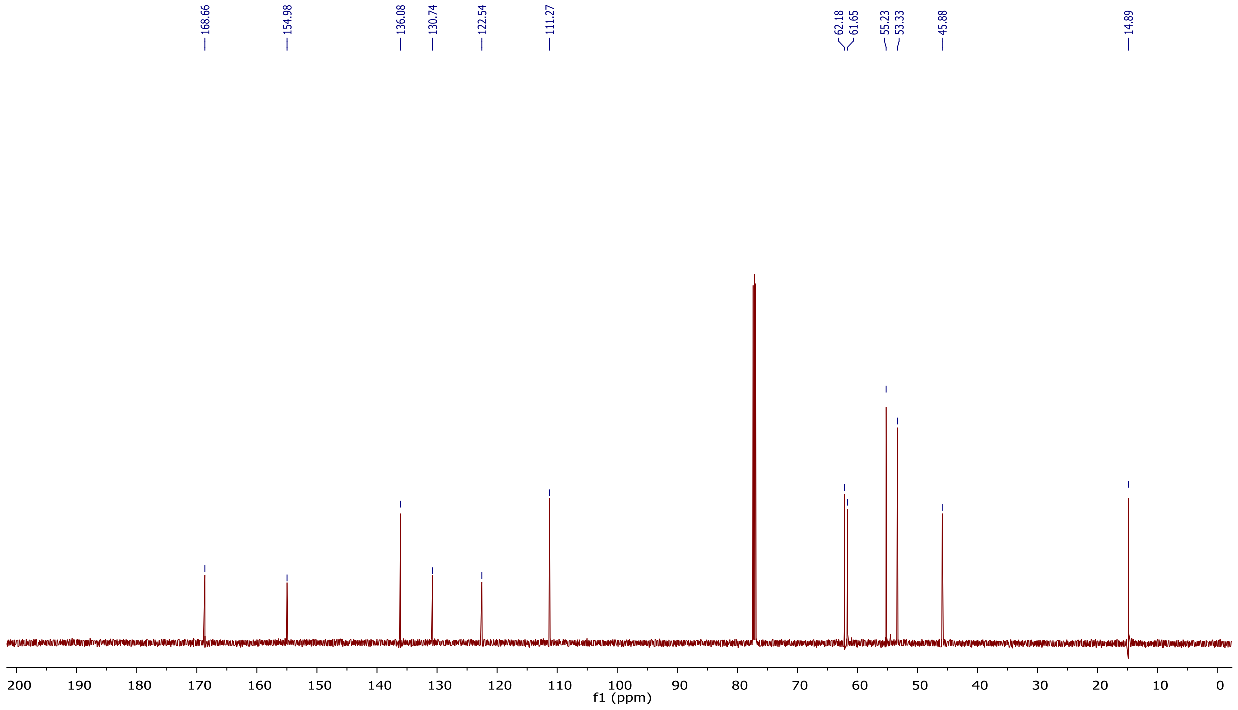
168.66
154.98
136.08
130.74
122.54
111.27
62.18
61.65
55.23
53.33
45.88
14.89
200 190 180 170 160 150 140 130 120 110 100 90 80 70 60 50 40 30 20 10 0
f1 (ppm)

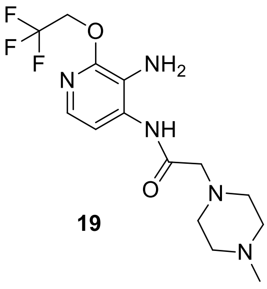
F
F
F
O
NH2
N
NH
O
N
N

19

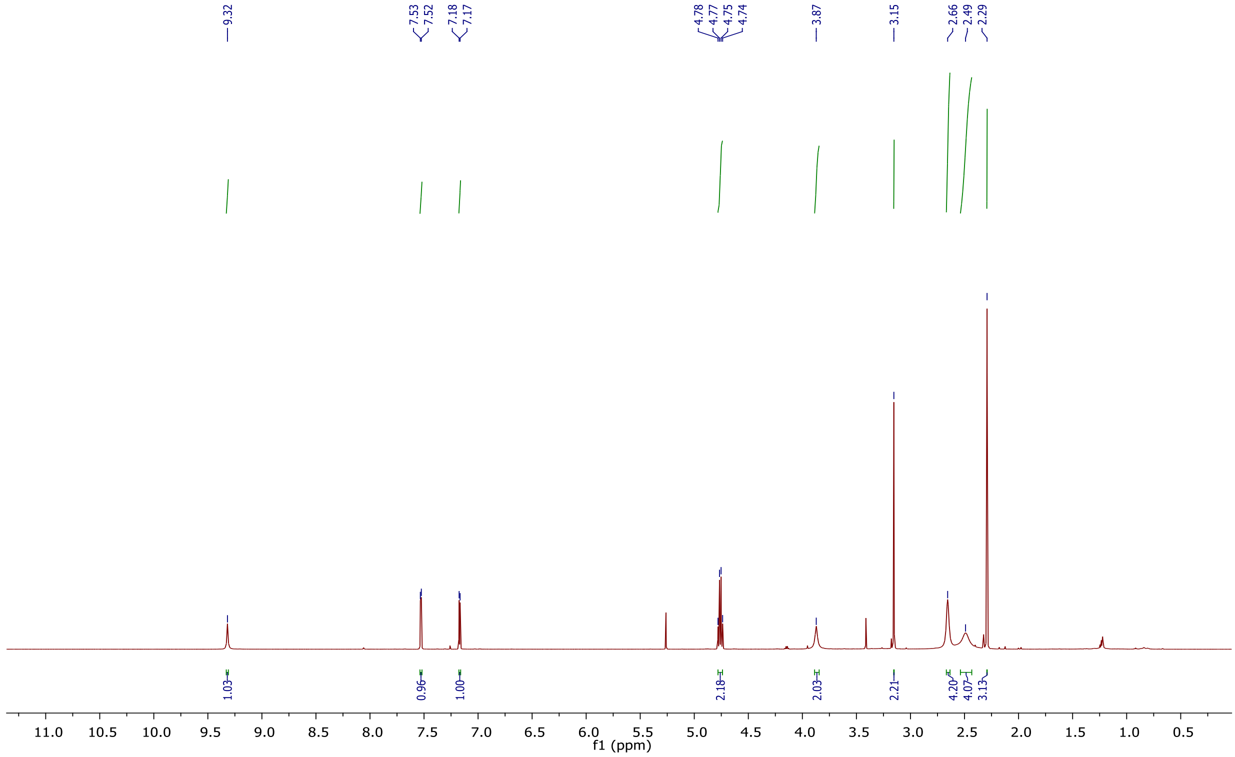
9.32
7.53
7.52
7.18
7.17
4.78
4.77
4.75
4.74
3.87
3.15
2.66
2.49
2.29
1.03
0.96
1.00
2.18
2.03
2.21
4.20
4.07
3.13
f1 (ppm)

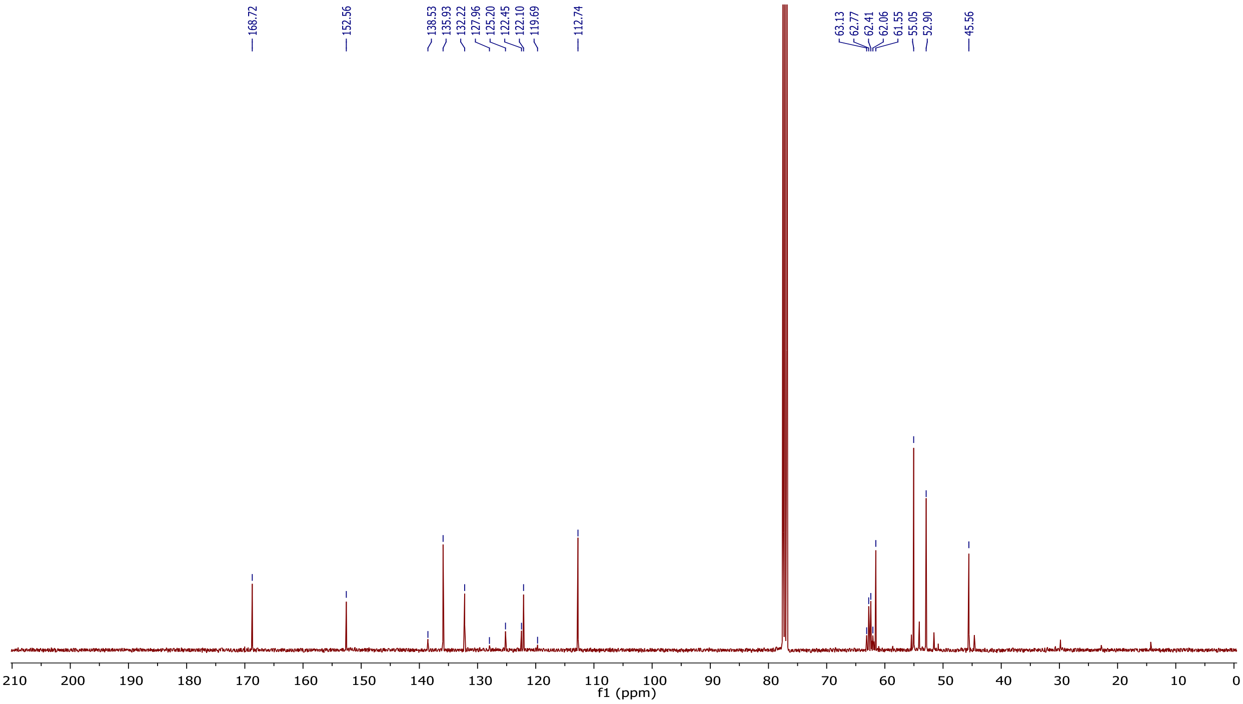
168.72
152.56
138.53
135.93
132.22
127.96
125.20
122.45
122.10
119.69
112.74
63.13
62.77
62.41
62.06
61.55
55.05
52.90
45.56
f1 (ppm)

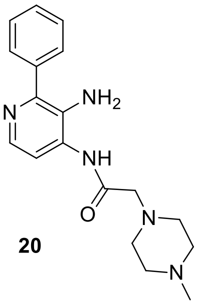
NH2
N
NH
O
N
N

20

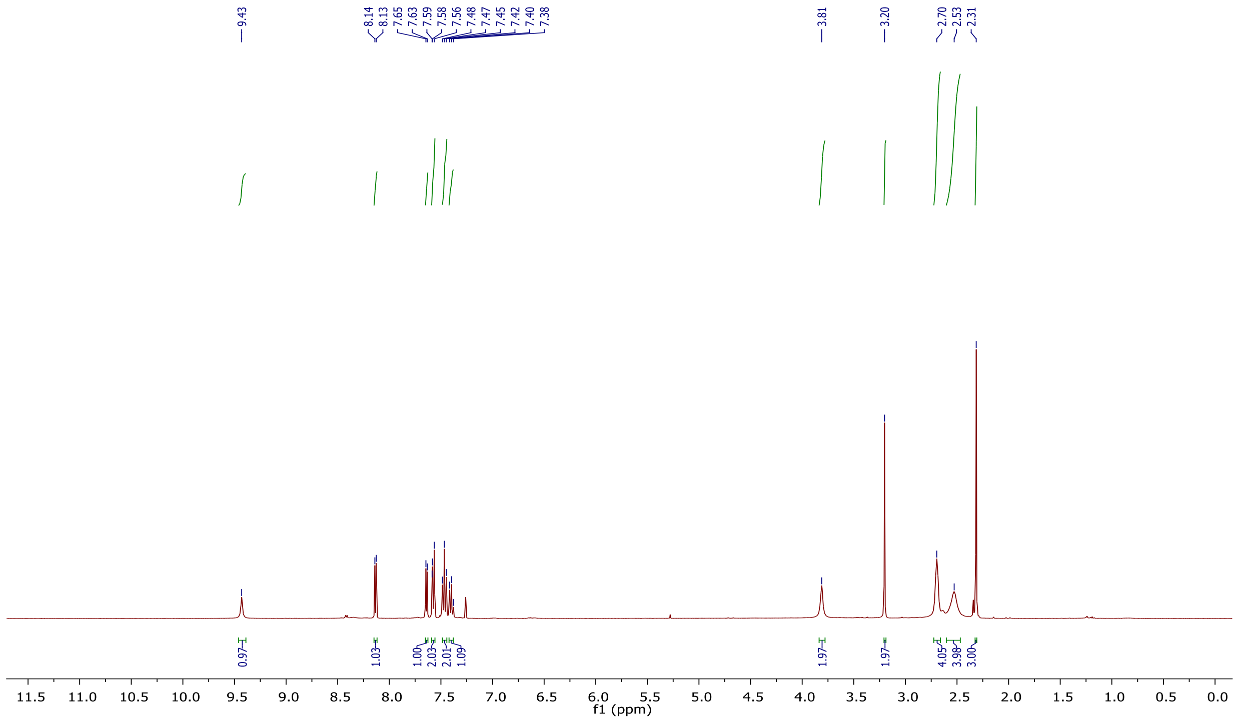
9.43
8.14
8.13
7.65
7.63
7.59
7.58
7.56
7.48
7.47
7.45
7.42
7.40
7.38
3.81
3.20
2.70
2.53
2.31
0.97
1.03
1.00
2.03
2.01
1.09
1.97
1.97
4.05
3.98
3.00
f1 (ppm)

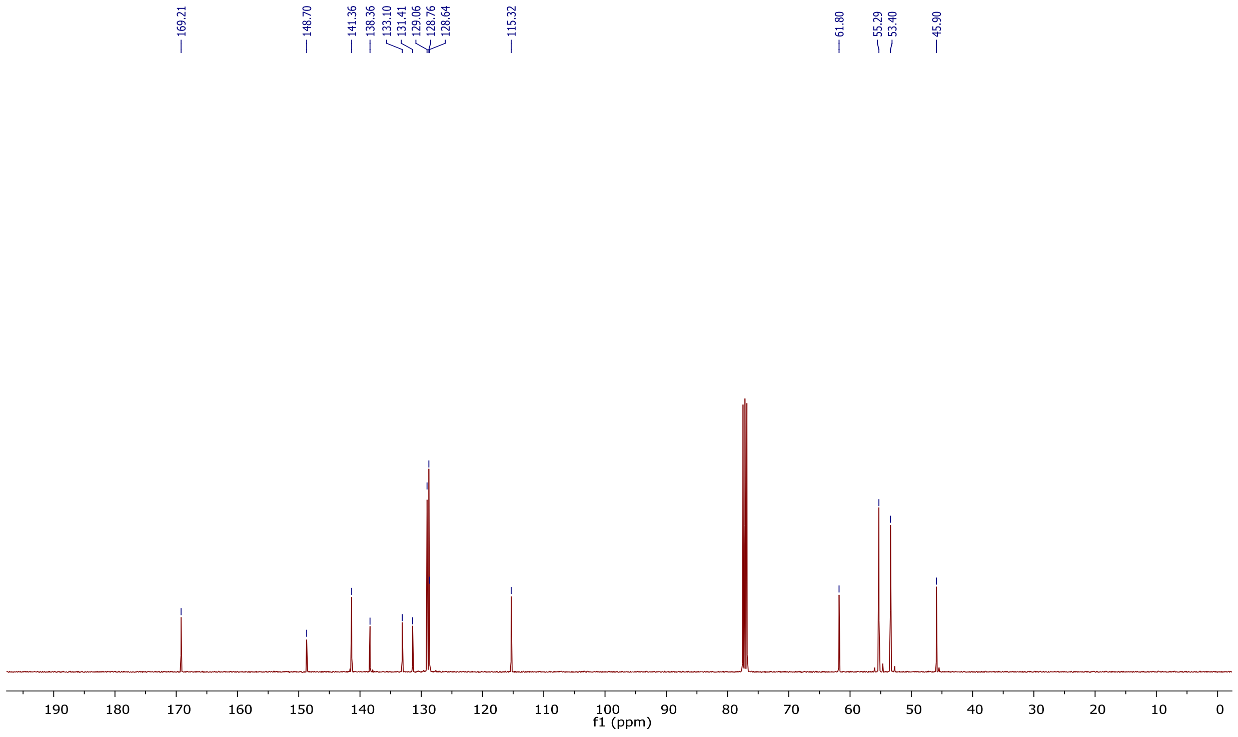
169.21
148.70
141.36
138.36
133.10
131.41
129.06
128.76
128.64
115.32
61.80
55.29
53.40
45.90
f1 (ppm)

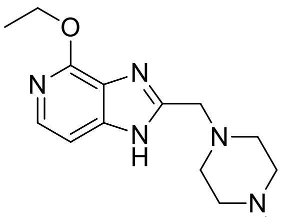

21

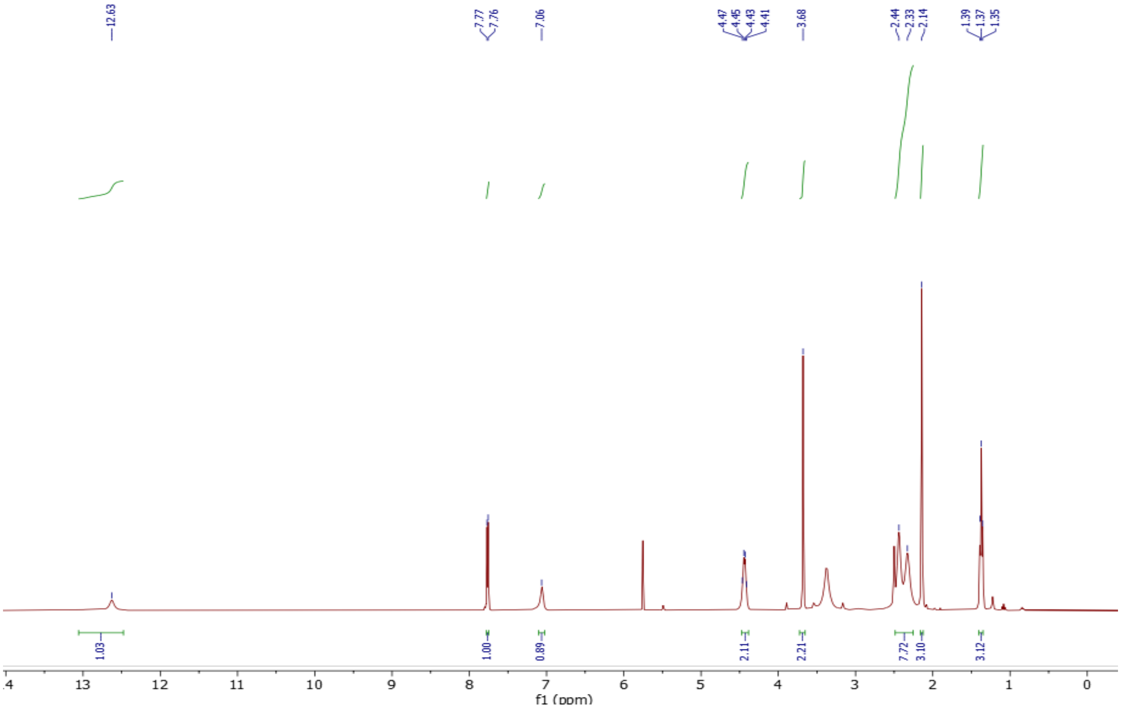
12.63
7.77
7.76
7.06
4.47
4.45
4.43
4.41
3.68
2.44
2.33
2.14
1.39
1.37
1.35
1.03
1.00
0.89
2.11
2.21
7.72
3.10
3.12
f1 (ppm)

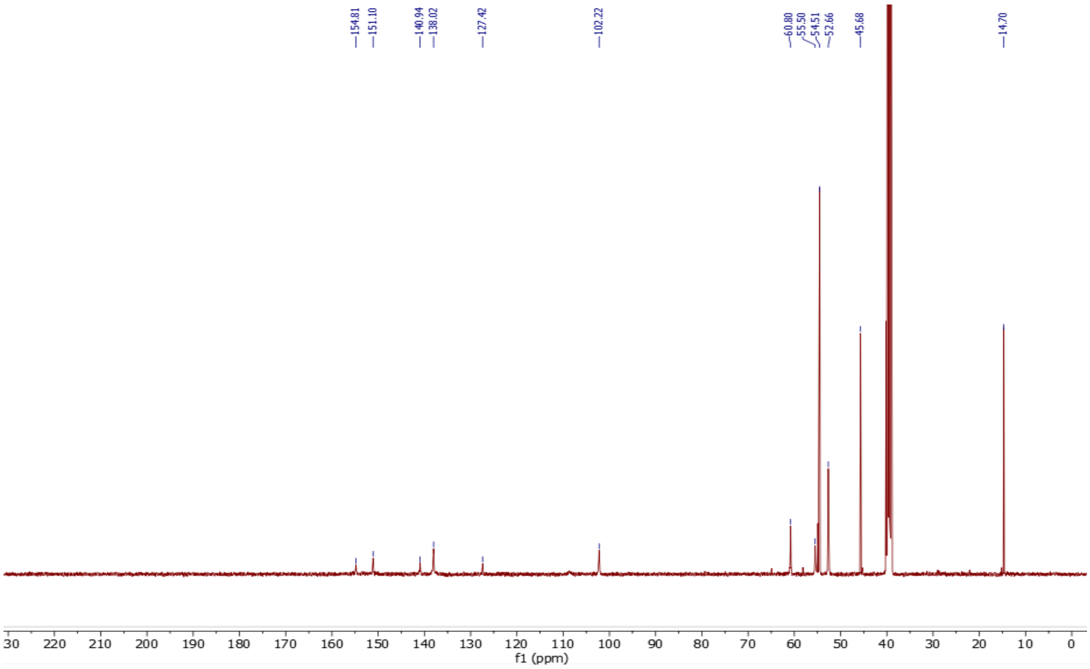
154.81
151.10
140.94
138.02
127.42
102.22
60.80
55.50
54.51
52.66
45.68
14.70
f1 (ppm)

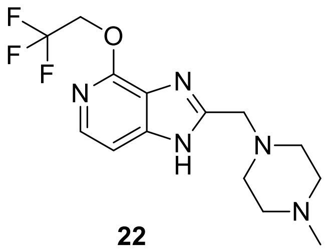
F
F
F
O
N
N
N
H
N
N

22

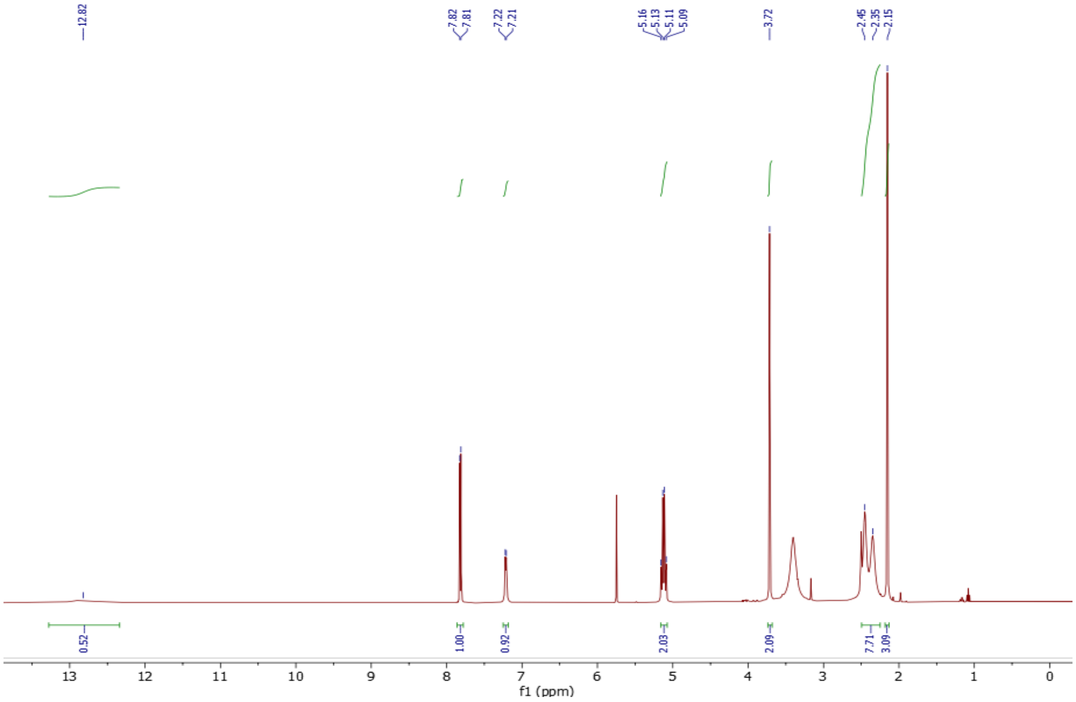
12.82
7.82
7.81
7.22
7.21
5.16
5.13
5.11
5.09
3.72
2.45
2.35
2.15
0.52
1.00
0.92
2.03
2.09
7.71
3.09
f1 (ppm)

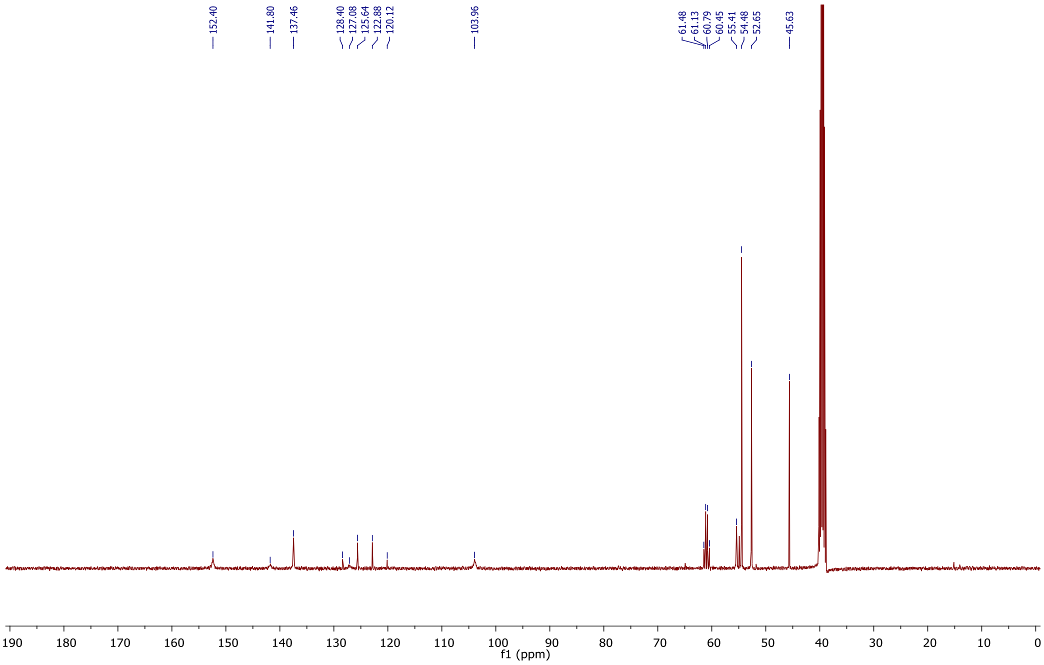
152.40
141.80
137.46
128.40
127.08
125.64
122.88
120.12
103.96
61.48
61.13
60.79
60.45
55.41
54.48
52.65
45.63
f1 (ppm)

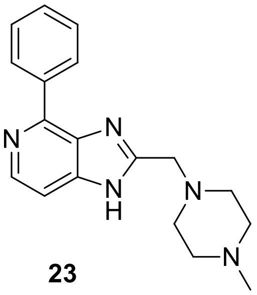
N
N
N
H
N
N

23

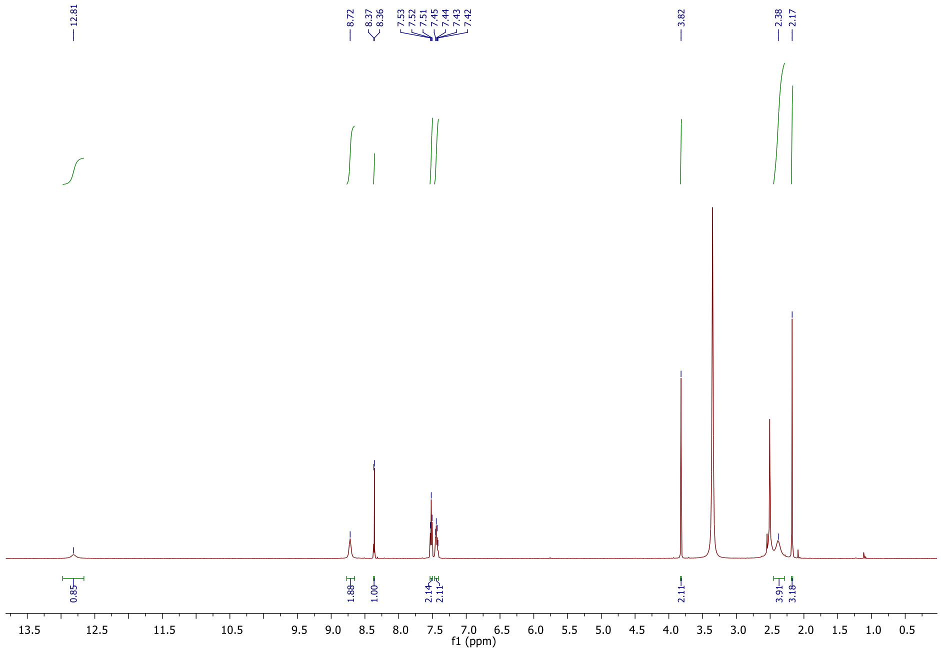
12.81
8.72
8.37
8.36
7.53
7.52
7.51
7.45
7.44
7.43
7.42
3.82
2.38
2.17
0.85
1.88
1.00
2.14
2.11
2.11
3.91
3.18
13.5 12.5 11.5 10.5 9.5 9.0 8.5 8.0 7.5 7.0 6.5 6.0 5.5 5.0 4.5 4.0 3.5 3.0 2.5 2.0 1.5 1.0 0.5
f1 (ppm)

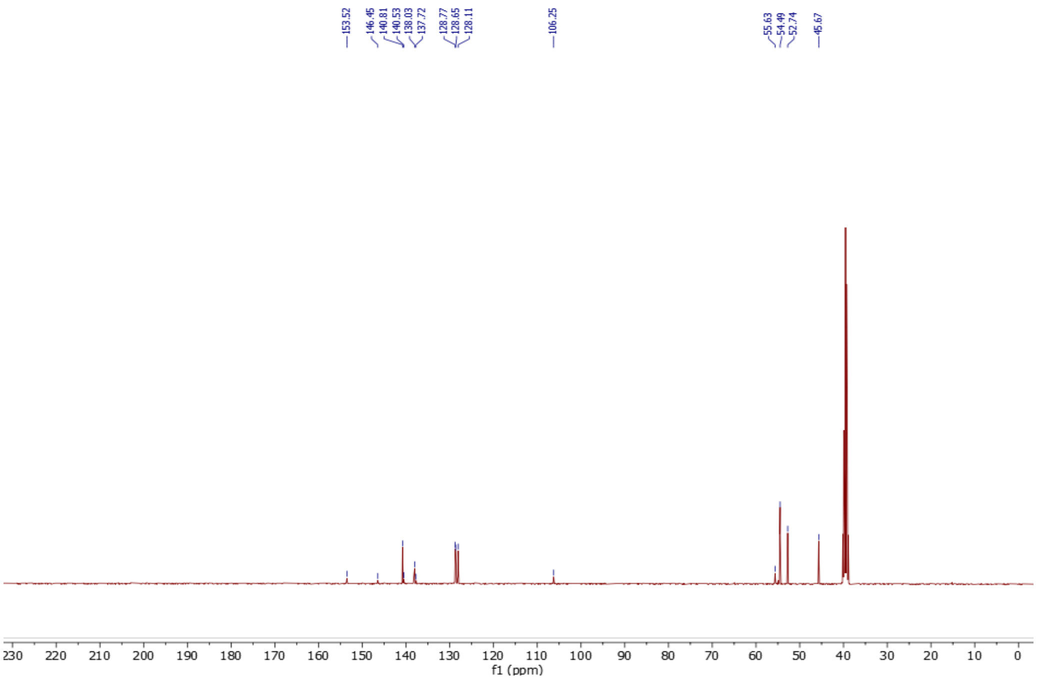
153.52
146.45
140.81
140.53
138.03
137.72
128.77
128.65
128.11
106.25
55.63
54.49
52.74
45.67
230 220 210 200 190 180 170 160 150 140 130 120 110 100 90 80 70 60 50 40 30 20 10 0
f1 (ppm)

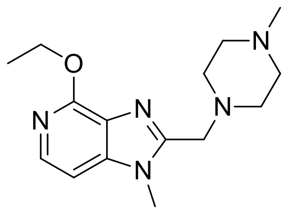
O
N
N
N
N
N

24a

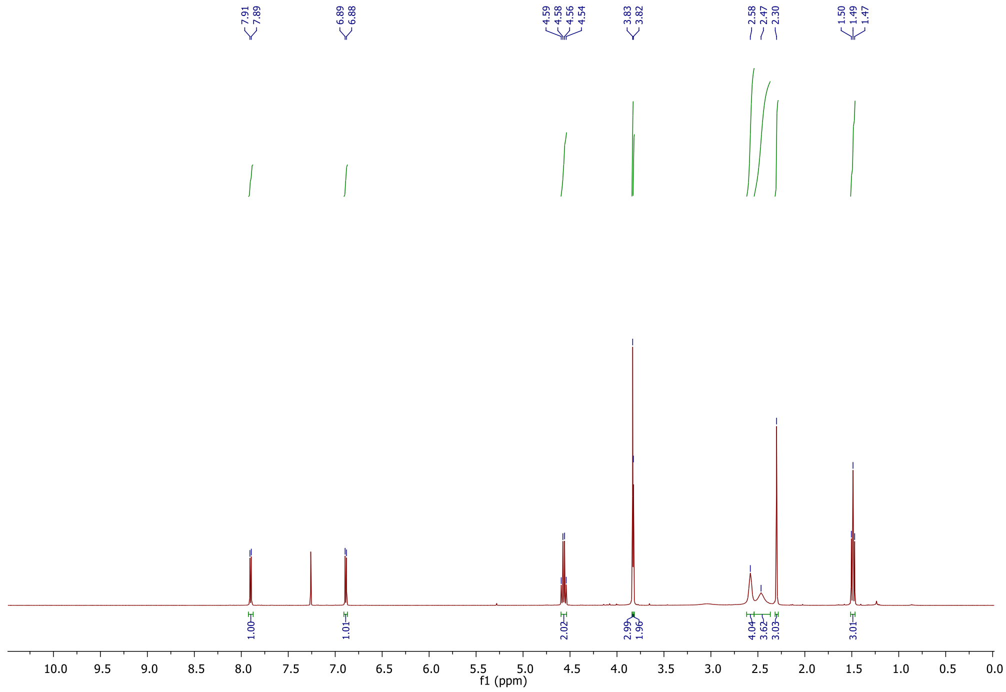
7.91
7.89
6.89
6.88
4.59
4.58
4.56
4.54
3.83
3.82
2.58
2.47
2.30
1.50
1.49
1.47
1.00
1.01
2.02
2.99
1.96
4.04
3.62
3.03
3.01
10.0 9.5 9.0 8.5 8.0 7.5 7.0 6.5 6.0 5.5 5.0 4.5 4.0 3.5 3.0 2.5 2.0 1.5 1.0 0.5 0.0
f1 (ppm)

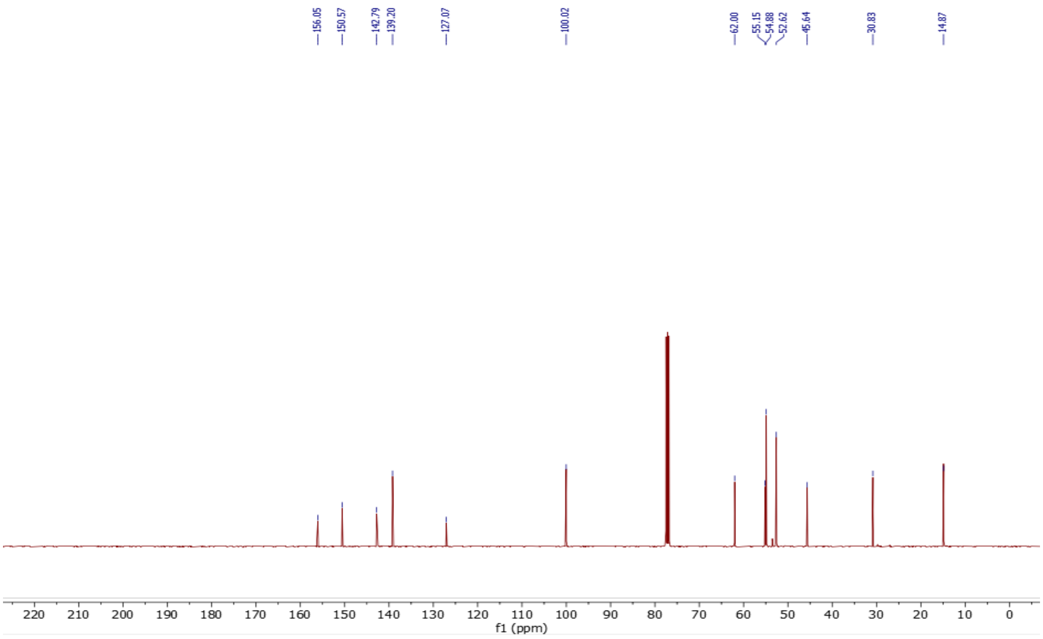
156.05
150.57
142.79
139.20
127.07
100.02
62.00
55.15
54.88
52.62
45.64
30.83
14.87
220 210 200 190 180 170 160 150 140 130 120 110 100 90 80 70 60 50 40 30 20 10 0
f1 (ppm)

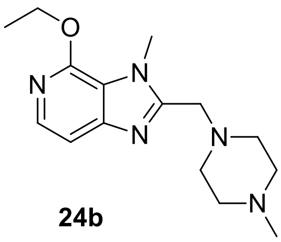

24b

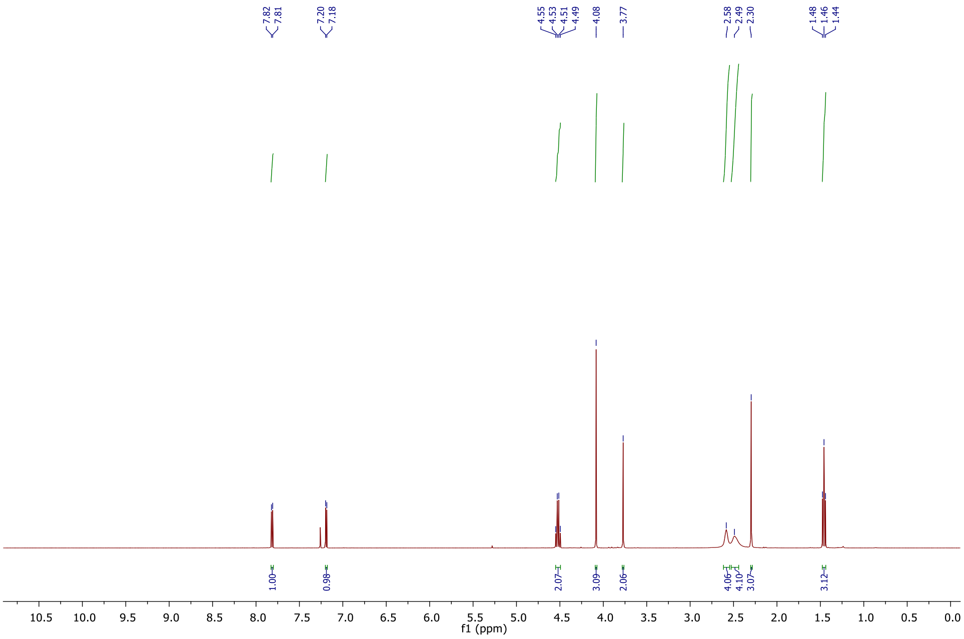
7.82
7.81
7.20
7.18
4.55
4.53
4.51
4.49
4.08
3.77
2.58
2.49
2.30
1.48
1.46
1.44
1.00
0.98
2.07
3.09
2.06
4.06
4.10
3.07
3.12
f1 (ppm)

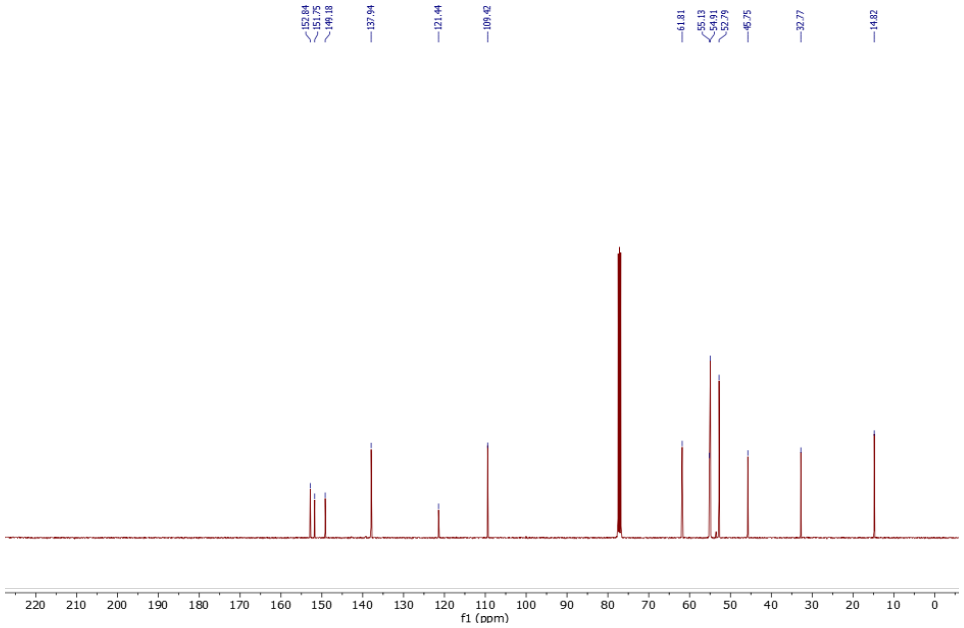
152.84
151.75
149.18
137.94
121.44
109.42
61.81
55.13
54.91
52.79
45.75
32.77
14.82
f1 (ppm)

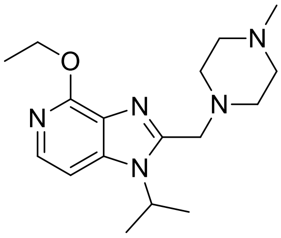

25a

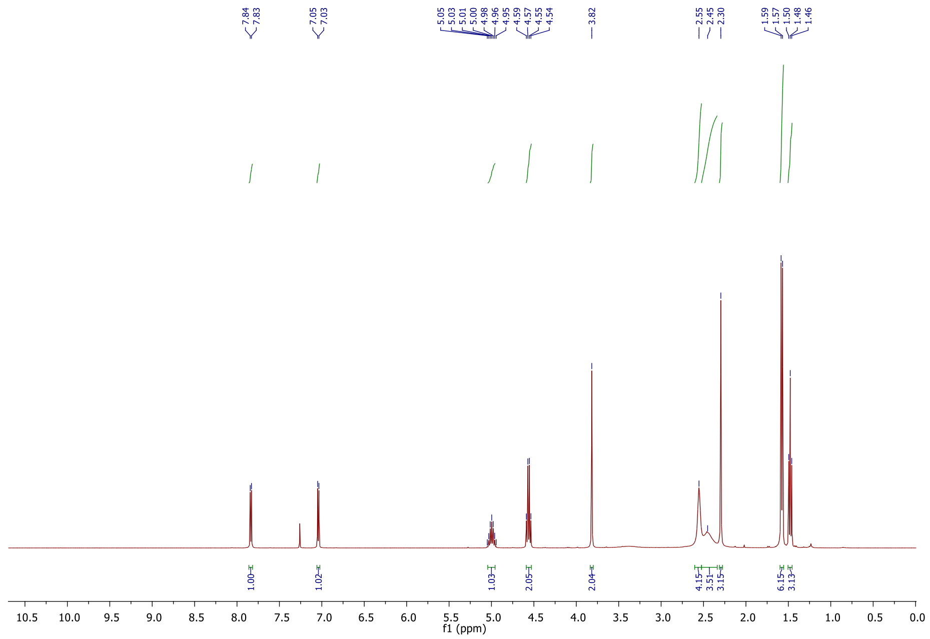

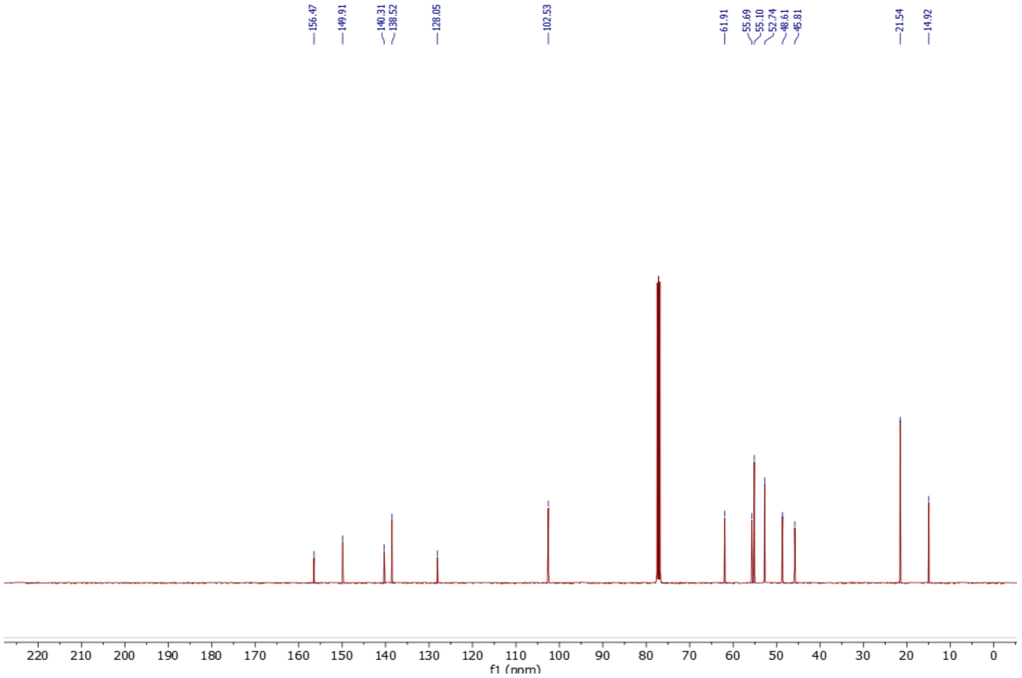

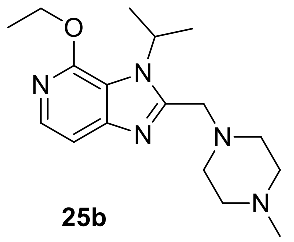

25b

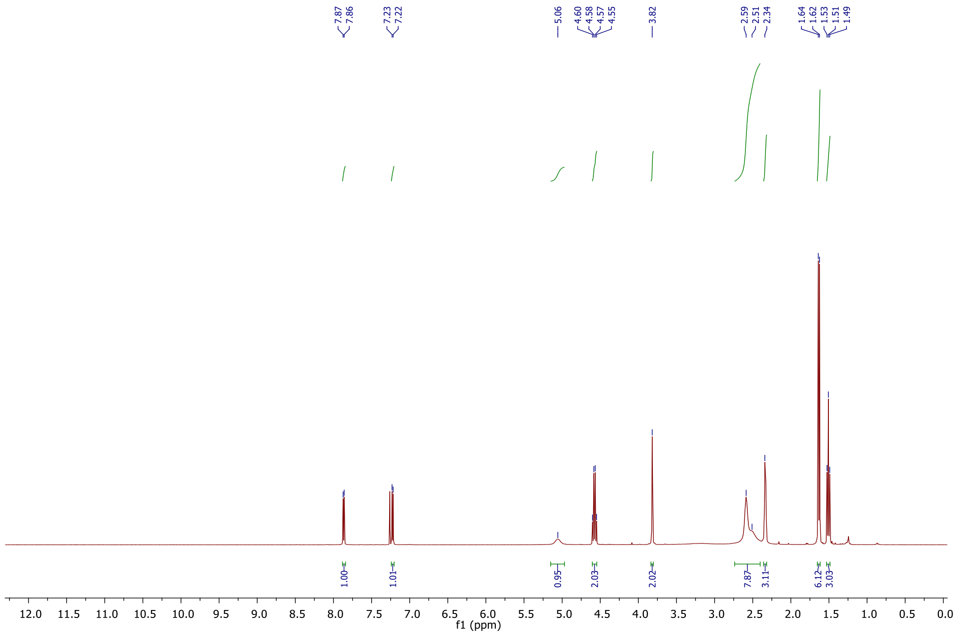
7.87
7.86
7.23
7.22
5.06
4.60
4.58
4.57
4.55
3.82
2.59
2.51
2.34
1.64
1.62
1.53
1.51
1.49
1.00
1.01
0.95
2.03
2.02
7.87
3.11
6.12
3.03
12.0 11.5 11.0 10.5 10.0 9.5 9.0 8.5 8.0 7.5 7.0 6.5 6.0 5.5 5.0 4.5 4.0 3.5 3.0 2.5 2.0 1.5 1.0 0.5 0.0
f1 (ppm)

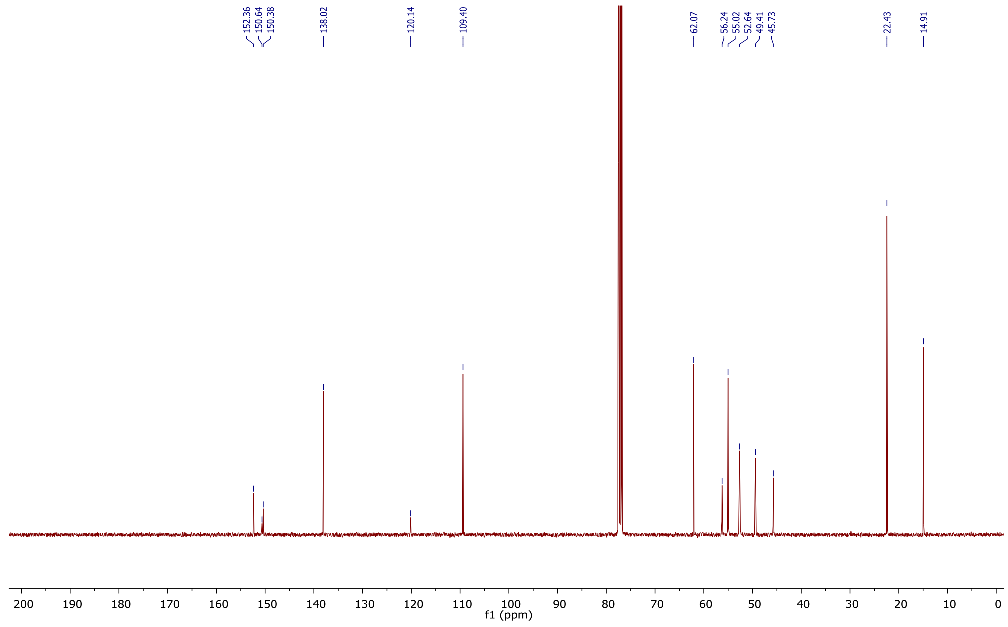
152.36
150.64
150.38
138.02
120.14
109.40
62.07
56.24
55.02
52.64
49.41
45.73
22.43
14.91
200 190 180 170 160 150 140 130 120 110 100 90 80 70 60 50 40 30 20 10 0
f1 (ppm)

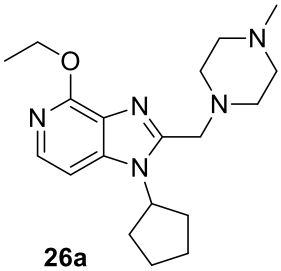
O
N
N
N
N
N

26a

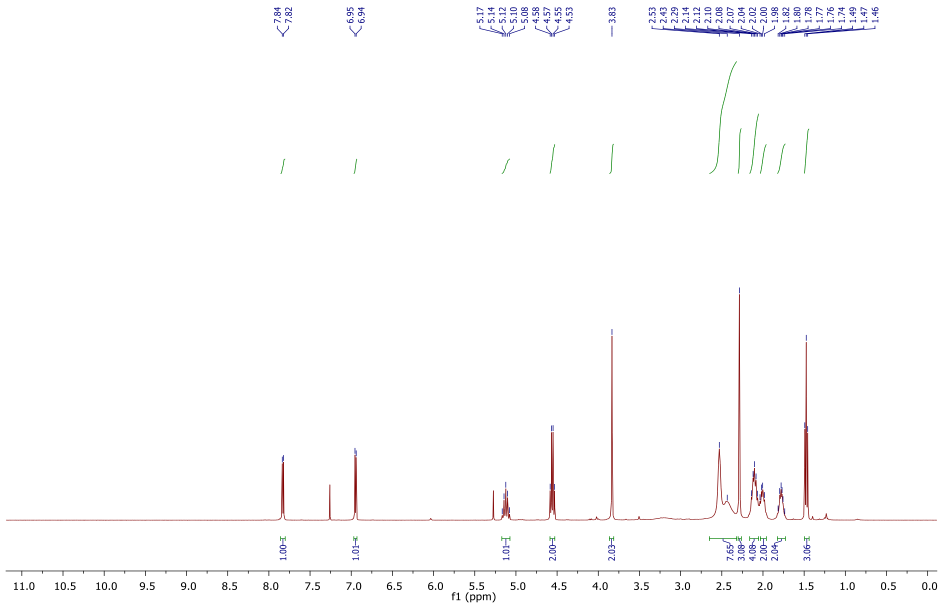
7.84
7.82
6.95
6.94
5.17
5.14
5.12
5.10
5.08
4.58
4.57
4.55
4.53
3.83
2.53
2.43
2.29
2.14
2.12
2.10
2.08
2.07
2.04
2.02
2.00
1.98
1.82
1.80
1.78
1.77
1.76
1.74
1.49
1.47
1.46
1.00
1.01
1.01
2.00
2.03
7.65
3.08
4.08
2.00
2.04
3.06
11.0 10.5 10.0 9.5 9.0 8.5 8.0 7.5 7.0 6.5 6.0 5.5 5.0 4.5 4.0 3.5 3.0 2.5 2.0 1.5 1.0 0.5 0.0
f1 (ppm)

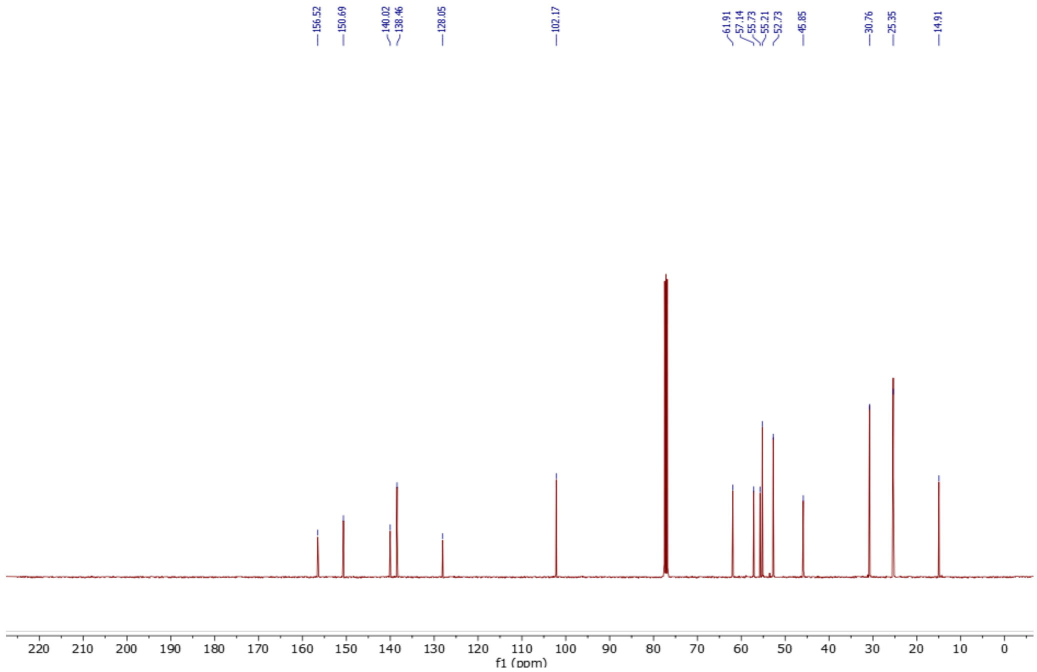
156.52
150.69
140.02
138.46
128.05
102.17
61.91
57.14
55.73
55.21
52.73
45.85
30.76
25.35
14.91
220 210 200 190 180 170 160 150 140 130 120 110 100 90 80 70 60 50 40 30 20 10 0
f1 (ppm)

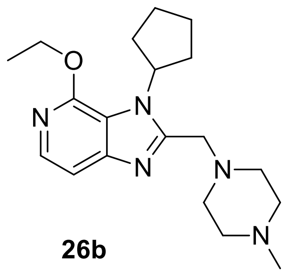
O
N
N
N
N
N

26b

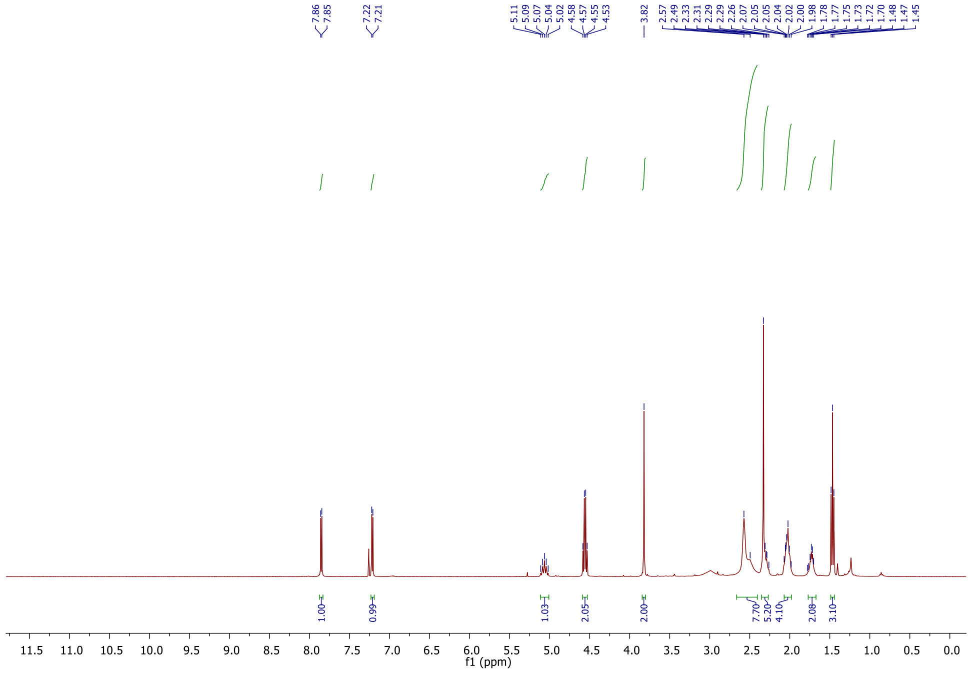
7.86
7.85
7.22
7.21
5.11
5.09
5.07
5.04
5.02
4.58
4.57
4.55
4.53
3.82
2.57
2.49
2.33
2.31
2.29
2.29
2.26
2.07
2.05
2.05
2.04
2.02
2.00
1.98
1.78
1.77
1.75
1.73
1.72
1.70
1.48
1.47
1.45
1.00
0.99
1.03
2.05
2.00
7.70
5.20
4.10
2.08
3.10
11.5 11.0 10.5 10.0 9.5 9.0 8.5 8.0 7.5 7.0 6.5 6.0 5.5 5.0 4.5 4.0 3.5 3.0 2.5 2.0 1.5 1.0 0.5 0.0
f1 (ppm)

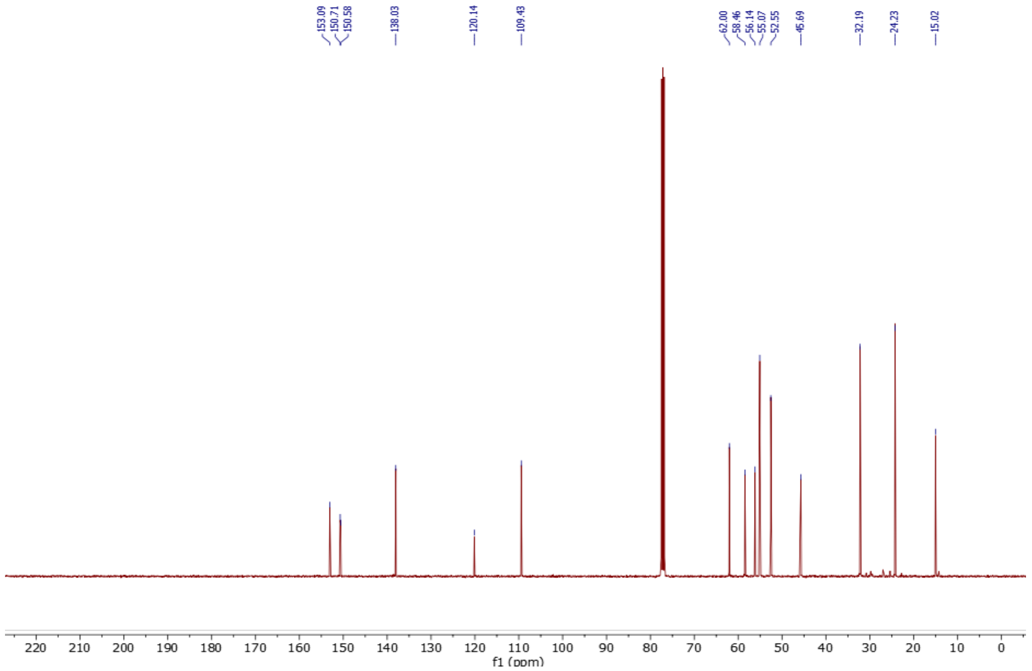
153.09
150.71
150.58
138.03
120.14
109.43
62.00
58.46
56.14
55.07
52.55
45.69
32.19
24.23
15.02
220 210 200 190 180 170 160 150 140 130 120 110 100 90 80 70 60 50 40 30 20 10 0
f1 (ppm)

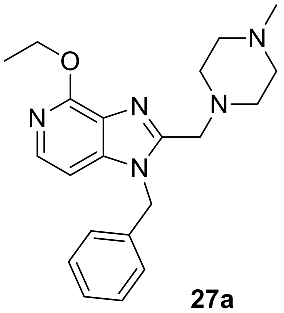
O
N
N
N
N
N

27a

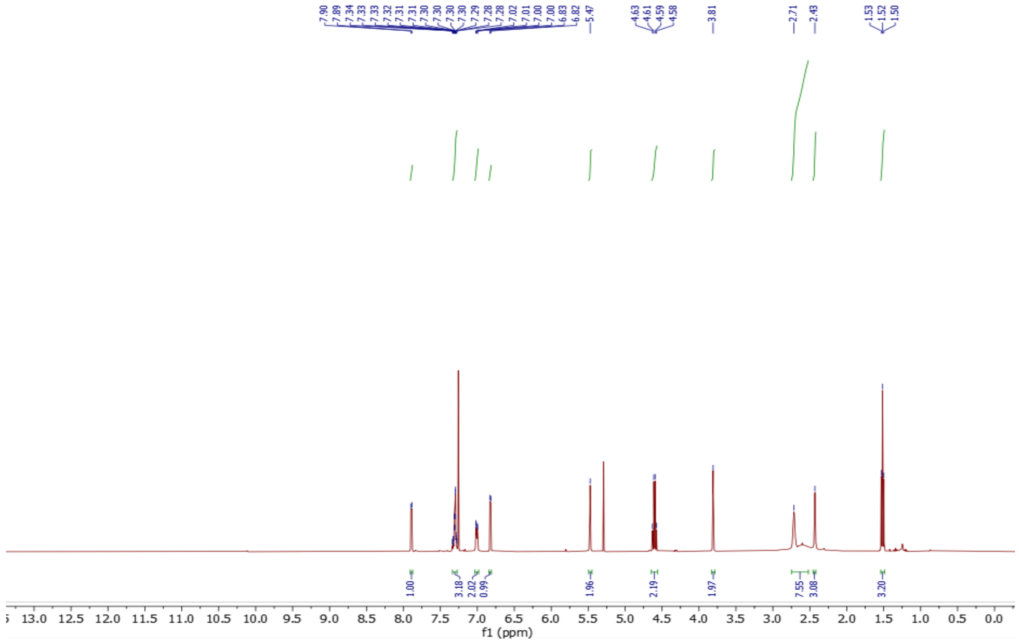
7.90
7.89
7.34
7.33
7.33
7.32
7.31
7.31
7.30
7.30
7.30
7.30
7.29
7.28
7.28
7.02
7.01
7.00
7.00
6.83
6.82
5.47
4.63
4.61
4.59
4.58
3.81
2.71
2.43
1.53
1.52
1.50
1.00
3.18
2.02
0.99
1.96
2.19
1.97
7.55
3.08
3.20
13.0 12.5 12.0 11.5 11.0 10.5 10.0 9.5 9.0 8.5 8.0 7.5 7.0 6.5 6.0 5.5 5.0 4.5 4.0 3.5 3.0 2.5 2.0 1.5 1.0 0.5 0.0
f1 (ppm)

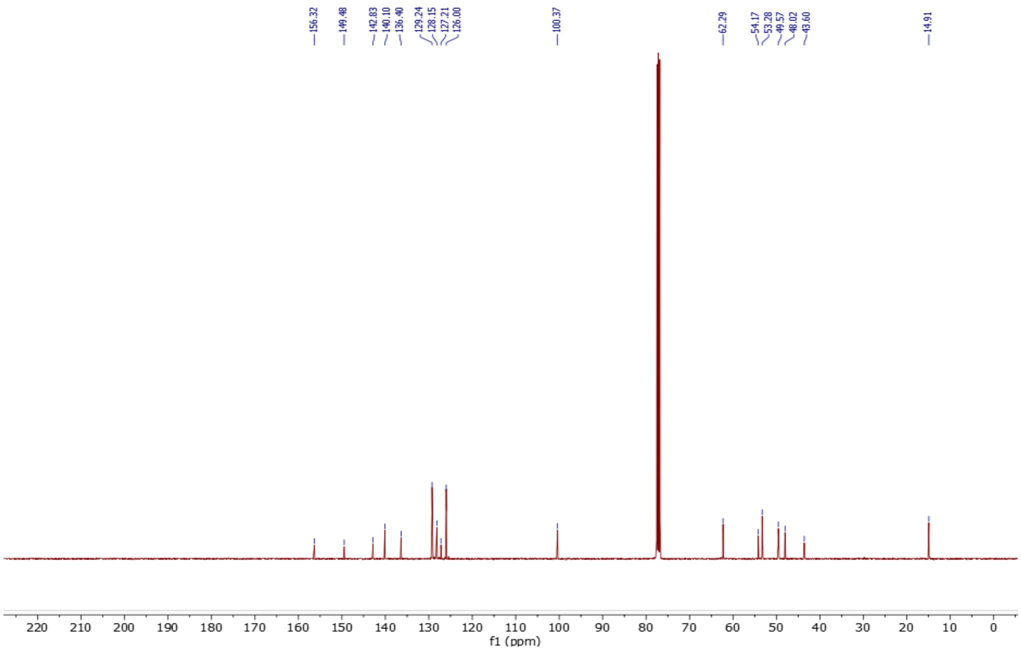
156.32
149.48
142.83
140.10
136.40
129.24
128.15
127.21
126.00
100.37
62.29
54.17
53.28
49.57
48.02
43.60
14.91
220 210 200 190 180 170 160 150 140 130 120 110 100 90 80 70 60 50 40 30 20 10 0
f1 (ppm)

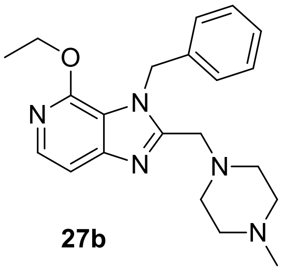

27b

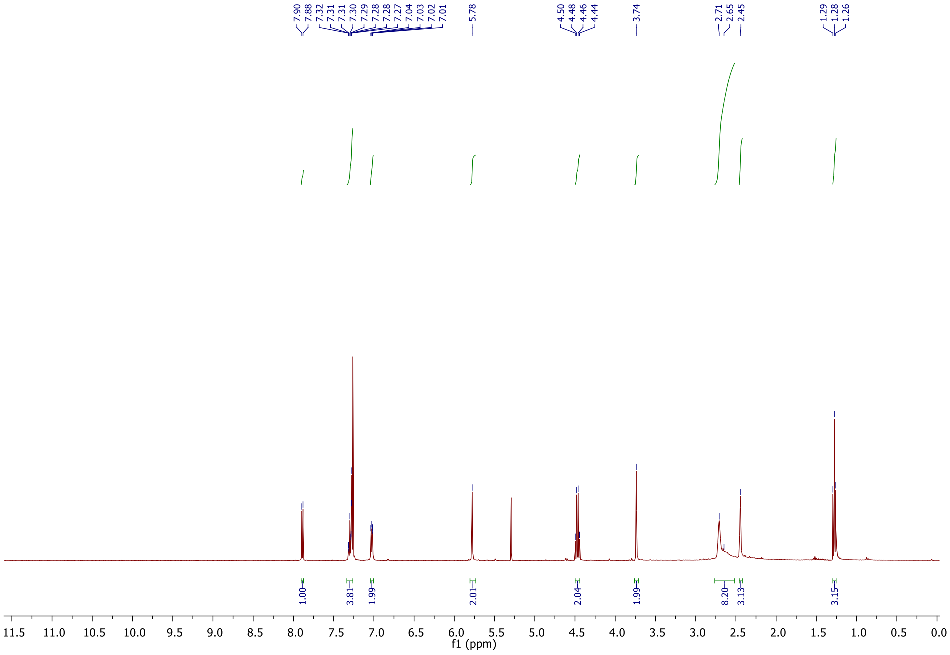

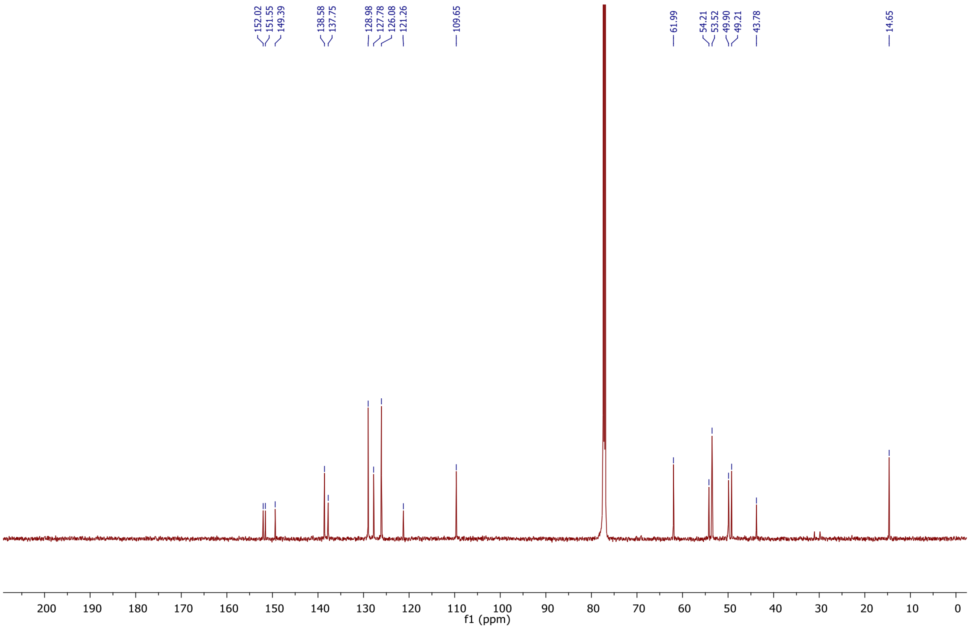

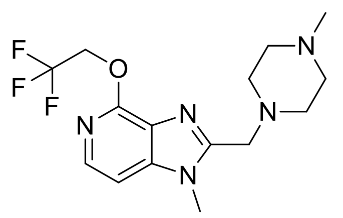

28a

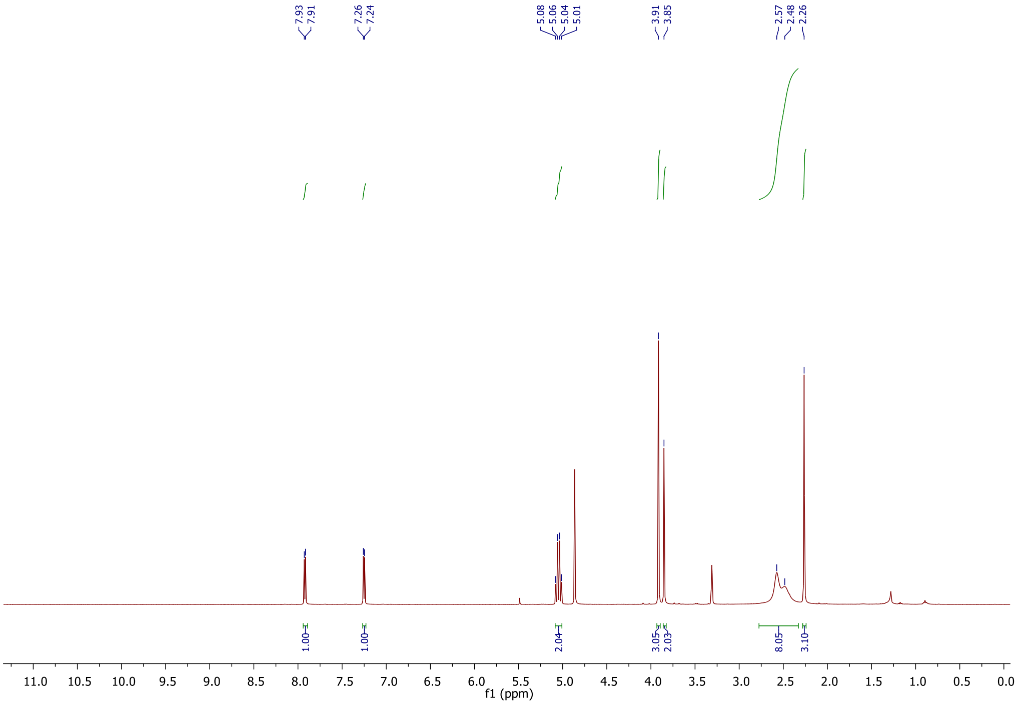
7.93
7.91
7.26
7.24
5.08
5.06
5.04
5.01
3.91
3.85
2.57
2.48
2.26
1.00
1.00
2.04
3.05
2.03
8.05
3.10
11.0 10.5 10.0 9.5 9.0 8.5 8.0 7.5 7.0 6.5 6.0 5.5 5.0 4.5 4.0 3.5 3.0 2.5 2.0 1.5 1.0 0.5 0.0
f1 (ppm)

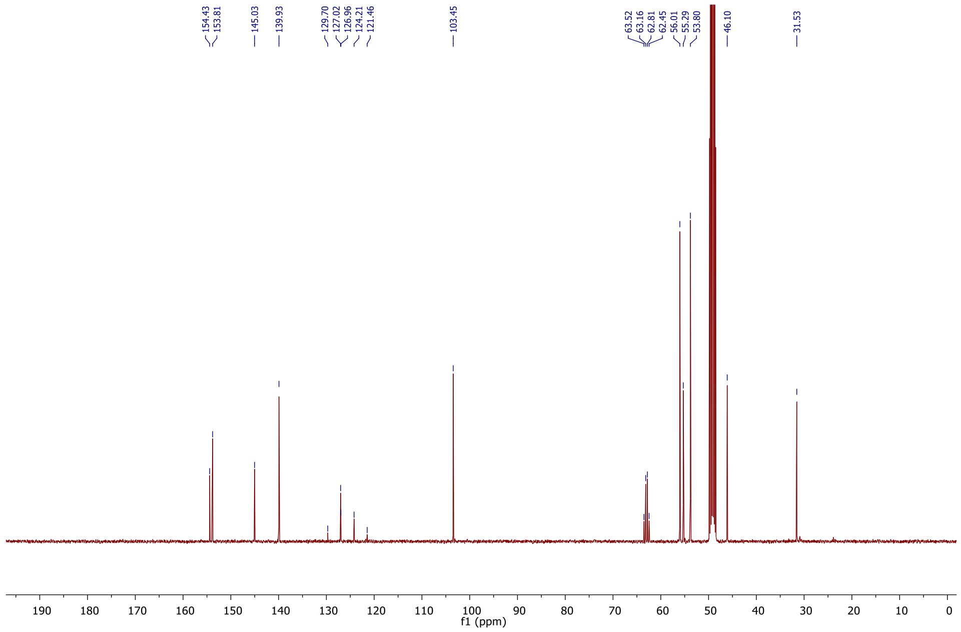
154.43
153.81
145.03
139.93
129.70
127.02
126.96
124.21
121.46
103.45
63.52
63.16
62.81
62.45
56.01
55.29
53.80
46.10
31.53
190 180 170 160 150 140 130 120 110 100 90 80 70 60 50 40 30 20 10 0
f1 (ppm)

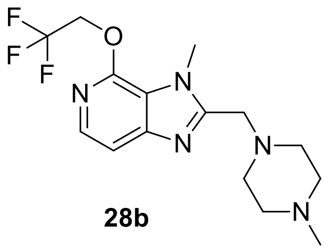
F
F
F
O
N
N
N
N
N

28b

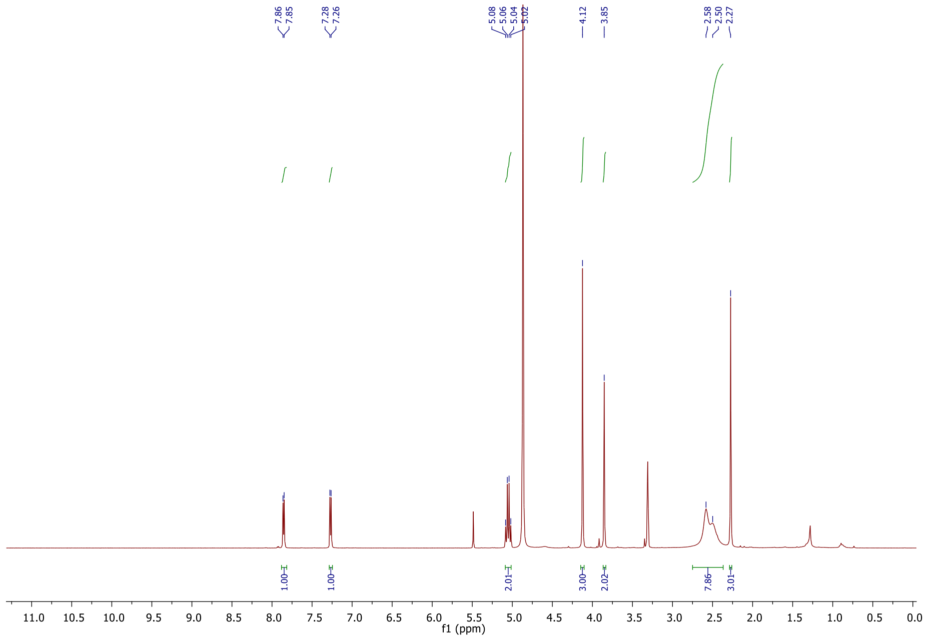
7.86
7.85
7.28
7.26
5.08
5.06
5.04
5.02
4.12
3.85
2.58
2.50
2.27
1.00
1.00
2.01
3.00
2.02
7.86
3.01
11.0 10.5 10.0 9.5 9.0 8.5 8.0 7.5 7.0 6.5 6.0 5.5 5.0 4.5 4.0 3.5 3.0 2.5 2.0 1.5 1.0 0.5 0.0
f1 (ppm)

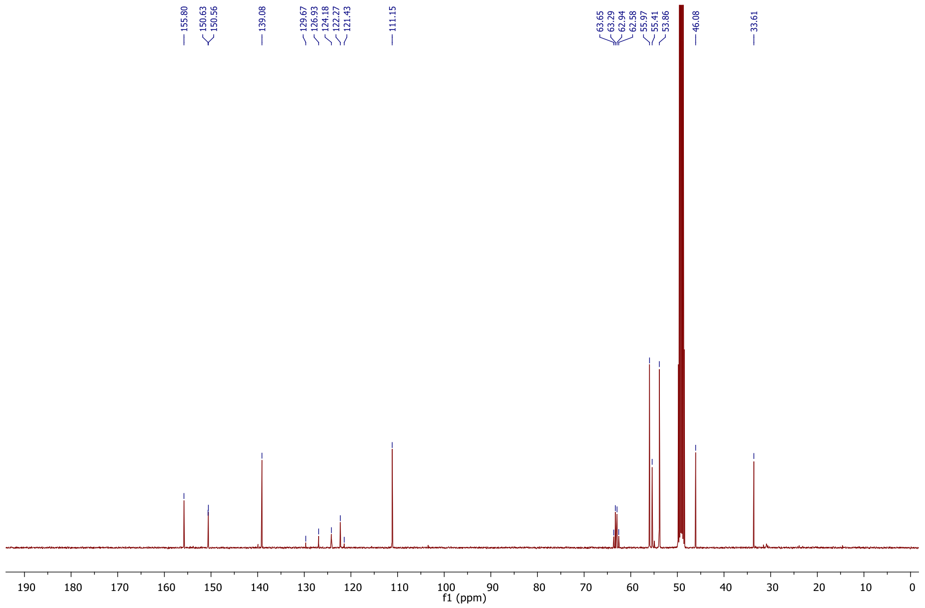
155.80
150.63
150.56
139.08
129.67
126.93
124.18
122.27
121.43
111.15
63.65
63.29
62.94
62.58
55.97
55.41
53.86
46.08
33.61
190 180 170 160 150 140 130 120 110 100 90 80 70 60 50 40 30 20 10 0
f1 (ppm)

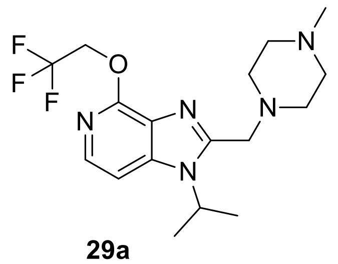
F
F
F
O
N
N
N
N
N

29a

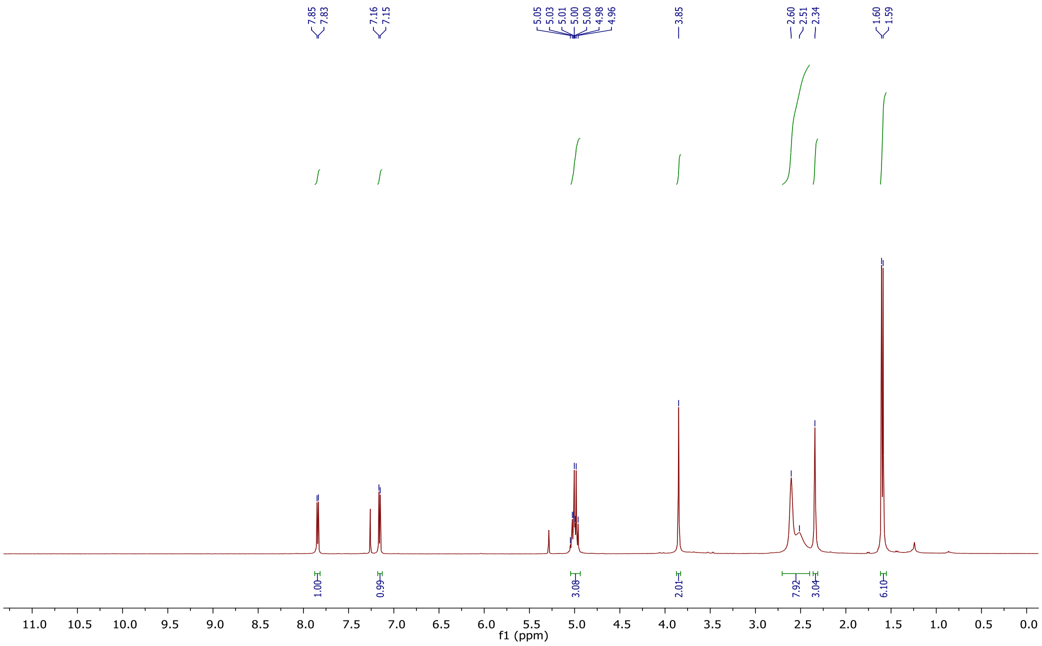
7.85
7.83
7.16
7.15
5.05
5.03
5.01
5.00
5.00
4.98
4.96
3.85
2.60
2.51
2.34
1.60
1.59
1.00
0.99
3.08
2.01
7.92
3.04
6.10
11.0 10.5 10.0 9.5 9.0 8.5 8.0 7.5 7.0 6.5 6.0 5.5 5.0 4.5 4.0 3.5 3.0 2.5 2.0 1.5 1.0 0.5 0.0
f1 (ppm)

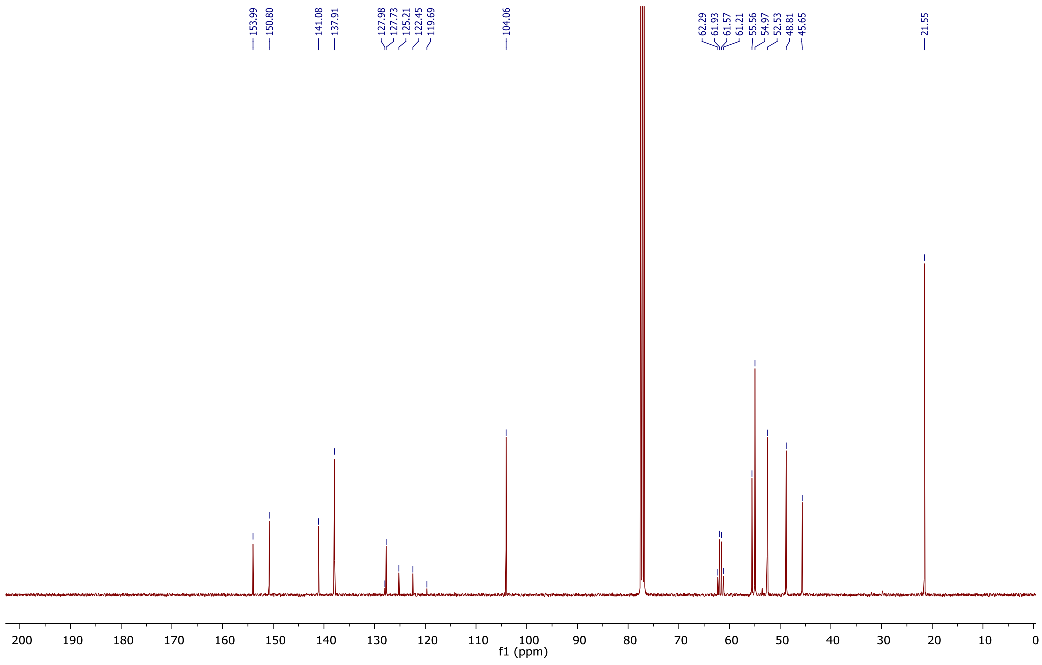
153.99
150.80
141.08
137.91
127.98
127.73
125.21
122.45
119.69
104.06
62.29
61.93
61.57
61.21
55.56
54.97
52.53
48.81
45.65
21.55
200 190 180 170 160 150 140 130 120 110 100 90 80 70 60 50 40 30 20 10 0
f1 (ppm)

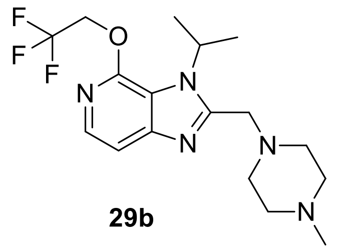

29b

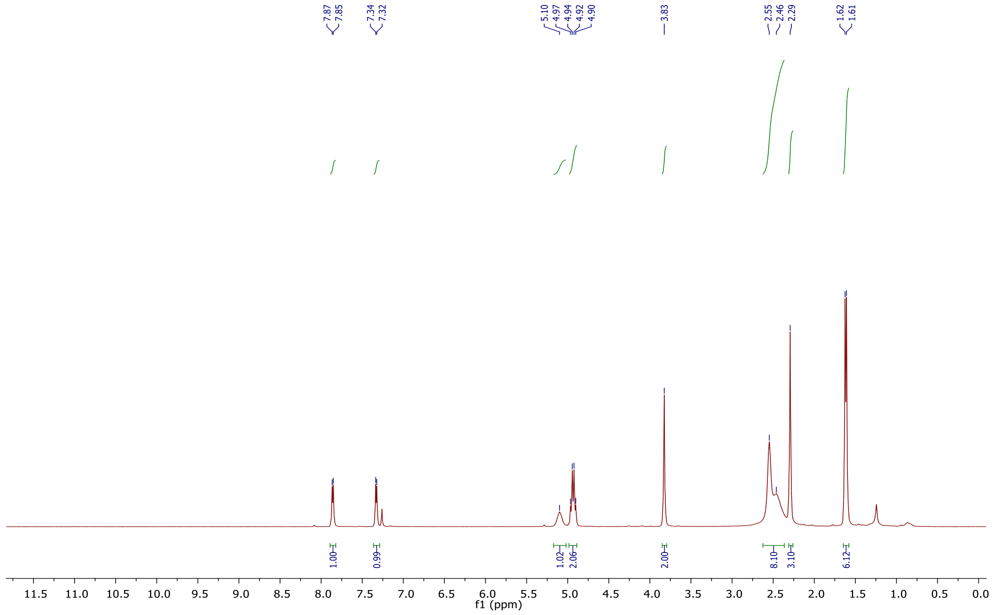
7.87
7.85
7.34
7.32
5.10
4.97
4.94
4.92
4.90
3.83
2.55
2.46
2.29
1.62
1.61
1.00
0.99
1.02
2.06
2.00
8.10
3.10
6.12
f1 (ppm)

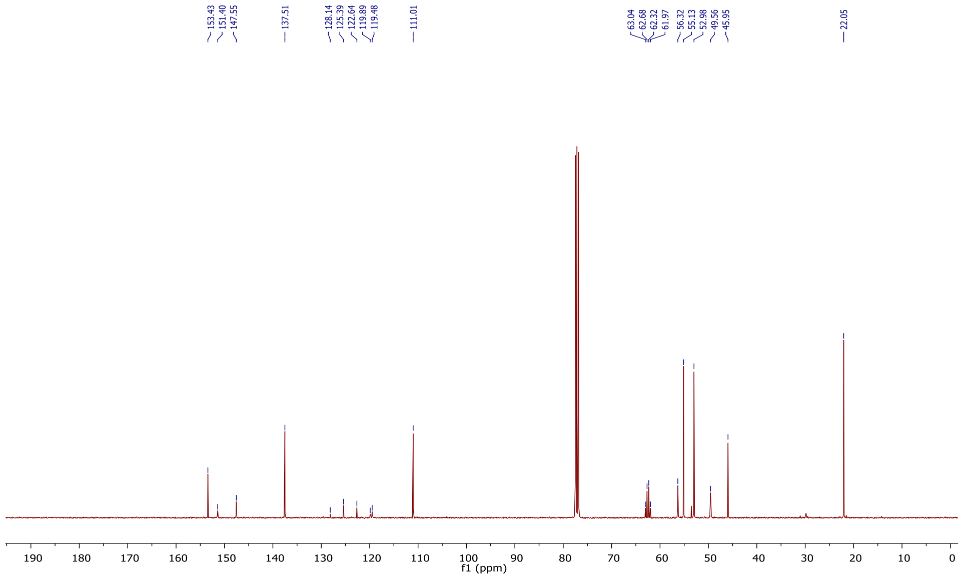
153.43
151.40
147.55
137.51
128.14
125.39
122.64
119.89
119.48
111.01
63.04
62.68
62.32
61.97
56.32
55.13
52.98
49.56
45.95
22.05
f1 (ppm)

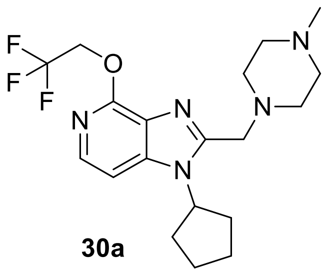

F
F
F
O
N
N
N
N
N

30a

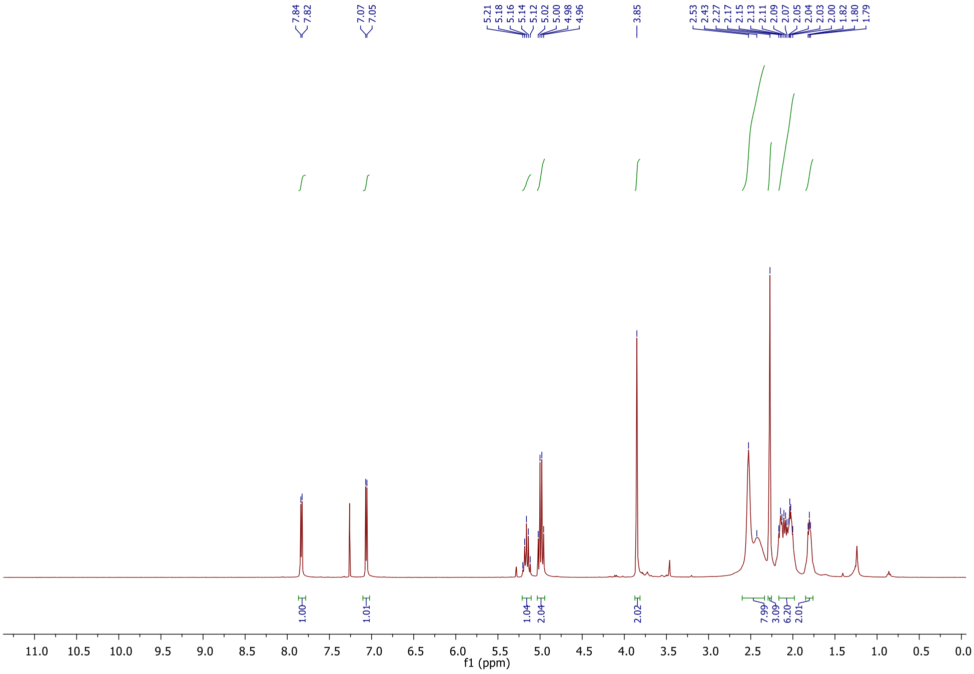

7.84
7.82
7.07
7.05
5.21
5.18
5.16
5.14
5.12
5.02
5.00
4.98
4.96
3.85
2.53
2.43
2.27
2.17
2.15
2.13
2.11
2.09
2.07
2.05
2.04
2.03
2.00
1.82
1.80
1.79
1.00
1.01
1.04
2.04
2.02
7.99
3.09
6.20
2.01
11.0 10.5 10.0 9.5 9.0 8.5 8.0 7.5 7.0 6.5 6.0 5.5 5.0 4.5 4.0 3.5 3.0 2.5 2.0 1.5 1.0 0.5 0.0
f1 (ppm)

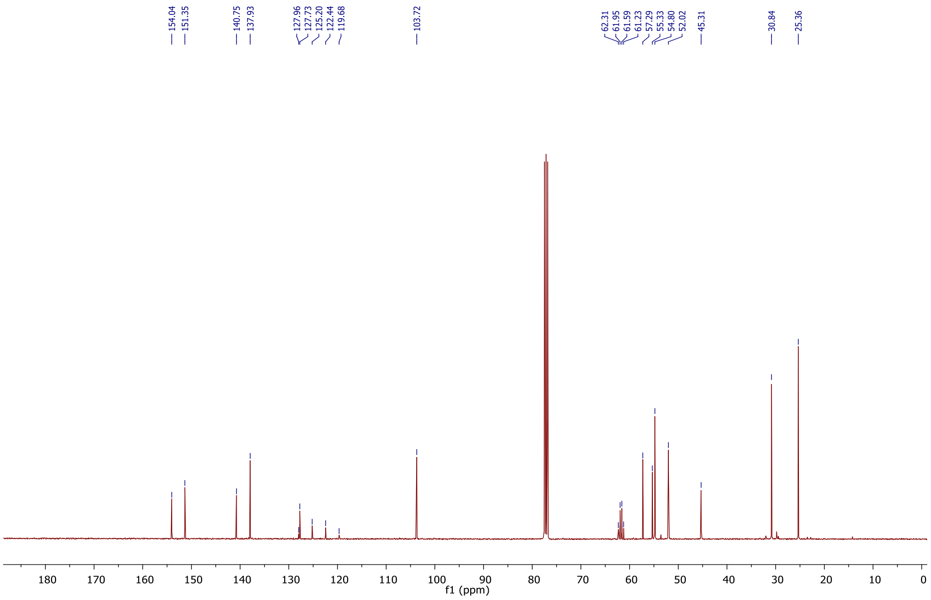

154.04
151.35
140.75
137.93
127.96
127.73
125.20
122.44
119.68
103.72
62.31
61.95
61.59
61.23
57.29
55.33
54.80
52.02
45.31
30.84
25.36
180 170 160 150 140 130 120 110 100 90 80 70 60 50 40 30 20 10 0
f1 (ppm)

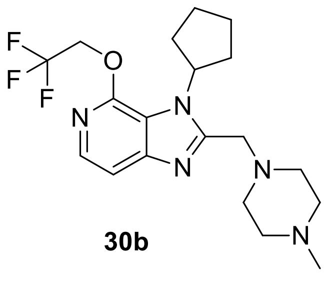

30b

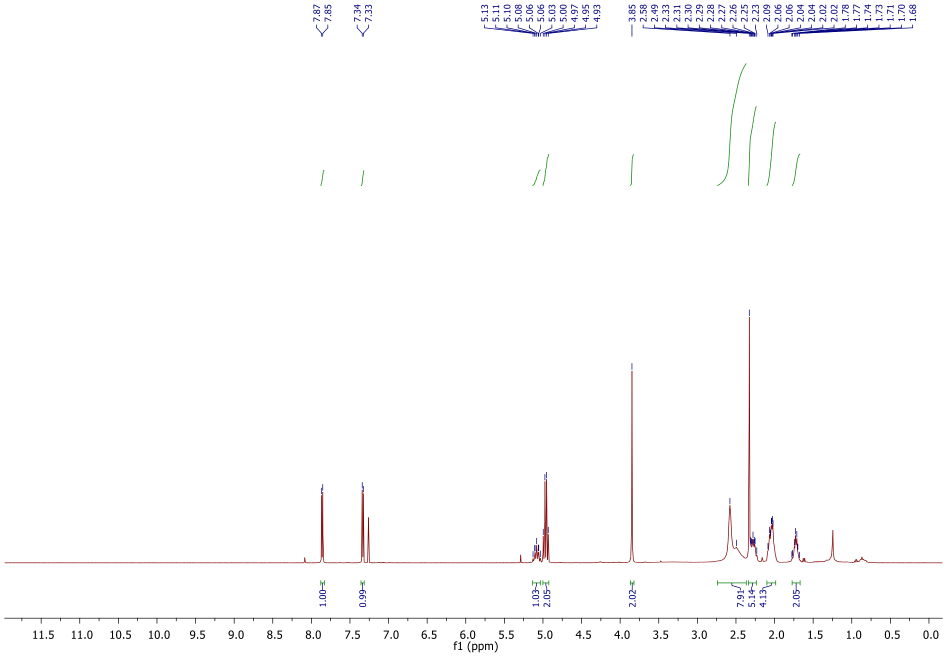
7.87
7.85
7.34
7.33
5.13
5.11
5.10
5.08
5.06
5.06
5.03
5.00
4.97
4.95
4.93
3.85
2.58
2.49
2.33
2.31
2.30
2.29
2.28
2.27
2.26
2.25
2.23
2.09
2.06
2.06
2.04
2.04
2.02
2.02
1.78
1.77
1.74
1.73
1.71
1.70
1.68
1.00
0.99
1.03
2.05
2.02
7.91
5.14
4.13
2.05
f1 (ppm)

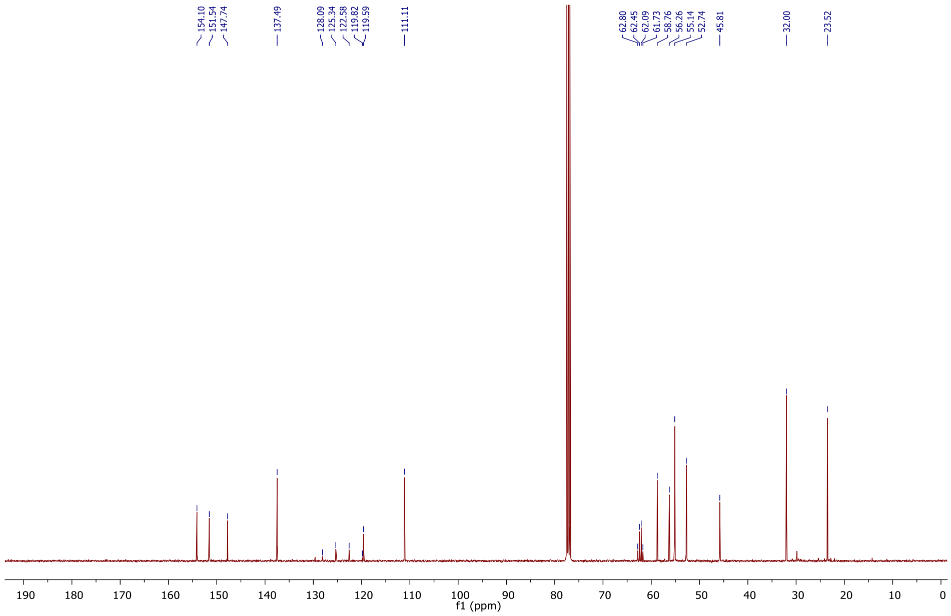
154.10
151.54
147.74
137.49
128.09
125.34
122.58
119.82
119.59
111.11
62.80
62.45
62.09
61.73
58.76
56.26
55.14
52.74
45.81
32.00
23.52
f1 (ppm)

Figure S2. Copies of the NOESY spectra of the target derivatives 25a, 25b, 26a, 26b, 30 and 30b.

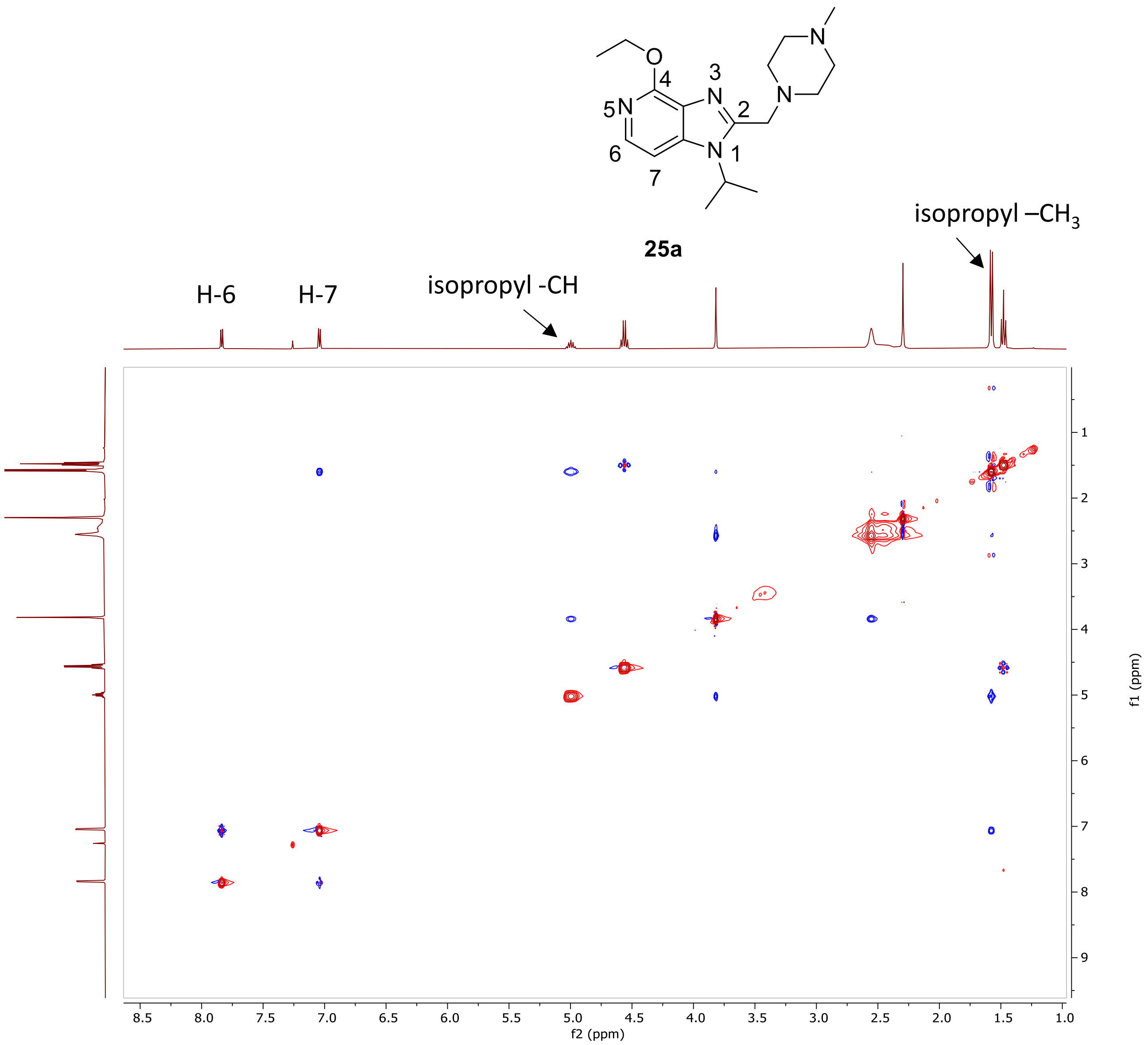

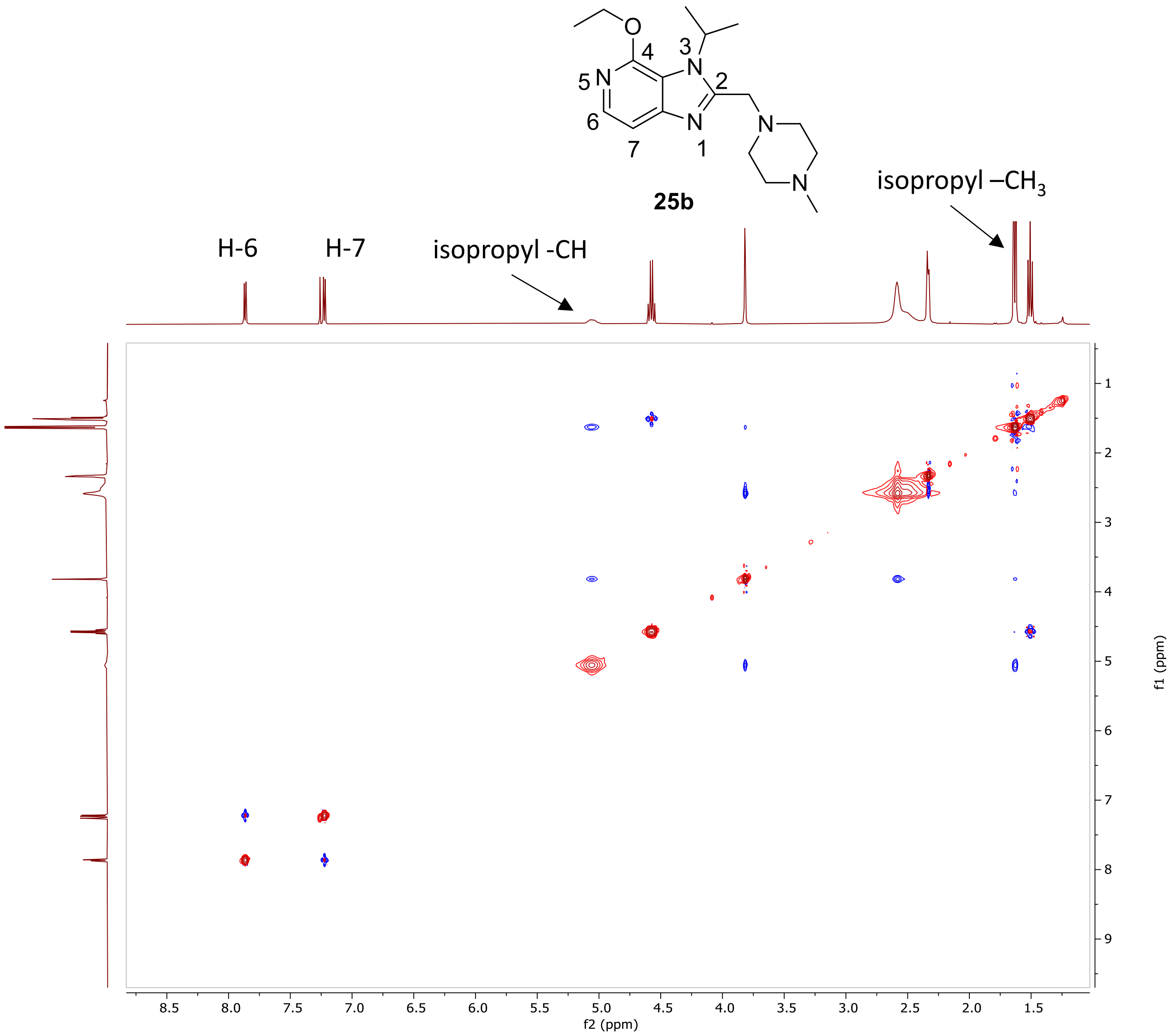
25b
H-6
H-7
isopropyl -CH
isopropyl –CH3
f1 (ppm)
f2 (ppm)

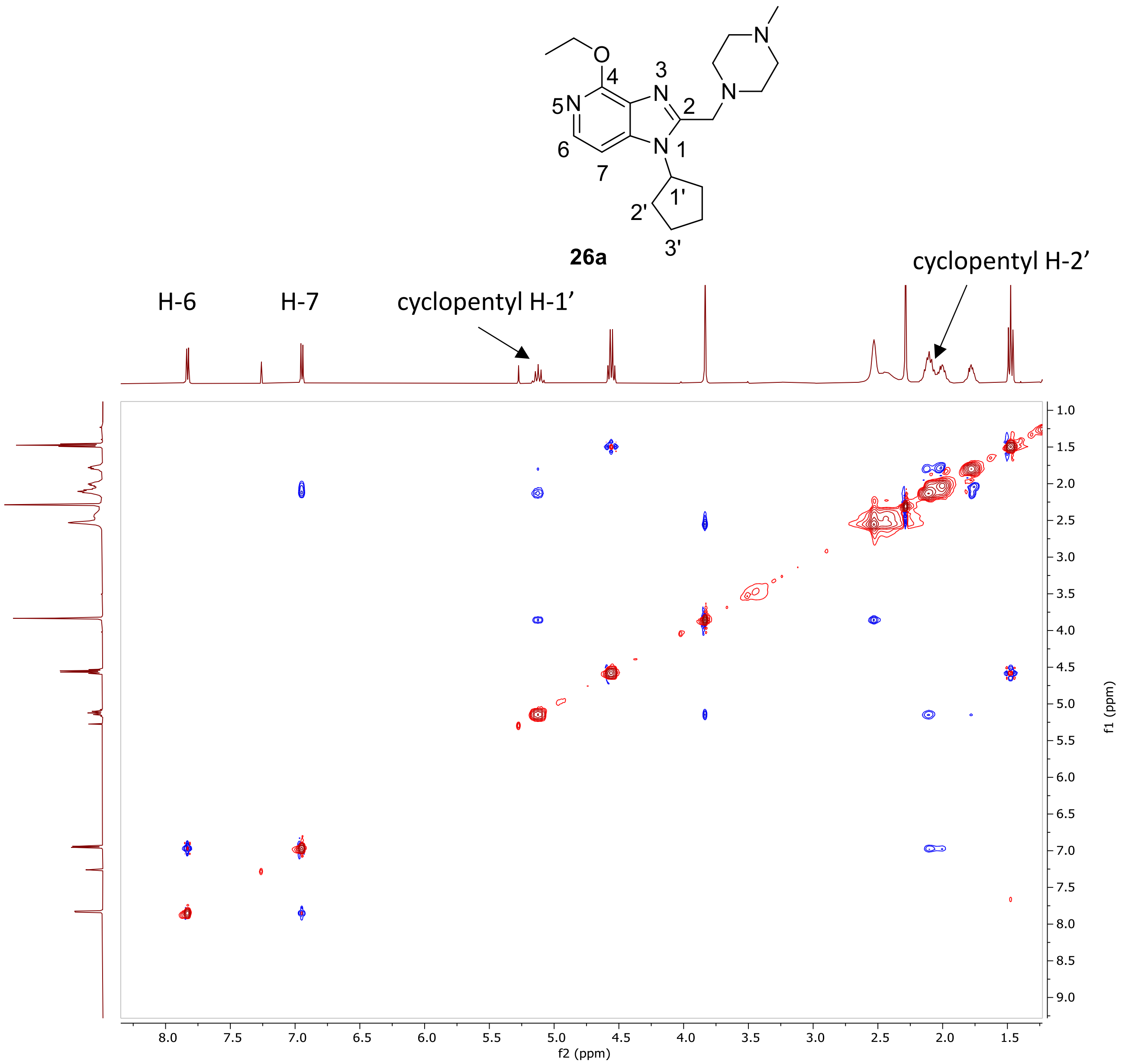

26a
H-6
H-7
cyclopentyl H-1'
cyclopentyl H-2'
f2 (ppm)
f1 (ppm)

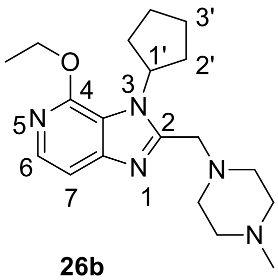

26b

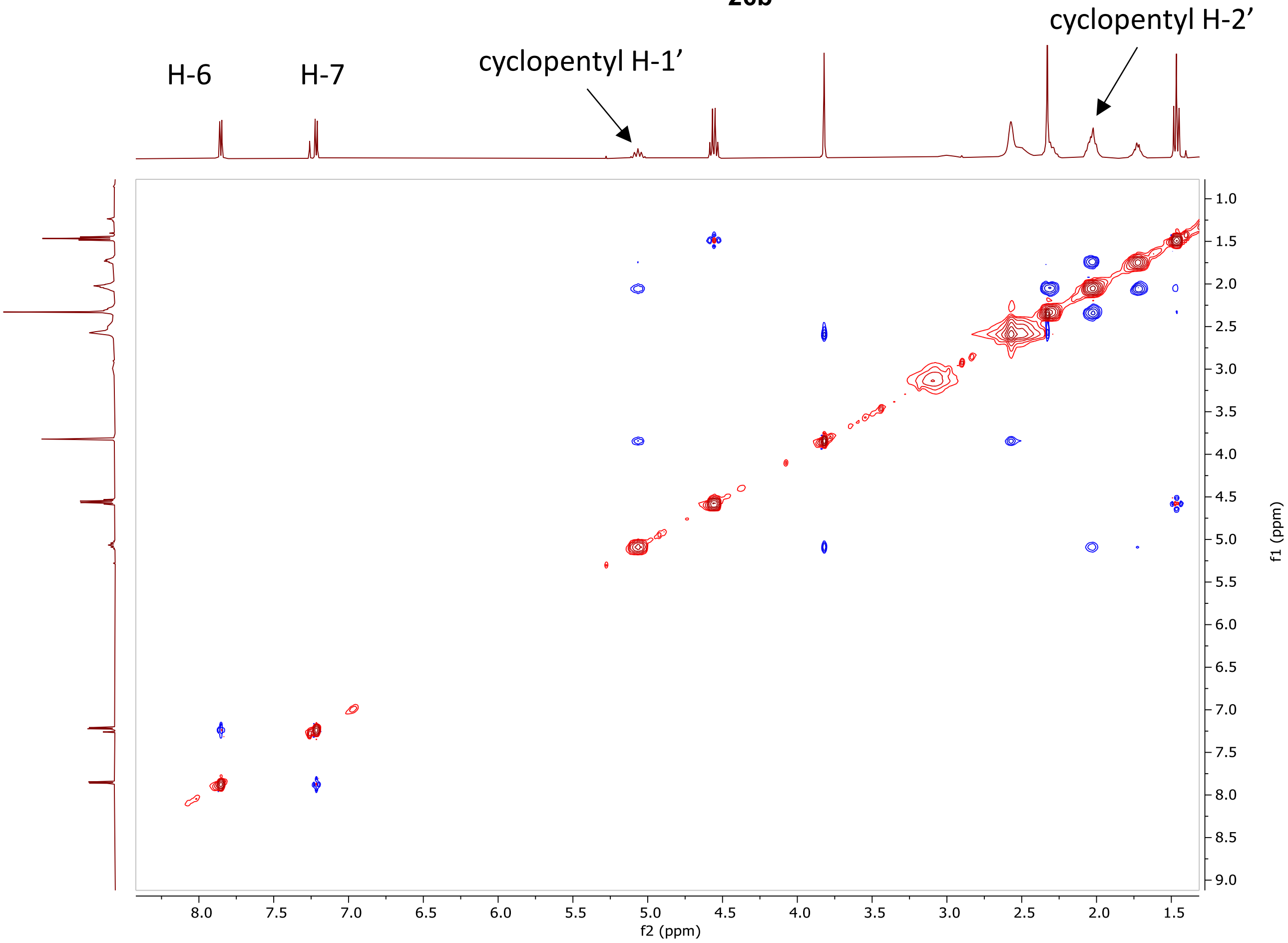

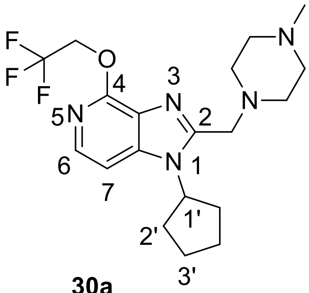
F
F
F
O
4
3
N
5N
2
N
N
N 1
6
7
1'
2'
3'

30a

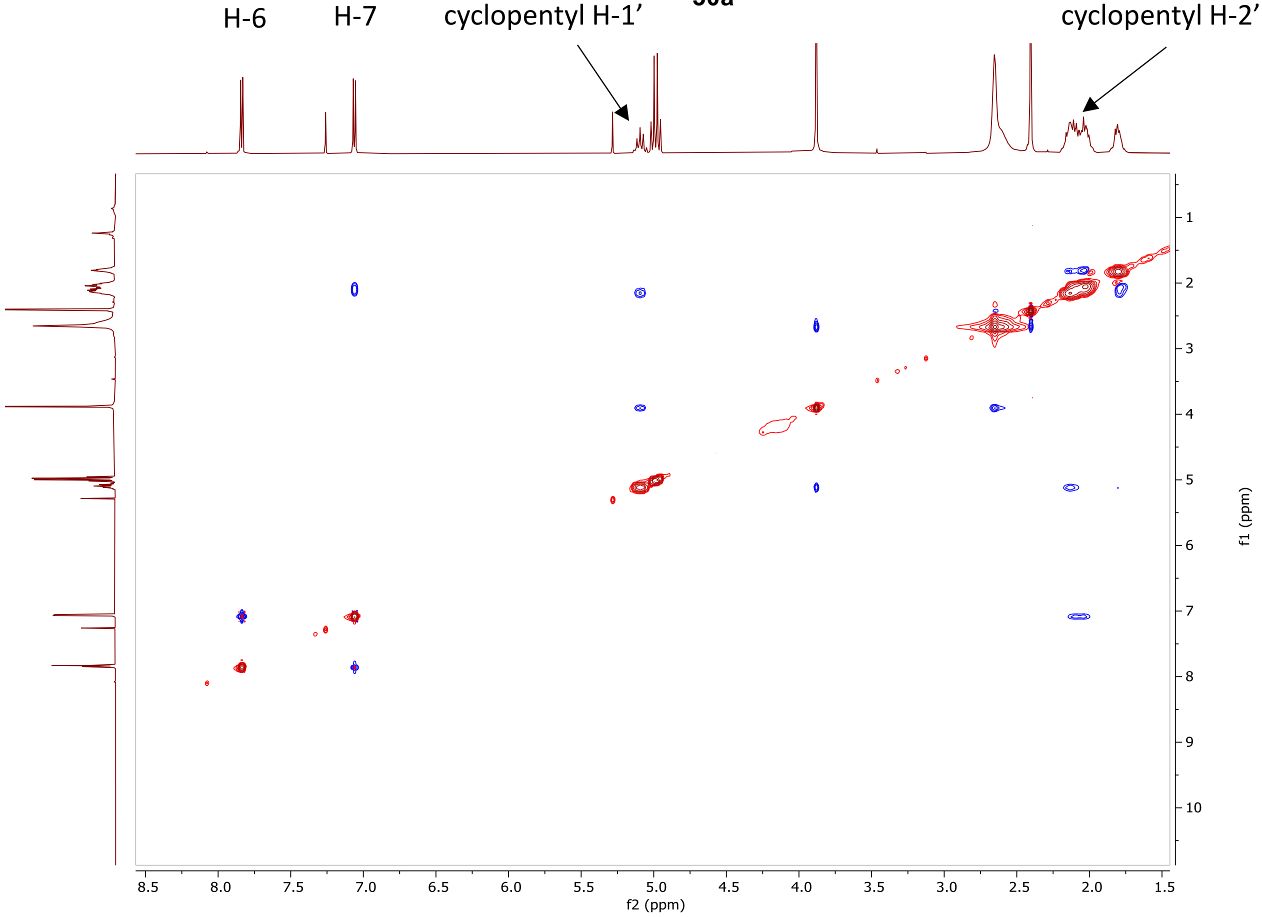
H-6
H-7
cyclopentyl H-1'
cyclopentyl H-2'
f2 (ppm)
f1 (ppm)
8.5
8.0
7.5
7.0
6.5
6.0
5.5
5.0
4.5
4.0
3.5
3.0
2.5
2.0
1.5
1
2
3
4
5
6
7
8
9
10

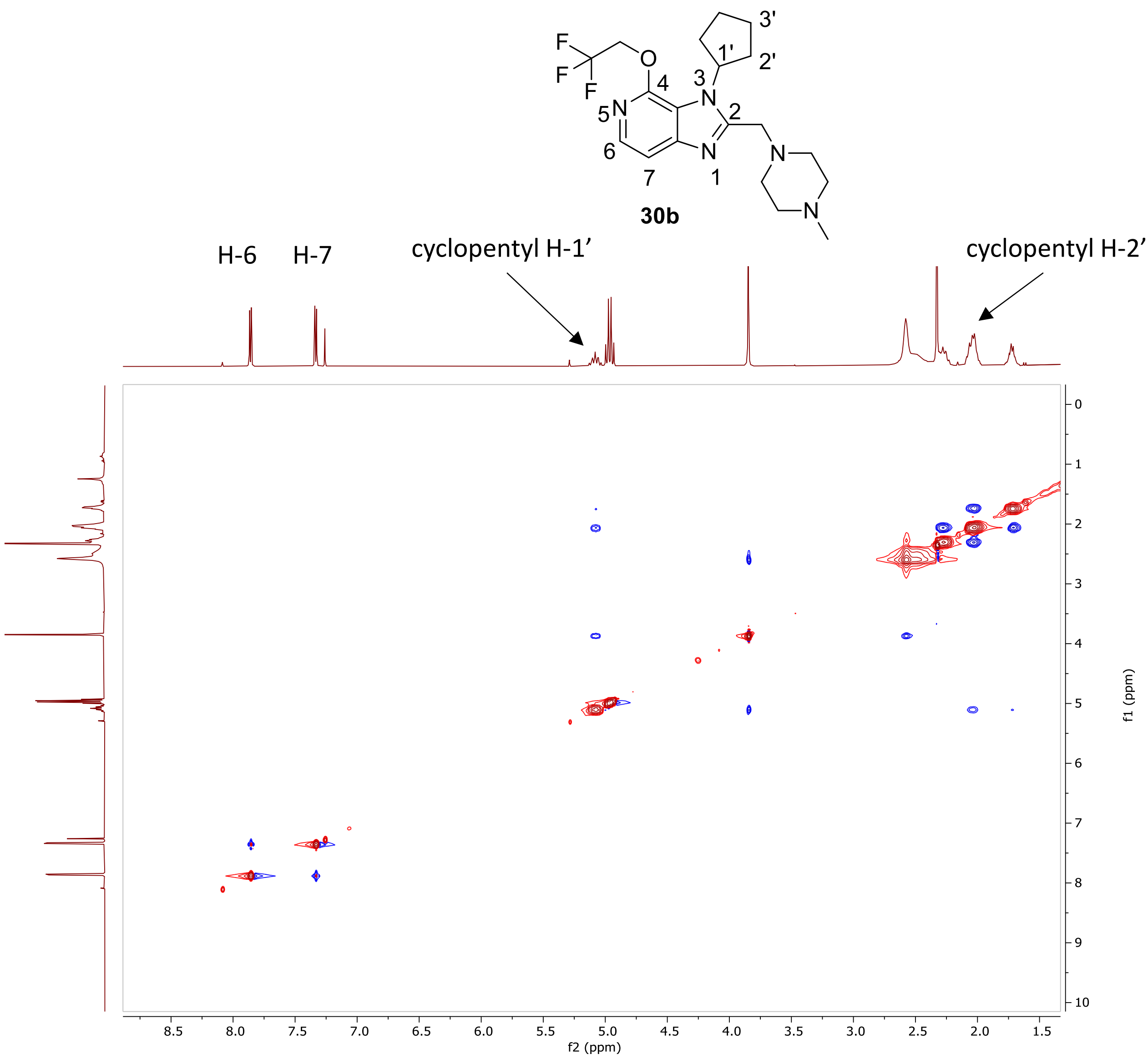
F
F
F
O
4
3
N
1'
3'
2'
5N
2
6
N
N
7
1
N
30b
H-6
H-7
cyclopentyl H-1’
cyclopentyl H-2’
f1 (ppm)
f2 (ppm)

Figure S3. Left panel: Docking pose of amphihevir A), anguizole B), and clemizole C) within the binding cavity of NS4B, Right panel: Close-up view of amphihevir, anguizole, and clemizole in the highest-scoring binding conformation of each compound with amino acid residues of NS4B protein. The orientation of each compound within the binding pocket of NS4B is illustrated, along with key interactions with surrounding residues that contribute to binding stabilization. (Red dashed lines represent hydrophobic interactions, while green dashed lines indicate hydrogen bonds).

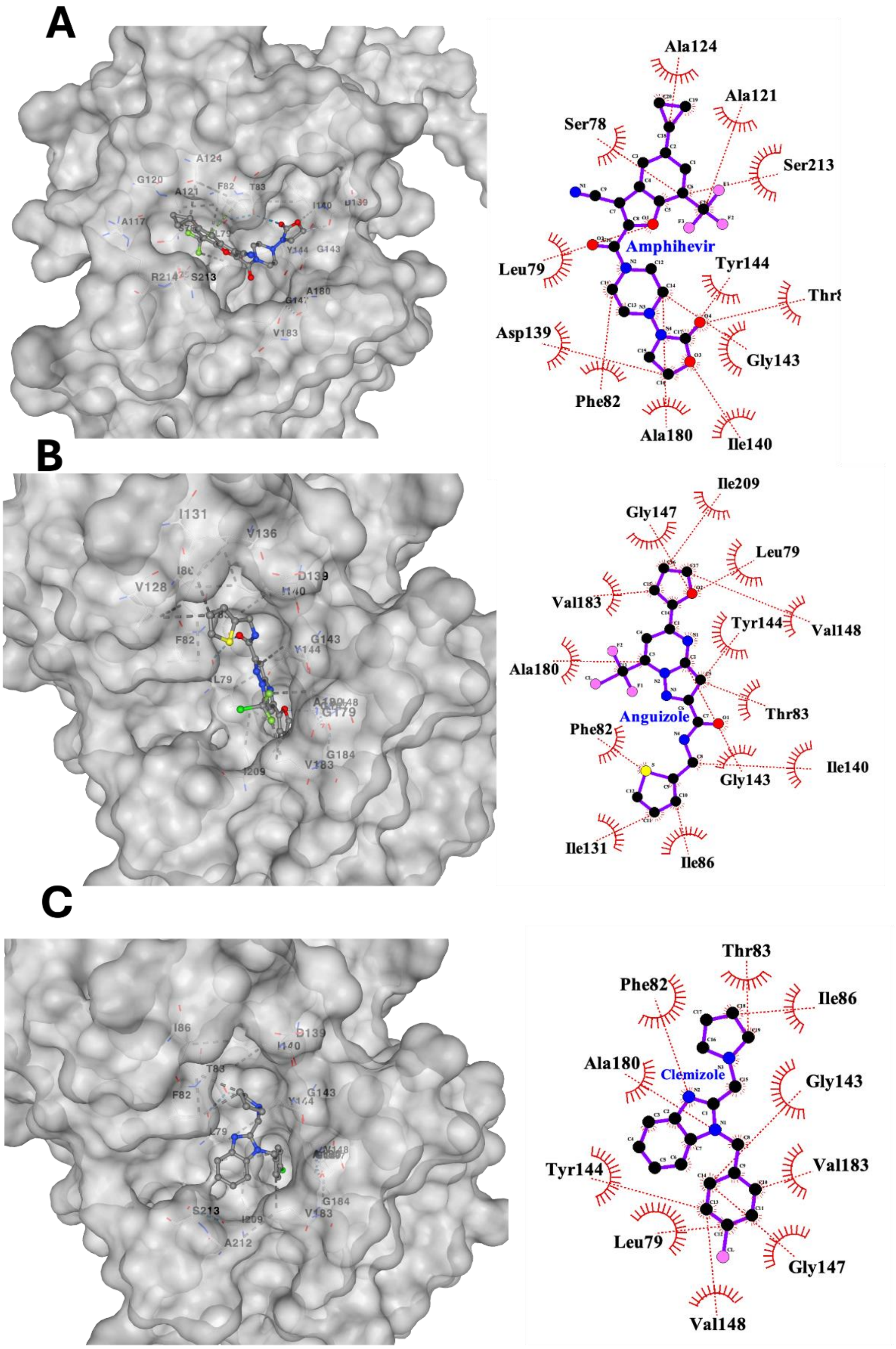

Supplement: Supplementary file 1 — Supplementary Material [file CMDC-21-e70378-s001.pdf]
